# N-doped porous carbons derived from Zn-porphyrin-MOF†

**DOI:** 10.1039/d2ra00327a

**Published:** 2022-02-18

**Authors:** Hyun-Chul Kim, Jongho Yoon, Sukbin Yoon, Youngmee Kim, Suk Joong Lee, Seong Huh

**Affiliations:** Department of Chemistry and Protein Research Center for Bio-Industry, Hankuk University of Foreign Studies Yongin 17035 Republic of Korea shuh@hufs.ac.kr +82 31 330 4566 +82 31 330 4522; Department of Chemistry, Research Institute for Natural Science, Korea University Seoul 136-701 Republic of Korea slee1@korea.ac.kr; Department of Chemistry and Nano Science, Ewha Womans University Seoul 120-750 Republic of Korea ymeekim@ewha.ac.kr

## Abstract

N-doped porous metal–organic framework (MOF)-derived carbons (MDCs) were directly synthesized from a new Zn-DpyDtolP-MOF (ZnDpyDtolP·1/2DMF, H_2_DpyDtolP = 5,15-di(4-pyridyl)-10,20-di(4-methylphenyl)porphyrin) containing a 3D hexagonal network through a self-templated carbonization method. KOH-activated MDC derivatives denoted as MDC-700-*n*KOH were also prepared with different weight ratios of KOH activator to MDC (MDC : KOH = 1 : *n*, where *n* = 1, 2). Compared to bare MDC, MDC-700-*n*KOH showed effective improvements of both gas sorption and electrochemical capacitive properties. More developed microporosity by KOH activation might induce great enhancement of high operating capacitive performances. The N-doped MDC-700-2KOH had high maximum gravimetric specific capacitance (555.6 F g^−1^) and specific energy (40.4 W h kg^−1^) at 0.1 A g^−1^ in 1 M H_2_SO_4_. Even at a high current density of 190 A g^−1^ in 6 M KOH, it exhibited high capacitive performance with a large specific power of 80 423 W kg^−1^. MDC-700-*n*KOH electrodes also showed good recycling properties of electrochemical capacitance up to 30 000 cycles.

## Abstract 2

The porphyrin-based Zn-MOF is directly carbonized and activated by KOH for the generation of N-doped porous carbons acting as high performance supercapacitor electrode materials.

## Graphical abstract

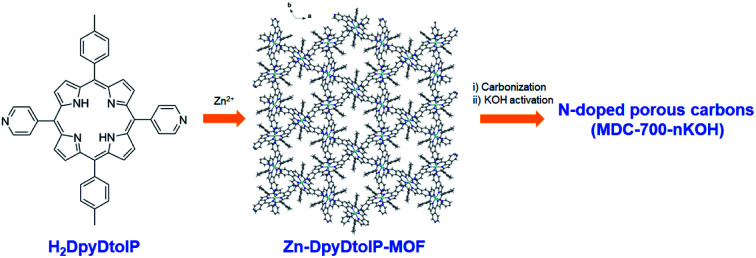

## 1. Introduction

Porphyrin-based metal–organic frameworks (por-MOFs) are very attractive crystalline porous materials owing to their potential for a wide range of applications.^1–3^ Their diverse structural variations are also interesting to those who are investigating new MOF structures. Many custom-made porphyrins can generate por-MOFs with interesting topological structures and unique properties.^4,5^ Very recently, Zhou *et al.* have reported a series of por-MOFs using Zr^IV^-based UiO-66 analogues.^6,7^ One of the advantages of using porphyrin bridging ligands might be their large physical dimensions. In addition, some por-MOFs exhibit very good porosity. The framework stability of por-MOFs is generally very high. For instance, single crystals of [Co(DpyDtolP)]_6_·12H_2_O MOF (H_2_DpyDtolP = 5,15-di(4-pyridyl)-10,20-di(4-methylphenyl)porphyrin) can maintain single crystallinity after heat treatment at a temperature as high as 250 °C.^8^ Analogous Co-por-MOFs show similar thermal stability.^9^ This phenomenal thermal stability can be mainly attributed to robust porphyrin-based bridging ligands. These exemplary cases clearly demonstrate that por-MOFs are robust crystalline porous solids.

MOFs are also excellent self-sacrificing templates for the preparation of porous carbon materials for diverse applications.^10–13^ Various porous carbons have functionalities suitable for heterogeneous catalysis, gas sorption, and electrical energy storage.^14,15^ It is worth mentioning that metal ions in MOFs often change their forms into oxides or sulfides nanoparticles embedded in carbon matrix depending on conditions of high temperature heat treatment.^12,16–19^ Thus, metal-free porous carbons can only be obtained after additional etching processes.^18,19^ In stark contrast, Zn-based MOFs tend to form porous carbons with very small amount of Zn species in resulting carbons without an additional etching process because Zn species are known to be volatile at high temperatures.^20^ In this sense, pure carbonaceous materials have been successfully prepared through a single step direct carbonization of Zn-MOFs.^20^ Furthermore, heteroatom-doping can occur simultaneously when Zn-MOFs contain heteroatom dopant sources. ZIF-8 can be carbonized to give N-doped porous carbons.^21^ Despite these promising results, direct carbonization of Zn-por-MOFs is relatively rare.

**Fig. 1:**
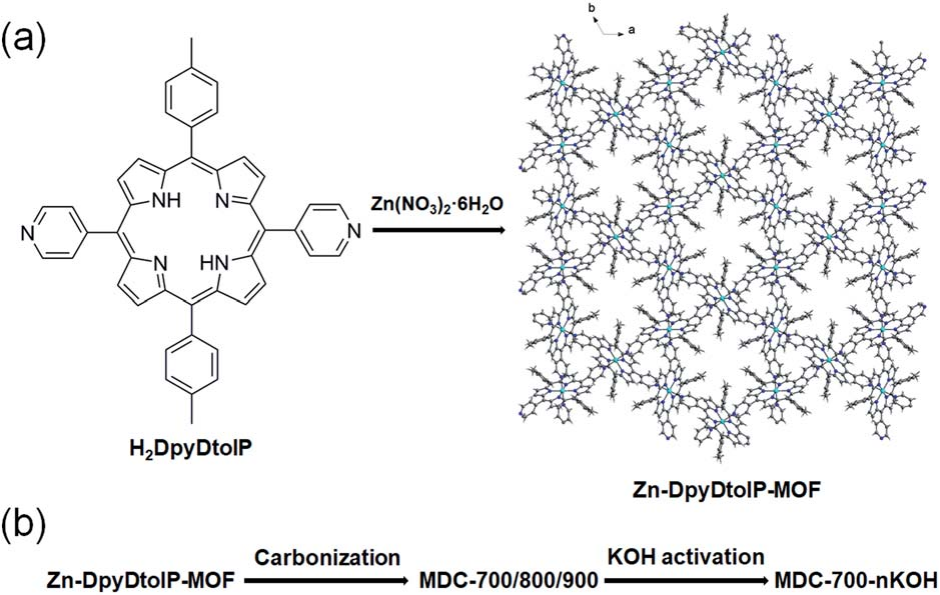
Illustration of the generation of Zn-DpyDtolP-MOF (ZnDpyDtolP·1/2DMF) (a) and the porous carbon materials by one-pot carbonization of as-prepared Zn-DpyDtolP-MOF (b).

We hypothesize that pyrrolic N atoms in porphyrin backbone could behave as N-dopants. In the case of DpyDtolP ligand, there are additional pyridyl N atoms as potential N-dopant sources. Therefore, we attempted to prepare N-doped porous carbons through direct carbonization of as-prepared Zn-DpyDtolP-MOF as illustrated in Fig. 1. We anticipated that N atoms in DpyDtolP ligand could be N-dopants and that residual Zn species could be a small amount due to volatility of Zn species at a high carbonization temperature. Gas sorption abilities and electrochemical supercapacitive properties of the resulting N-doped porous carbons and their activated forms were then systematically investigated.

## 2. Experimental methods

### 2.1. Preparation of Zn-DpyDtolP-MOF, ZnDpyDtolP·1/2DMF

A mixture containing Zn(NO_3_)_2_·6H_2_O (0.059 g, 0.2 mmol, Sigma-Aldrich) and 5,15-di(4-pyridyl)-10,20-di(4-methylphenyl)porphyrin (H_2_DpyDtolP, 0.064 g, 0.1 mmol) in *N*,*N*-dimethylformamide (DMF, 5 mL) was placed in a Teflon-lined high pressure bomb, sealed, and heated at 120 °C for 48 h. It was then cooled slowly to room temperature. Purple needles were retrieved by filtration, washed with DMF, and dried overnight in air (yield: 50 mg). Anal. calcd for C_91_H_67_N_13_OZn_2_ (1489.37): C, 73.38%; H, 4.53%; and N, 12.23%. Found: C, 73.45%; H, 4.23%; N, and 11.85%.

### 2.2. Preparation of porous carbon materials

As-prepared Zn-DpyDtolP-MOF crystals (100 mg) were directly carbonized under a continuous flow of ultrapure grade (99.999%) nitrogen gas at the target temperature in a tube furnace for 2 h (MDC-700 at 700 °C; MDC-800 at 800 °C; MDC-900 at 900 °C). To reach the target temperature of the furnace, the temperature was gradually increased at a ramping rate of 2 °C min^−1^ from room temperature. After carbonization, black MDCs were obtained (yield: 69 mg for MDC-700, 68 mg for MDC-800, and 64 mg for MDC-900). MDC-700-1KOH and MDC-700-2KOH were prepared by mixing MDC-700 (100 mg) and KOH (100 or 200 mg) by grinding for 30 min followed by heat treatment at 700 °C for 2 h under nitrogen gas. After KOH activation, black solids were neutralized with a 1 M HCl (50 mL) overnight. The final solid products were retrieved by filtration, washed with deionized water, and dried in an oven at 80 °C.

### 2.3. Gas sorption measurements

Cryogenic N_2_ adsorption/desorption analysis was performed using a Belsorp-miniII instrument at 77 K (BEL Japan). Samples were dried at 423 K under high vacuum for 2 h before measurements. Low pressure CO_2_ adsorption/desorption measurements were also performed using the same equipment at 196 K (2-propanol/dry ice bath) and 273 K (ice bath). Ultrapure grade (99.999%) CO_2_ gas was used for gas sorption experiments. A moisture trap was placed at the outlet of the CO_2_ gas cylinder to avoid moisture contamination during measurements. Volumetric H_2_ and CH_4_ sorption experiments were conducted at 77 K and 196 K, respectively, using the same equipment.

### 2.4. Capacitance measurements

A 3-electrode (3E) cell with a Pt wire (Bioanalytical Systems, Germany), an Ag/AgCl (NaCl, 3 M) electrode (ALS, Japan), and a glassy carbon electrode (CHI 104, 3 mm in diameter, 0.0707 cm^2^, from CH Instruments, USA) embedded in Kel-F (polychlorotrifluoroethylene, PCTFE) were used as counter, reference, and working electrodes, respectively. The glassy carbon electrode was polished with an emery paper. Carbon samples were ground in an agate mortar prior to testing. The ground carbon sample (2 mg) was dispersed in 400 μL of deionized water and then ultrasonicated. Then 10 μL (containing 0.05 mg of the carbon sample) of the above suspension was dropped on the polished glassy carbon electrode and dried at room temperature for 1 h. After coating 20 μL of 5 wt% Nafion solution on the sample as a binder, the sample was dried at room temperature for 30 min. Before electrochemical measurements, the working electrode was dipped into an electrochemical cell containing 10 mL of electrolyte solution. Electrochemical tests including cyclic voltammetry (CV) and galvanostatic charge/discharge (GCD) cycling were performed in 1 M Na_2_SO_4_ solution at room temperature. A CompactStat potentiostat/galvanostat (Ivium Technology, Holland) was used for electrochemical tests. The specific capacitance was calculated from the area of the second cycle curve of the cyclic voltammogram. Alternatively, the specific capacitance was obtained in the GCD cycling mode based on the average value from the second to the fourth cycle of discharge curves.

### 2.5. Physical measurements

Powder X-ray diffraction (PXRD) patterns were obtained using a Bruker New D8 Advance diffractometer (40 kV, 40 mA, step size = 0.02°). SEM images were recorded using Coxem CX-100S (accelerating voltage = 20 kV) with metallic coating. X-ray photoelectron spectroscopy (XPS) measurements were performed using an ESCA 2000 equipped with Al Kα as an X-ray source. Micro Raman measurements were performed in backscattering geometry using a Horiba Jobin Yvon LabRam HR system fitted with a liquid-nitrogen cooled CCD multichannel detector. Elemental analysis was performed using an EA1112 (CE Instruments, Italy) analyzer. The crystal structure of Zn-DpyDtolP-MOF was analyzed with a Bruker APEX-II diffractometer equipped with a monochromator using a Mo Kα (*λ* = 0.71073 Å) incident beam. Crystallographic parameters and data are summarized in Tables S1–S5 (ESI, CCDC 956817†).

## 3. Results and discussion

### 3.1. Preparation of Zn-DpyDtolP-MOF

The new 3D Zn-DpyDtolP-MOF was assembled from a reaction between Zn(NO_3_)_2_·6H_2_O and the porphyrin-based ditopic H_2_DpyDtolP bridging linker in DMF at 120 °C. The H_2_DpyDtolP bridging linker contains two pyridyl functional groups at 5- and 15-positions ideal for bridging metal ions. After the reaction, uniform purple needles good for crystal structure determination formed (Fig. S1, ESI†). The crystal structure of Zn-DpyDtolP-MOF is isostructural with a previously reported Co analogue, Co-DpyDtolP-MOF.^8^ The asymmetric unit of the 3D framework structure of Zn-DpyDtolP-MOF is depicted in Fig. S2 (ESI†). There are hexagonally oriented microporous channels along the *c*-axis as shown in Fig. 1a. Both elemental analysis and thermogravimetric analysis (TGA) data indicated that the formula of Zn-DpyDtolP-MOF would be ZnDpyDtolP·1/2DMF. In the TGA curve shown in Fig. S3 (ESI†), a weight loss of 3.40% at 532 °C for the removal of DMF hemisolvate was clearly seen. With increasing temperature, further weight loss occurred possibly due to thermal decomposition of DpyDtolP ligand. The bulk purity of as-prepared Zn-DpyDtolP-MOF crystals was investigated by comparing the PXRD pattern of as-prepared sample with the simulated pattern from X-ray crystallographic data shown in Fig. S4 (ESI†). Main diffraction planes in the low angle region such as (2−10), (300), (101), (4−20), (20−1), and (4−1−1) were observed at 2*θ* values of 5.30, 9.22, 9.92, 10.62, 11.22, and 14.54°, respectively. Corresponding calculated 2*θ* values were 5.36, 9.30, 10.00, 10.74, 11.36, and 14.70°, respectively. Based on these observations, the purity of as-prepared Zn-DpyDtolP-MOF was very high.

### 3.2. Gas sorption properties of Zn-DpyDtolP-MOF

**Fig. 2:**
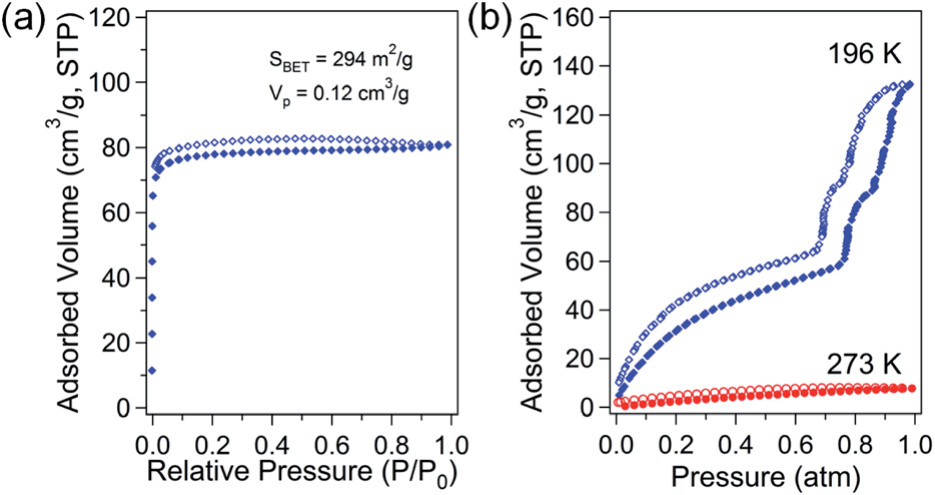
N_2_ adsorption/desorption isotherms of solvent-free Zn-DpyDtolP-MOF at 77 K (a). CO_2_ adsorption/desorption isotherms at 196 K and 273 K (b). Open and solid symbols indicate adsorption and desorption data, respectively.

The porosity of solvent-free Zn-DpyDtolP-MOF was systematically investigated by gas adsorption/desorption analysis using N_2_, H_2_, CH_4_, and CO_2_ gases at suitable temperatures. The microporous nature of Zn-DpyDtolP-MOF was clearly confirmed by N_2_ adsorption/desorption isotherms measured at 77 K (Fig. 2a). The typical type I adsorption isotherm was observed.^22^ There was a very small hysteresis between adsorption and desorption branches. The corresponding Brunauer–Emmett–Teller (BET) surface area was 294 m^2^ g^−1^ (0 < *P*/*P*_0_ < 0.1194) with a total pore volume of 0.12 cm^3^ g^−1^. The Horváth–Kawazoe (HK) micropore dimension was 0.81 nm (Fig. S5, ESI†). When H_2_ and CH_4_ sorption experiments were performed at 77 and 196 K, respectively, the adsorption capacity was 66.4 cm^3^ g^−1^ (2.96 mmol g^−1^, 0.60 wt%) for H_2_ and 21.4 cm^3^ g^−1^ (0.95 mmol g^−1^) for CH_4_ (Fig. S6, ESI†). The higher sorption for H_2_ might be mainly attributed to the small pore dimension of Zn-DpyDtolP-MOF. The kinetic diameter of H_2_ (2.89 Å) is much smaller than that of CH_4_ (3.80 Å).^23^ CO_2_ adsorption at 196 K showed a very interesting isotherm shape with a sorption amount of 132.3 cm^3^ g^−1^ (5.90 mmol g^−1^) as shown in Fig. 2b. In the adsorption branch, there was a very large step starting at 0.77 atm. An additional small step was also observed at 0.86 atm. This unique adsorption behavior was also observed for Co-DpyDtolP-MOF. It can be ascribed to the stepwise adsorption of CO_2_ molecules into microporous channels upon increase of gas pressure. Since the framework of Zn-DpyDtolP-MOF was very rigid, this sequential adsorption of CO_2_ molecules did not originate from breathing of channels. Slight hysteric behavior was observed for the desorption branch. The CO_2_ sorption behavior was also very closely related to the measurement temperature. The CO_2_ sorption amount at 273 K was merely 7.7 cm^3^ g^−1^ (0.35 mmol g^−1^). Thus, the CO_2_ sorption was not efficient at 273 K.

### 3.3. Direct carbonization of Zn-DpyDtolP-MOF

To prepare N-doped porous carbon directly from the porphyrin-based MOF sacrificial self-template, the as-prepared Zn-DpyDtolP-MOF was carbonized under inert atmosphere at three different temperatures (MDCs: MDC-700 at 700 °C, MDC-800 at 800 °C, and MDC-900 at 900 °C) to generate N-doped MOF-derived carbons. The porphyrin-based MOF self-template is known to produce porous carbons for a wide range of applications.^13^ The porosity of each MDC was investigated by N_2_ adsorption/desorption analysis at 77 K. Interestingly, no MDC showed any meaningful adsorption of N_2_. The kinetic diameter of N_2_ is 3.64 Å.^23^ We speculate that carbonization of Zn-DpyDtolP-MOF might have produced carbons with very small pore dimensions. This could be corroborated by adsorption experiments with CO_2_. Despite relatively low adsorption amounts of CO_2_, all MDCs exhibited CO_2_ adsorption abilities (Fig. S7, ESI†). The adsorption volume gradually decreased as the measurement temperature increased: MDC-700, 35.5 (1.58 mmol g^−1^, 196 K), 31.0 (1.38 mmol g^−1^, 273 K), and 26.1 m^3^ g^−1^ (1.17 mmol g^−1^, 298 K); MDC-800, 31.1 (1.39 mmol g^−1^, 196 K), 25.5 (1.14 mmol g^−1^, 273 K), and 21.9 m^3^ g^−1^ (0.98 mmol g^−1^, 298 K); MDC-900, 26.8 (1.19 mmol g^−1^, 196 K), 22.0 (0.98 mmol g^−1^, 273 K), and 19.9 m^3^ g^−1^ (0.89 mmol g^−1^, 298 K).

Based on N_2_ and CO_2_ sorption data, it became clear that the large physical dimension of the preorganized porphyrin backbone of microporous Zn-DpyDtolP-MOF resulted in a rather dense carbon material with a low porosity. Amounts of N-dopants of MDC-700, MDC-800, and MDC-900 were found to be 8.21, 6.77, and 6.41 wt% by elemental analysis, respectively. The temperature-dependence of the residual N-dopant content was clearly seen.^24^

### 3.4. Characterizations of MDCs and MDC-*n*KOHs

Powder X-ray diffraction (PXRD) patterns of all MDCs showed a typical diffraction pattern of disordered graphitic carbons partly due to dopants (Fig. S8, ESI†). Two very broad diffraction signals assignable to (002) and (100) planes were observed at around 24° and 44°, respectively.^24–27^ The presence of N-dopants was further confirmed by X-ray photoelectron spectroscopy (XPS) (Fig. S9 and S10, ESI†). Small amounts of residual Zn element were also observed for all MDCs (0.34–1.36 at%). However, there is no residual Zn element for KOH-activated samples. The amounts of residual Zn element are summarized in Table S6 (ESI†). Upon increase of carbonization temperature, the N-dopant contents gradually decreased. The types of N elements were also changed when we compared the high-resolution N1s XPS spectra of MDC-700, MDC-700-1KOH, and MDC-700-2KOH (Table S6 and Fig. S10, ESI†). The relatively abundant pyridinic-N (398.4 ± 0.1 eV) dopant for MDC-700 decreased while the contents of pyrrolic-N (399.7 ± 0.2 eV) dopant for both KOH-activated carbons increased.^28–30^ Both dopant types are known to contribute to pseudo-capacitance. Interestingly, the pyridone type of dopants (400.7 ± 0.2 eV) were not found in both KOH-activated samples. Additionally, the relative amounts of graphitic-N (also known as quaternary N) good for enhancing electrical conductivity dramatically increased for KOH-activated samples. Although the total amounts of N-dopants for MDC-700-*n*KOH samples are smaller than MDC-700, they are beneficial for enhancing capacitance in aqueous electrolyte.

The scanning electron microscopy (SEM) investigation for carbons samples indicates that the rod shape of pristine as-prepared Zn-DpyDtolP-MOF was conserved after carbonization (Fig. S11, ESI†). MDC-700-1KOH showed much smaller particles than MDC-700-2KOH.

Raman spectroscopic analysis of carbonaceous materials provides very useful information about the ordering of graphitic carbon layers.^26,27^ Thus, Raman spectra of the three MDCs were collected to investigate their graphitic nature by comparing ratios of *I*_D_/*I*_G_ from D- and G-bands (Fig. S12, ESI†). Typical Raman signals of graphitic and disordered carbon layers were observed around 1590 and 1343 cm^−1^, respectively.^26,27^ Interestingly, *I*_D_/*I*_G_ ratios increased in the order of MDC-700 (1.44) < MDC-800 (2.66) < MDC-900 (3.84). The lower the carbonization temperature, the higher the degree of graphitization. Furthermore, *I*_D_/*I*_G_ ratios of MDC-700 and MDC-800 were smaller than the known value of 3.03 for mesoporous CMK-3 carbonized at 900 °C for 20 h.^31^ Among these samples, MDC-700 exhibited the best graphitization. Thus, MDC-700 will be a better electrode material due to its better electrical conductivity.

### 3.5. Electrochemical performances of MDCs and MDC-*n*KOHs

**Fig. 3:**
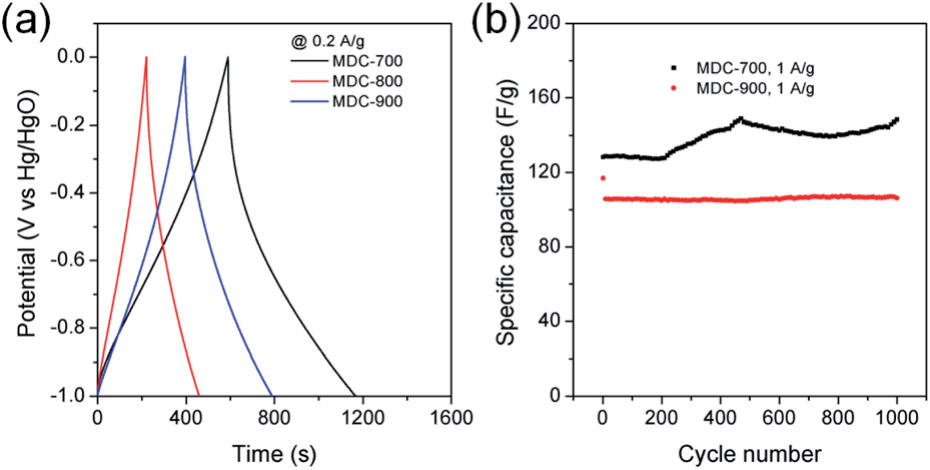
GCD results for the MDC-700, MDC-800 and MDC-900 under 3E system in 6 M KOH electrolyte (potential window: −1.0 V to 0.0 V *vs.* Hg/HgO). (a) GCD curves at 0.2 A g^−1^. (b) Variation of specific capacitance as a function of cycle number.

Initial assessment of electrochemical supercapacitive properties of the three MDC electrodes in electric double layer capacitance (EDLC)-based supercapacitors revealed that MDC-700 showed better performance than MDC-800 and MDC-900. Fig. 3a shows electrochemical properties of MDC carbon electrodes from the chronopotentiometry experiment in 6 M KOH electrolyte with a three-electrode (3E) cell configuration. The maximum specific capacitance at 0.2 A g^−1^ was 195.4 F g^−1^ for MDC-700, 71.5 F g^−1^ for MDC-800, and 125.5 F g^−1^ for MDC-900. The higher N-dopant content and the better microporosity of MDC-700 based on CO_2_ gas adsorption capacity might have synergistic effect in enhancing the maximum specific capacitance compared to the other two MDC electrodes. For MDC-700 and MDC-900, electrochemical recycling properties at 1 A g^−1^ were investigated up to 1000 cycles (Fig. 3b). The capacitance of the first cycle was 128.3 F g^−1^ for MDC-700 and 117.0 F g^−1^ for MDC-900. After 1000 cycles, both electrodes retained their specific capacitances: 148.4 F g^−1^ for MDC-700 (115.7%) and 106.2 F g^−1^ for MDC-900 (90.8%). Hence, the Zn-DpyDtolP-MOF is an ideal self-sacrificing carbonization precursor for N-doped porous carbon electrodes in supercapacitors. Electrochemical performances of carbon-based electrodes can be further improved by chemical activation.^24,32,33^ Thus, MDC-700 was chosen for activation with KOH. Two samples (*i.e.*, MDC-700-1KOH and MDC-700-2KOH) with different weight ratios of KOH to MDC-700 were then prepared.

**Fig. 4:**
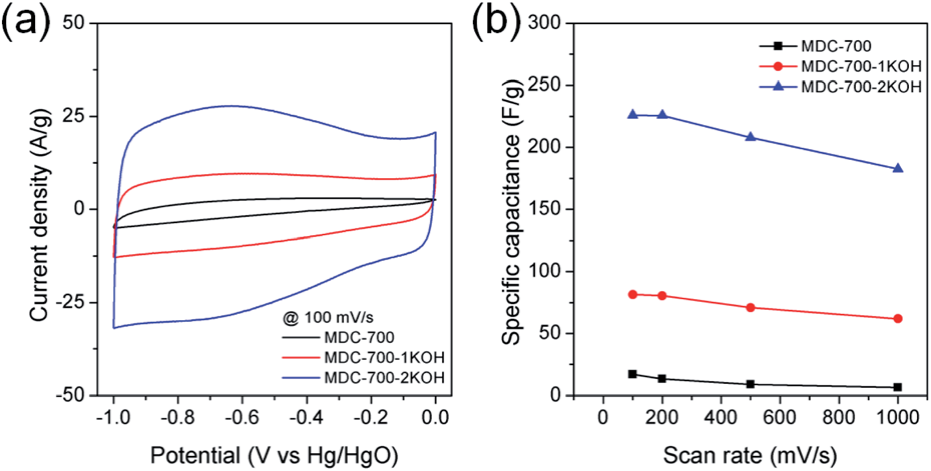
CV results for MDC-700, MDC-700-1KOH, and MDC-700-2KOH with a 3E system in 6 M KOH electrolyte (potential window: −1.0 V to 0.0 V *vs.* Hg/HgO). (a) CV curves at 100 mV s^−1^. (b) Dependence of specific capacitance on scan rate.

Cyclic voltammetry (CV), chronopotentiometry, and electrochemical impedance spectrometry (EIS) were used to determine electrochemical capacitive behaviors of bare MDC-700 and activated MDC-700-*n*KOH samples. To comparatively study the effect of KOH activation on capacitive capabilities of carbon samples, CV measurement was performed in 6 M KOH aqueous electrolyte with a 3E system (Fig. 4). In Fig. 4a, shapes of CV curves of MDC-700-*n*KOH samples reflected EDLC behavior combined with pseudo-capacitive character. The pseudo-capacitance can be clearly seen in a broad hump of CV curves. In our samples, the origin of the pseudo-capacitance might be the redox reaction from N- and O-dopants in carbon matrix.^34–40^ Contrarily, in the case of MDC-700, the shape of CV was significantly deformed from ideal rectangular geometry because the resistive character was coupled with the EDLC. This resistive character also affected the capacitive rate performance as shown in Fig. 4b. At a scan rate of 100 mV s^−1^, specific capacitances of MDC-700, MDC-700-1KOH and MDC-700-2KOH were 16.9, 81.5, and 225.9 F g^−1^, respectively. When the scan rate was increased to 1000 mV s^−1^, specific capacitances of MDC-700, MDC-700-1KOH and MDC-700-2KOH decreased to 6.3, 61.8, and 182.5 F g^−1^, respectively. When the scan rate was increased from 100 to 1000 mV s^−1^, capacitive retention rates of MDC-700, MDC-700-1KOH, and MDC-700-2KOH were 37.8, 75.8, and 80.8%, respectively. Thus, MDC-700-1KOH and MDC-700-2KOH exhibited good retention ability. CV curves of KOH-activated samples preserved the rectangular shapes even at a high scan rate of 1000 mV s^−1^ (Fig. S13a and S14a, ESI†). Due to microporosity of samples confirmed by gas sorption analysis, enhancements of specific capacitance and rate capability with KOH-activation could be seen. Widening the micropore dimension may reduce electrolyte transportation resistance by expanding electrochemically active surface area.

To investigate electrolytic effects on specific capacitances for MDC-700-*n*KOH samples in detail, CV curves were also obtained using neutral (1 M Na_2_SO_4_) and acidic (1 M H_2_SO_4_) electrolytes with the same 3E system and scan rate range (100–1000 mV s^−1^) of 6 M KOH (Fig. S13b, S13c, S14b, and S14c, ESI†). Both MDC-700-1KOH and MDC-700-2KOH samples had the lowest specific capacitances in 1 M Na_2_SO_4_ electrolyte. As previously observed, electrolyte conductivity dependence on specific capacitance may explain this result. The electrolyte conductivity increased in the order of KOH > H_2_SO_4_ > Na_2_SO_4_. The low conductivity of 1 M Na_2_SO_4_ electrolyte could hinder ionic transportation mediated electrosorption, thus decreasing the EDLC of carbon surface. When shapes of CV curves were compared, the contribution of pseudo-capacitance to overall specific capacitance was low in 1 M Na_2_SO_4_. Meanwhile, there existed two different trends of specific capacitance dependence on electrolyte between 1 M H_2_SO_4_ and 6 M KOH. In the case of MDC-700-2KOH, higher capacitances were measured in 6 M KOH than in 1 M H_2_SO_4_. In this case, different ion size between KOH and H_2_SO_4_ was an additional factor. The order of the size of ions is as follows:^41–43^OH^−^ < K^+^ ≈ H_3_O^+^ < SO_4_^2−^

Ion size difference between OH^−^ and SO_4_^2−^ might differently influence EDLC contribution to total specific capacitance. Small OH^−^ ion would have better electrostatic interaction and electrosorption on pores of carbon electrodes than SO_4_^2−^ ion. Besides, stronger pseudo-capacitive interactions between K^+^ and nitrogen functionalities than H_3_O^+^ also contribute to total capacitance.^41^

However, opposite trend of specific capacitances between 1 M H_2_SO_4_ and 6 M KOH was seen in MDC-700-1KOH. MDC-700-1KOH had lesser microporosity but more nitrogen content than MDC-700-2KOH. These distinct properties could explain the higher specific capacitance in 1 M H_2_SO_4_ than in 6 M KOH.^41,43^ Due to the basic surface of nitrogen functionalities, more N-doped MDC-700-1KOH might possess higher electronic charge density than MDC-700-2KOH. This basic surface character was likely to enhance ionic adsorption to improve the EDLC. In addition to EDLC, pseudo-capacitive contribution to overall specific capacitance could also be considered. In MDC-700-1KOH, stronger faradaic (pseudo-capacitive) interactions between nitrogen functional groups and H_3_O^+^ than those of K^+^ could have positive effects on the overall capacitance. Besides, faradaic reactions of nitrogen functional groups were favored by proton. Thus, the reactions provided additional pseudo-capacitance.

**Fig. 5:**
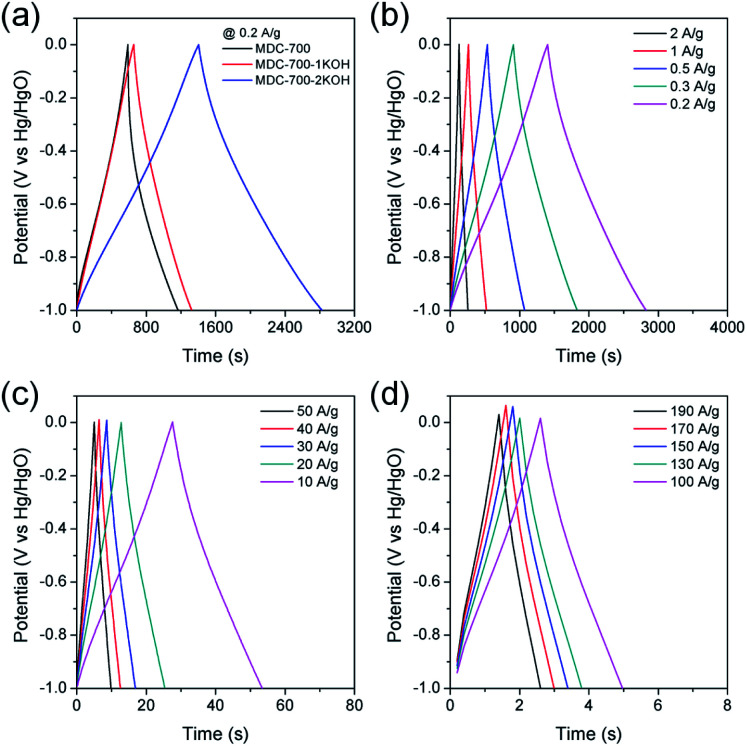
GCD curves in 6 M KOH electrolyte with a 3E system for MDC-700, MDC-700-1KOH, and MDC-700-2KOH at 0.2 A g^−1^ (a) and for MDC-700-2KOH at low (b), middle (c) and high current densities (d) (potential window: −1.0 V to 0.0 V *vs.* Hg/HgO).

To further study electrochemical performances of bare MDC-700 and KOH-activated samples, chronopotentiometry was conducted at various current loads in 6 M KOH. With the same electrolytic condition and a voltage range as those for the above CV measurements, galvanostatic charge–discharge (GCD) curves were obtained. Results are shown in Fig. 5. As can be seen in Fig. 5a, the discharge time of carbon samples increased with increasing degree of KOH activation. Thus, the EDLC induced from the discharge time had the same trend of order as the specific capacitance from the above CV curves. Moreover, charge–discharge curves of MDC-700-*n*KOH electrodes exhibited nearly symmetric and triangular shapes. These shapes of chronopotentiometry curves reflected the good EDLC character. For MDC-700-2KOH, the EDLC character retained at high current loads (Fig. 5b–d). Contrarily, the shape for the bare MDC-700 electrode was not highly symmetric. It deviated from an ideal triangle. This shape indicated resistive and pseudo-capacitive contributions to the overall capacitance.

**Fig. 6:**
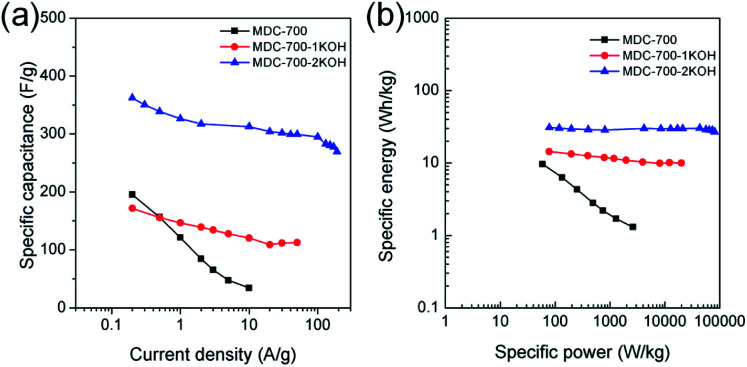
Electrochemical performances for MDC-700, MDC-700-1KOH, and MDC-700-2KOH from GCD curves with a 3E system in 6 M KOH electrolyte. (a) Dependence of specific capacitance on current density. (b) Ragone plots.

Electrochemical properties corresponding to chronopotentiometry curves are shown in Fig. 6 and Tables S7–S9 (ESI†). Fig. 6a reveals the dependence of total specific capacitance on current density. At 0.2 A g^−1^, total specific capacitances of MDC-700, MDC-700-1KOH, and MDC-700-2KOH were 195.4, 171.6, and 362.3 F g^−1^, respectively. MDC-700 exhibited poor rate capability with a low specific capacitance (34.3 F g^−1^). Its retention ratio was only 17.6% at 10 A g^−1^. On the other hand, MDC-700-*n*KOHs showed higher rate capabilities. For MDC-700-1KOH, the specific capacitance was 112.6 F g^−1^ at 50 A g^−1^ (65.6% retention). MDC-700-2KOH showed more improvement of rate capability. Even at 190 A g^−1^, its specific capacitance value was 269.3 F g^−1^ with a retention rate of 74.3%. Such different rate capabilities might be associated with equivalent series resistance (ESR). In the case of our samples, higher degree of KOH-activation induced lower ESR values with higher rate capability. The ESR reflects the overall internal resistance. It includes electrode's electronic resistance, ionic diffusional resistance, and the interface resistance between the current collector and the electrode.^44^ As summarized in Tables S7–S9 (ESI†), the MDC-700 exhibited higher resistance character than MDC-700-*n*KOH samples.

The total specific capacitance can be divided into two main parts: EDLC and pseudo-capacitance. Without considering resistive or pseudo-capacitive contribution to the overall specific capacitance, the EDLC component can be estimated from the ideal triangular shape of the charge–discharge curve. Therefore, EDLC could be estimated from the supposed ideal triangle without any significant deviations. The improved value trends of EDLC with respect to the degree of KOH-activation were coincident with those of specific capacitances from CV results (MDC-700 < MDC-700-1KOH < MDC-700-2KOH). More nitrogen-doped MDC-700 sample had a higher pseudo-capacitance ratio than KOH-activated MDC-700-*n*KOH samples (Tables S6–S9, ESI†). Thus, MDC-700 possessed higher specific capacitances at lower current densities (<1 A g^−1^), although it showed lower EDLC values than MDC-700-1KOH. At low current densities (<1 A g^−1^), the small gap of EDLC between MDC-700 and MDC-700-1KOH coupled with large gap of pseudo-capacitance contribution ratio between them might have generated higher capacitances for MDC-700.

Electrolytic effects of electrochemical performances for MDC-700-*n*KOHs were also studied using chronopotentiometry (Fig. S15–S18, Tables S8–S13, ESI†). GCD curves were obtained with the same electrode configuration and voltage range for the above CV. For MDC-700-1KOH, the dependence of specific capacitance on electrolytes (Fig. S17a, ESI†) has the same trend as those for previous CV curves (1 M H_2_SO_4_ > 6 M KOH > 1 M Na_2_SO_4_). At 0.2 A g^−1^, total specific capacitances in 6 M KOH, 1 M Na_2_SO_4_, and 1 M H_2_SO_4_ were 171.6, 94.7, and 589.7 F g^−1^, respectively. In 1 M H_2_SO_4_, the specific capacitance was as high as 145.7 F g^−1^ at up to 100 A g^−1^ with a retention ratio of 24.7%. However, in other electrolytes, low specific capacitances were observed at high current density. The capacitance was 30.2 F g^−1^ at 20 A g^−1^ (31.9% retention) in 1 M Na_2_SO_4_. It was 112.6 F g^−1^ at 50 A g^−1^ in 6 M KOH (65.6% retention).

Effects of electrolytes on electrochemical performances were also investigated for MDC-700-2KOH. Fig. S17b (ESI†) shows specific capacitance dependence on electrolyte in MDC-700-2KOH. At 0.2 A g^−1^, maximum values of total specific capacitance in 6 M KOH, 1 M Na_2_SO_4_, and 1 M H_2_SO_4_ were 362.3, 197.2, and 555.6 F g^−1^, respectively. Minimum total specific capacitance values of MDC-700-2KOH in 6 M KOH, 1 M Na_2_SO_4_, and 1 M H_2_SO_4_ at 190, 120, and 150 A g^−1^ were 269.3, 148.7, and 201.9 F g^−1^, respectively (corresponding retention rates: 74.3, 75.4, and 36.3%, respectively). Compared to other electrolytes, low EDLC of 1 M Na_2_SO_4_ might be resulted from different electrolyte conductivity (6 M KOH > 1 M H_2_SO_4_ > 1 M Na_2_SO_4_). In addition, there were two different trends at intersection point of about 1 A g^−1^. At current densities of 1 A g^−1^ or above, the same trend of specific capacitance dependence on electrolyte as described in CV earlier was seen (6 M KOH > 1 M H_2_SO_4_ > 1 M Na_2_SO_4_). However, there was a reverse trend of such dependence on electrolytes for 6 M KOH and 1 M H_2_SO_4_ in the range 0.5–0.2 A g^−1^. These different trends could also be attributed to synergistic contribution of EDLC and pseudo-capacitance. EDLC from different electrolyte conductivity between 6 M KOH and 1 M H_2_SO_4_ could be applied down to 1 A g^−1^ (6 M KOH > 1 M H_2_SO_4_). Conversely, at lower current densities than 1 A g^−1^, the basic surface of nitrogen functionalities could drive enhanced ionic adsorption to improve the EDLC of 1 M H_2_SO_4_ compared to 6 M KOH. Besides, 1 M H_2_SO_4_ showed higher pseudo-capacitive contribution ratio than other electrolytes. Not only faradaic interactions between nitrogen functionalities and electrolyte's cation, but also faradaic reactions of nitrogen functional groups with proton could affect this contribution.

Ragone plots of bare MDC-700 and KOH-activated samples are depicted in Fig. 6b. At a current density of 0.2 A g^−1^, the maximum specific energy was 9.6 W h kg^−1^ for MDC-700, 14.4 W h kg^−1^ for MDC-700-1KOH, and 30.8 W h kg^−1^ for MDC-700-2KOH. At 10 A g^−1^, the MDC-700 had a minimum specific energy of 1.3 W h kg^−1^ with a low maximum specific power of 2625 W kg^−1^. Contrarily, MDC-700-1KOH retained a specific energy of 10.0 W h kg^−1^ at 50 A g^−1^ with a maximum specific power of 19 980 W kg^−1^. More improved retention of specific energy (26.8 W h kg^−1^) and maximum specific power (80 423 W kg^−1^) were observed for MDC-700-2KOH even at 190 A g^−1^. As mentioned earlier, microporosity development by KOH-activation could lead to enhancement of the electrochemically active surface area with a decrease of ESR. As a result, the rate capability of MDC-700-2KOH was superior to other carbon samples in our experiments. It is important to study the retention of capacitance and energy of supercapacitors at high operational conditions.^45^ The observed specific energy and power with high rate capability for MDC-700-2KOH were comparable to other good supercapacitors (Table S14, ESI†). For both MDC-700-1KOH and MDC-700-2KOH, electrolyte effects on the Ragone plot are also plotted in Fig. S18 (ESI†). MDC-700-1KOH had a poorer performance in 1 M Na_2_SO_4_ than in 6 M KOH. However, it had better performance in 1 M H_2_SO_4_ than in 6 M KOH. The maximum specific energy at 0.2 A g^−1^ was 14.4 W h kg^−1^ for 6 M KOH, 7.7 W h kg^−1^ for 1 M Na_2_SO_4_, and 26.0 W h kg^−1^ for 1 M H_2_SO_4_. At 20 A g^−1^, the 1 M Na_2_SO_4_ showed a minimum specific energy of 2.7 W h kg^−1^ with a low maximum specific power of 7973 W kg^−1^. On the contrary, the specific energy of 1 M H_2_SO_4_ had a moderately high value of 15.4 W h kg^−1^ at 100 A g^−1^ with a high specific power of 43 885 W kg^−1^. MDC-700-2KOH exhibited superior performances to MDC-700-1KOH in all electrolytes. The maximum specific energy at 0.2 A g^−1^ was 30.8 W h kg^−1^ in 6 M KOH, 22.3 W h kg^−1^ in 1 M Na_2_SO_4_, and 40.4 W h kg^−1^ in 1 M H_2_SO_4_. In 1 M Na_2_SO_4_, its specific energy was 13.5 W h kg^−1^ at 120 A g^−1^ with the highest specific power of 48 420 W kg^−1^. At 150 A g^−1^, enhanced retention of specific energy of 15.5 W h kg^−1^ and maximum specific power of 55 735 W kg^−1^ in 1 M H_2_SO_4_ were found. Table S15 (ESI†) tabulates electrochemical properties related to Ragone plot in H_2_SO_4_ electrolyte to compare the current systems with other reported values. It demonstrated that both MDC-700-1KOH and MDC-700-2KOH also showed good performances in H_2_SO_4_ electrolyte.

**Fig. 7:**
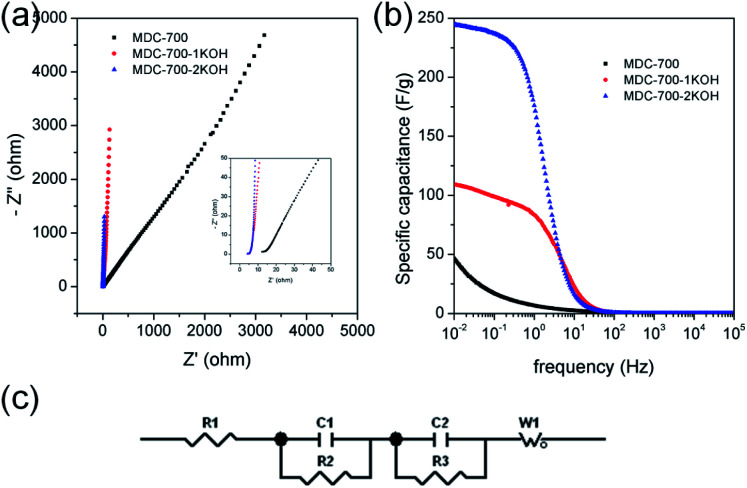
EIS results for MDC-700, MDC-700-1KOH, and MDC-700-2KOH with a 3E system in 6 M KOH electrolyte (applied constant potential: −0.5 V *vs.* Hg/HgO). (a) Nyquist plots. (b) Bode plots from specific capacitance. (c) Equivalent circuit.

EIS can be used to rigorously analyze capacitive and resistive behaviors of carbon electrodes. With frequencies from 10^5^ to 10^−2^ Hz, impedances were measured at a constant potential with a 10 mV potential amplitude. Fig. 7a shows Nyquist plots of AC impedance spectra for MDC-700, MDC-700-1KOH, and MDC-700-2KOH in 6 M KOH solution. From the *x*-intercept at high frequency region, the internal resistance of ESR was estimated. The ESR includes electrolyte resistance, intrinsic resistance of carbon electrode, and the contact resistance at the carbon material/current collector interface.^34,41,44^ Both MDC-700-1KOH and MDC-700-2KOH had lower ESR than MDC-700. This trend was in accordance with ESR obtained from chronopotentiometry curves (Tables S7–S9, ESI†). Negligibly small semicircles were visible in the inset of Fig. 7a. This phenomenon could be ascribed to a low pseudo-capacitive interaction,^41^ a low interface resistance between electrode and electrolyte,^46^ a high ionic conductivity,^47,48^ and a high electronic conductivity.^47^ Generally, there is a diffusive resistance (Warburg impedance) line with 45° slope in the middle frequency region.^44,49–51^ Comparing middle and low frequency regions in the Nyquist plots, it was found that MDC-700-*n*KOHs also had lower ionic diffusion resistance than bare MDC-700. Thus, diffusion was not a major control factor in electronic kinetics of MDC-700-1KOH or MDC-700-2KOH. It has been reported that the vertical line of 90° slope of MDC-700-*n*KOHs in the Nyquist plot indicates an ideal capacitive behavior with a low ionic resistance.^48^ Thus, MDC-700-1KOH and MDC-700-2KOH exhibited more capacitive character but less ionic diffusional resistance in middle and low frequency regions than MDC-700.

Frequency-dependent specific capacitance plots (Fig. 7b) also reflected kinetic features of ionic diffusion. At 0.01 Hz, the maximum value of specific capacitance was 46.6 F g^−1^ for MDC-700, 108.7 F g^−1^ for MDC-700-1KOH, and 244.4 F g^−1^ for MDC-700-2KOH. These values were similar to those from GCD curves at the highest current densities. They were mostly inversely proportional to absolute values of imaginary impedances at 0.01 Hz. At a frequency of 1 Hz, the retention ratio (%) of the specific capacitance to the highest value was 14.3% for MDC-700, 76.8% for MDC-700-1KOH, and 72.1% for MDC-700-2KOH. Thus, MDC-700-*n*KOHs had better frequency responses than non-activated MDC-700. These better behaviors were also supported by operating frequency. The operating frequency (*f*_0_) is a point of frequency at which capacitive retention ratio is 50%.^49,52,53^ Calculated operating frequency values for MDC-700, MDC-700-1KOH, and MDC-700-2KOH were 0.04948, 3.831, and 1.896 Hz, respectively. According to the relationship of *τ*_0_ = 1/*f*_0_, the relaxation time constant (*τ*_0_) was also estimated. The estimated relaxation time constant was 20.2102 s for MDC-700, 0.2610 s for MDC-700-1KOH, and 0.5274 s for MDC-700-2KOH. In brief, MDC-700-1KOH and MDC-700-2KOH had higher operation frequency but lower relaxation time constants than MDC-700. This demonstrated that MDC-700-*n*KOHs possessed better kinetic features about ionic diffusion and higher rate capabilities than MDC-700. Using the reported equivalent circuit (Fig. 7c),^54^ best fitting values were obtained and tabulated in Table S16 (ESI†). Superior characters of MDC-700-1KOH and MDC-700-2KOH were also supported by these fitting values.

EIS analyses in other electrolytes were also conducted to investigate electrolytic effects on electrochemical performances (Fig. S19 and S20 and Tables S17 and S18, ESI†). With the exception of the applied constant potential (−0.5 V *vs.* Ag/AgCl for 1 M Na_2_SO_4_, 0.25 V *vs.* Ag/AgCl for 1 M H_2_SO_4_), impedances were measured with the same method for 6 M KOH. Besides, the same equivalent circuit used in the basic electrolyte earlier was also applied to fit measured data for neutral and acidic electrolytes. Results of EIS revealed that basic and acidic electrolytes had better electrochemical performances than neutral electrolyte.

**Fig. 8:**
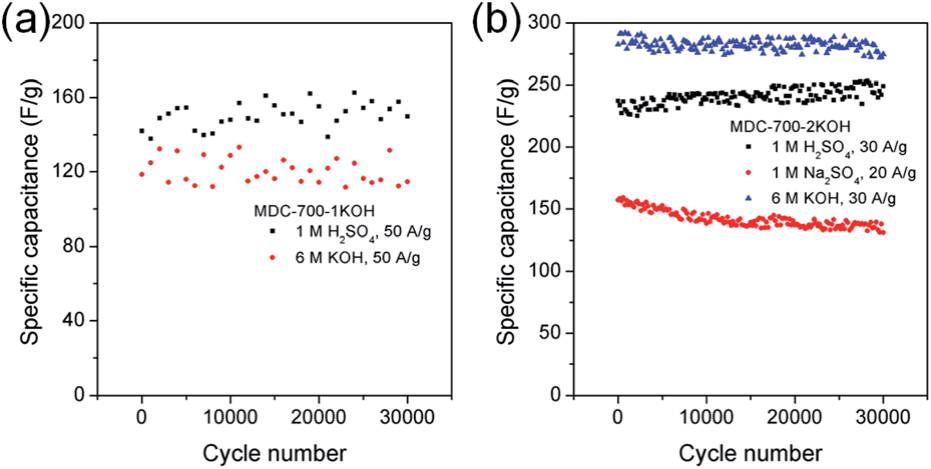
Variation of specific capacitance as a function of cycle number from GCD curves in 3E system for (a) MDC-700-1KOH and (b) MDC-700-2KOH.

Long cycling stability of an electrode material is a critical factor for high performance supercapacitors. Thus, electrochemical cycling properties of MDC-700-1KOH and MDC-700-2KOH were investigated up to 30 000 cycles using GCD measurements (Fig. 8). Cycling stabilities were also investigated by EIS (Fig. S21 and S22, ESI†). The specific capacitance of the first cycle of MDC-700-1KOH electrode at 50 A g^−1^ was 118.7 F g^−1^ in 6 M KOH and 142.0 F g^−1^ in 1 M H_2_SO_4_. After 30 000 cycles, its specific capacitances in 6 M KOH and 1 M H_2_SO_4_ were 114.6 F g^−1^ (96.5% retention) and 149.9 F g^−1^ (105.6% retention), respectively. GCD cycling of MDC-700-2KOH was also performed under more stable conditions than that of MDC-700-1KOH (<50 A g^−1^). At the first cycle of 30 A g^−1^, the specific capacitance of MDC-700-2KOH in 1 M Na_2_SO_4_ was 157.3 F g^−1^. The specific capacitance decreased slightly to 131.0 F g^−1^ (16.7% loss) after 30 000 cycles. Compared to the electrolytic condition of 1 M Na_2_SO_4_, better cycling performances could be seen in 6 M KOH and 1 M H_2_SO_4_. The specific capacitances started with 282.5 (6 M KOH) and 237.3 F g^−1^ (1 M H_2_SO_4_) at the very beginning cycle of 20 A g^−1^. At the 30000th cycle, the specific capacitance was 274.7 F g^−1^ (97.2% retention) in 6 M KOH and 249.1 F g^−1^ (105.0% retention) in 1 M H_2_SO_4_. For comparison, published data of electrochemical cycling properties of other carbon electrodes are summarized in Tables S19 and S20 (ESI†) for 6 M KOH and 1 M H_2_SO_4_, respectively. Based on the data in these tables, MDC-700-1KOH and MDC-700-2KOH electrodes showed good cycling performances at higher operating conditions than other known carbons.

**Fig. 9:**
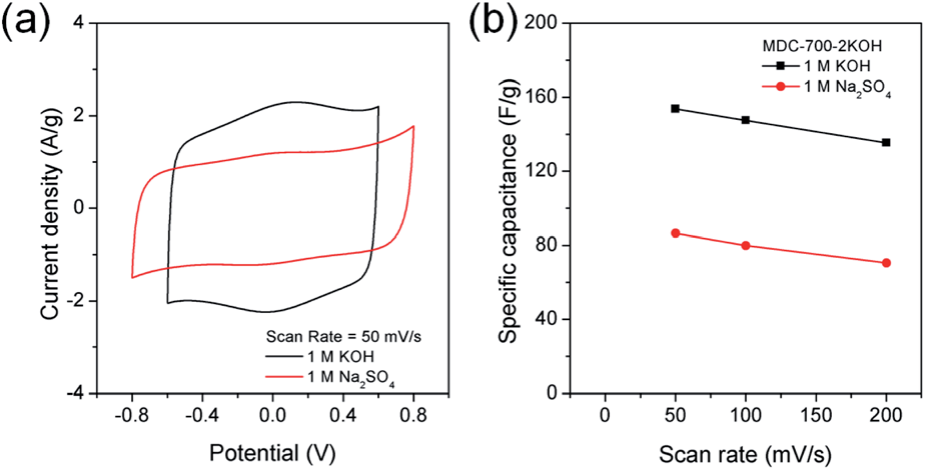
CV analysis of MDC-700-2KOH using Swagelok cell-based 2E system in different electrolytes (potential windows in 1 M KOH: −0.6 V to 0.6 V, in 1 M Na_2_SO_4_: −0.8 V to 0.8 V). (a) CV curves at 50 mV s^−1^. (b) Dependence of specific capacitance on scan rate.

Swagelok type of cell was assembled to investigate electrochemical properties of MDC-700-2KOH in a symmetric 2E system. These properties were gained from basic (1 M KOH) and neutral (1 M Na_2_SO_4_) electrolytic conditions. Like the 3E system, CV measurements were also performed (Fig. 9). As displayed in Fig. 9a, 1 M KOH electrolytic condition exhibited pseudocapacitive behavior more clearly than 1 M Na_2_SO_4_ electrolytic condition. This pseudocapacitive behavior, revealed by a pair of humps, could be generated from functionalities of nitrogen and oxygen. The specific capacitance dependence on scan rate was plotted in Fig. 9b.

Maximal specific capacitances of MDC-700-2KOH obtained at 50 mV s^−1^ were 153.8 and 86.4 F g^−1^ in 1 M KOH and 1 M Na_2_SO_4_, respectively. Minimal specific capacitances obtained at 200 mV s^−1^ were 135.4 (88.0% retention) and 70.6 F g^−1^ (81.7% retention) in 1 M KOH and 1 M Na_2_SO_4_, respectively. Therefore, 1 M KOH showed superior capacitive properties than 1 M Na_2_SO_4_ as described previously in a 3E system.

**Fig. 10:**
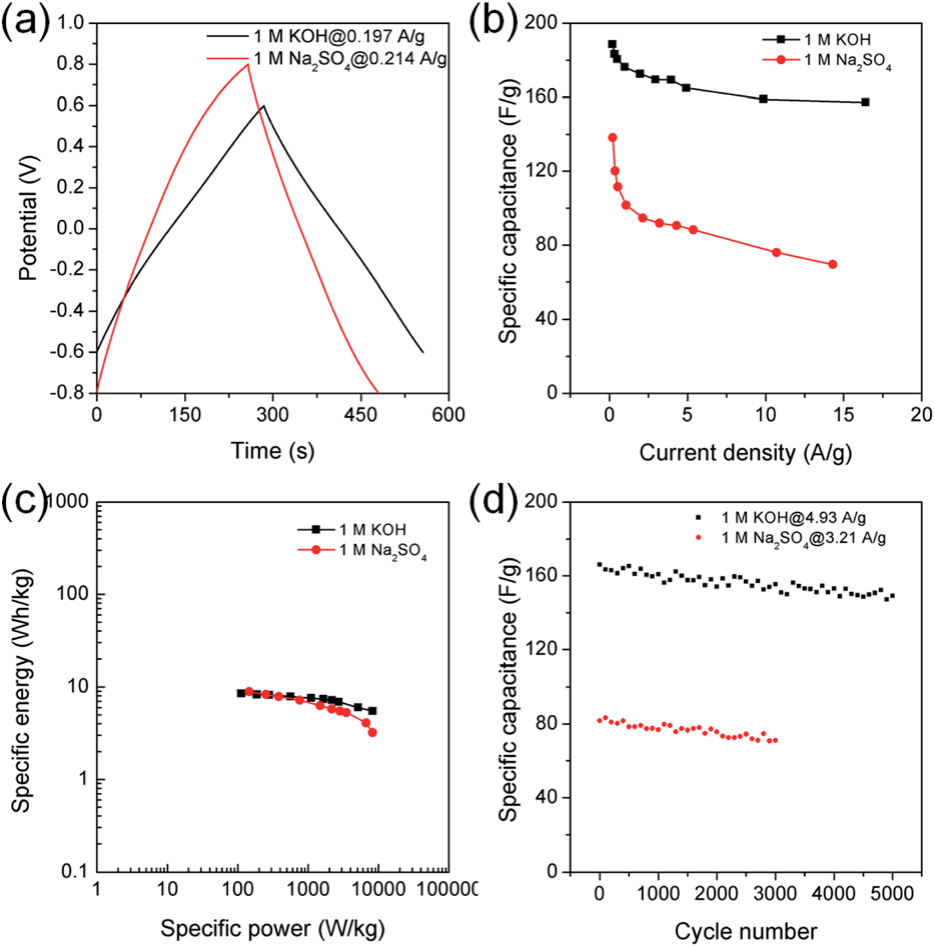
GCD results for MDC-700-2KOH under Swagelok type cell-based 2E system in different electrolytes (potential windows at 1 M KOH: −0.6 V to 0.6 V, at 1 M Na_2_SO_4_: −0.8 V to 0.8 V). (a) GCD curves at the lowest current densities. (b) Dependence of specific capacitance on current density. (c) Ragone plots. (d) Variation of specific capacitance as a function of cycle number.

**Fig. 11:**
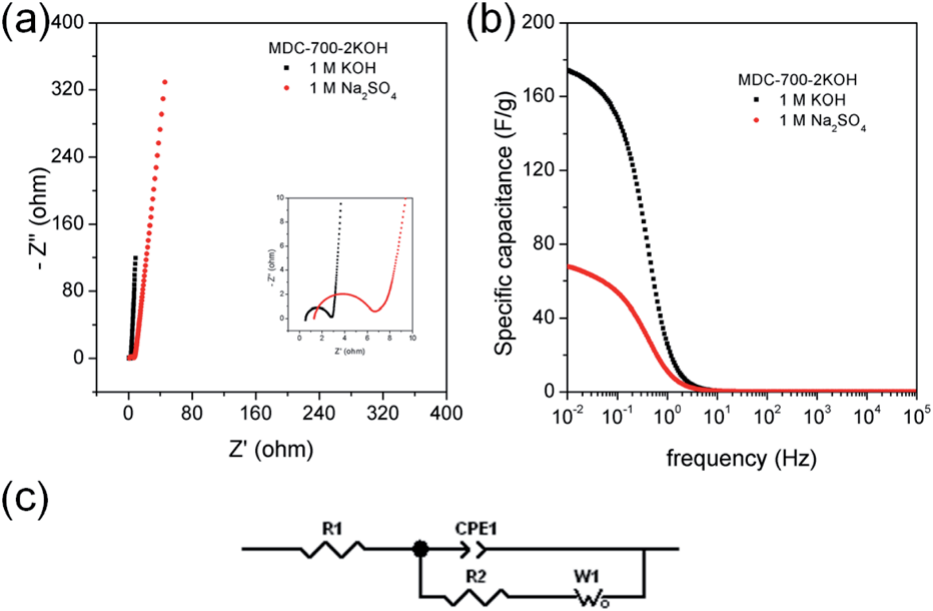
EIS results for MDC-700-2KOH in different electrolytes under Swagelok cell-based 2E system (applied constant potential: 0 V). (a) Nyquist plots. (b) Bode plots from specific capacitance. (c) Equivalent circuit.

Chronopotentiometry was also performed to further study electrochemical properties of MDC-700-2KOH electrode in a Swagelok cell-based 2E system (Fig. 10, S23, S24, and Tables S21, S22, ESI†). Fig. 10b shows specific capacitance dependence on current density. The maximum specific capacitance was 188.5 F g^−1^ in 1 M KOH at 0.197 A g^−1^ and 138.2 F g^−1^ in 1 M Na_2_SO_4_ at 0.214 A g^−1^. In a basic electrolyte, the specific capacitance slightly decreased to 157.3 F g^−1^ at 16.4 A g^−1^ (83.4% retention). In a neutral electrolyte, the value was 69.5 F g^−1^ at 14.3 A g^−1^ (50.3% retention). In 1 M KOH at 0.197 A g^−1^, the highest specific energy was 8.5 W h kg^−1^ and the lowest specific power was 113 W kg^−1^ (Fig. 10c). At an increased current density of 16.4 A g^−1^, the lowest specific energy was 5.5 W h kg^−1^ and the highest specific power was 8256 W kg^−1^. Table S23 (ESI†) summarizes reported performance data for other carbon electrodes in KOH electrolyte. Compared to these literature data, performances of MDC-700-2KOH electrode operating in a relatively low concentration of electrolyte (1 M) were quite comparable to those of other reported samples. Besides, the highest specific energy was 8.9 W h kg^−1^ and the lowest specific power was 146 W kg^−1^ in 1 M Na_2_SO_4_ at 0.214 A g^−1^. At 14.3 A g^−1^, the lowest specific energy was 3.2 W h kg^−1^ and the highest specific power was 8216 W kg^−1^. Cyclic performances were investigated up to 3000 cycles in 1 M Na_2_SO_4_ and 5000 cycles in 1 M KOH (Fig. 10d). At the first cycle, the specific capacitance was 166.1 F g^−1^ in 1 M KOH at 4.93 A g^−1^ and 81.8 F g^−1^ in 1 M Na_2_SO_4_ at 3.21 A g^−1^. Its specific capacitances in 1 M KOH at 4.93 A g^−1^ and in 1 M Na_2_SO_4_ at 3.21 A g^−1^ were 149.2 (89.8% retention) and 71.7 F g^−1^ (86.9% retention), respectively. As summarized in Table S24 (ESI†), MDC-700-2KOH showed better cycling performances in basic electrolyte than other carbons. In addition, higher resistive features in a neutral electrolyte were confirmed as summarized in Tables S21 and S22 (ESI†). This was additionally confirmed by EIS analysis (Fig. 11 and Table S25, ESI†). Thus, a basic electrolytic condition was electrochemically superior to a neutral electrolytic condition. EIS analysis was also performed with the applied constant potential of 0 V in the Swagelok cell-based 2E system (Fig. 11, Table S25 and Fig. S25, ESI†). The basic electrolytic condition yielded higher maximum specific capacitance of 174.3 F g^−1^ at 0.01 Hz, higher operating frequency of 0.3831 Hz, and shorter relaxation time constant (2.610 s) compared to the neutral electrolytic condition (67.8 F g^−1^, 0.3371 Hz, and 2.966 s). The basic electrolytic condition also showed better performances after fitting the equivalent circuit (Fig. 11c) than the neutral electrolytic condition (Table S25, ESI†).^44^ Therefore, the basic electrolytic condition is more favorable for better performances in the Swagelok cell-based 2E system like in the 3E system. The 2E cell with 1 M Na_2_SO_4_ electrolyte successfully enlightened a light-emitting diode (LED) with a minimum operating voltage of 1.2 V (Fig. S26, ESI†).

Other symmetric 2E cells were assembled for an acidic electrolytic condition (2 M H_2_SO_4_) with a relatively narrow potential window (−0.5 to 0.5 V). Two different acid-resistant current collectors such as Ti plate and ITO glass were used to support 2E cells. From CV curves shown in Fig. S27 (ESI†), the Ti plate-based system exhibited better performances (117.0 F g^−1^ at 50 mV s^−1^ and 80.0 F g^−1^ at 200 mV s^−1^) than the ITO glass-based system (75.6 F g^−1^ at 50 mV s^−1^ and 19.4 F g^−1^ at 200 mV s^−1^). Furthermore, the Ti plate-based system showed mostly superior properties from GCD curves (Fig. S28 and Tables S26 and S27, ESI†) to the ITO glass-based system with the exception of maximum specific capacitance (379.8 F g^−1^ at 0.2 A g^−1^) and cycling stability (101.0% capacitive retention after 10 000 cycles at 2 A g^−1^). At 0.2 A g^−1^, the Ti plate-based system delivered slightly lower capacitance of 369.2 F g^−1^, specific energy of 6.3 W h kg^−1^, and specific power of 70 W kg^−1^. In addition, the Ti plate-based system delivered a capacitance of 115.2 F g^−1^, a specific energy of 2.1 W h kg^−1^, and a specific power of 5468 W kg^−1^ at 15 A g^−1^. These data from the Ragone plot were comparable to those of other reported carbon samples derived from MOF-based precursors under the same potential window range (Table S28, ESI†). At 3 A g^−1^, the Ti plate-based system exhibited capacitive retention of 78.5% after 10 000 cycles (from 172.1 to 135.1 F g^−1^). Cycling stabilities of other carbon electrodes with the same potential window (1.0 V) and cycle number (10 000 cycles) are tabulated (Table S29, ESI†).

EIS analysis was performed for an acidic electrolytic condition with the applied potential of 0 V in 2E systems supported by Ti plate and ITO glass (Fig. S29 and Table S30, ESI†). The Ti plate-based system held a higher maximal specific capacitance of 134.7 F g^−1^ at 0.01 Hz, a higher operating frequency of 0.2448 Hz, and a lower relaxation time constant of 4.085 s than the ITO glass-based system (116.1 F g^−1^, 0.04948 Hz, and 210 s, respectively). In addition, the Ti plate-based system showed superior results to the ITO glass-based system (Table S30, ESI†) after fitting equivalent circuit^44^ (Fig. S29c, ESI†) using a slightly different from the Swagelok cell based 2E system. Consequently, the Ti plate-based system yielded better performances than the ITO glass-based system.

## 4. Conclusions

A new porphyrin-based Zn-DpyDtolP-MOF (ZnDpyDtolP·1/2DMF, H_2_DpyDtolP = 5,15-di(4-pyridyl)-10,20-di(4-methylphenyl)porphyrin) with a 3D hexagonal network as a self-sacrificing template for N-doped porous carbons through direct carbonization was prepared and structurally characterized. Despite its relatively low surface area, the solvent-free Zn-DpyDtolP-MOF exhibited a good microporosity for capturing CO_2_ at low temperature. N-doped porous MOF-derived carbons (MDCs) were directly synthesized from Zn-DpyDtolP-MOF through a self-templated single-step carbonization. KOH-activated MDC derivatives, namely MDC-700-*n*KOH, were also prepared by using different weight ratios of KOH activator to MDC (MDC : KOH = 1 : *n*, where *n* = 1 or 2). MDC-700-1KOH and MDC-700-2KOH exhibited much improved gas sorption and electrochemical capacitive properties due to their more profound microporosity and surface areas than MDCs. These activated carbon samples also exhibited much enhanced high rate operating capacitive performances. The maximum values of the gravimetric specific capacitance and specific energy for N-doped MDC-700-2KOH were higher than those of other known porous carbons. It also exhibited a high-rate capacitive performance with a large specific power. MDC-700-*n*KOH electrodes also showed very good recycling properties of electrochemical capacitance up to 30 000 cycles. Through simple carbonization of porphyrin-based Zn-MOF followed by a suitable activation, very competing porous carbons with N-dopants and more developed microporosity were obtained. We envision that a range of new porphyrin-MOF-based self-sacrificing templates would be possible for high performance charge storage electrode materials.

## Conflicts of interest

There are no conflicts to declare.

## Supplementary Material

RA-012-D2RA00327A-s001

RA-012-D2RA00327A-s002
