# Supplementary material for: N-doped porous carbons derived from Zn-porphyrin-MOF
Source: RSC Adv. 2022 Feb 18;12(10):5979–89. doi: 10.1039/d2ra00327a (PMC8981994; doi:10.1039/d2ra00327a)
Supplement: RA-012-D2RA00327A-s001 [file RA-012-D2RA00327A-s001.pdf]

Electronic Supplementary Material (ESI) for RSC Advances.
This journal is © The Royal Society of Chemistry 2022

Supporting Information

N-doped porous carbons derived from Zn-porphyrin-MOF

Hyun-Chul Kim,[a] Jongho Yoon,[b] Sukbin Yoon,[a] Youngmee Kim,*[c] Suk Joong Lee*[b] and Seong Huh*[a]

[a]*Department of Chemistry and Protein Research Center for Bio-Industry, Hankuk University of Foreign Studies, Yongin 17035, Republic of Korea. E-mail: shuh@hufs.ac.kr*

[b]*Department of Chemistry, Research Institute for Natural Science, Korea University, Seoul 136-701, Republic of Korea, E-mail: slee1@korea.ac.kr*

[c]*Department of Chemistry and Nano Science, Ewha Womans University, Seoul 120-750, Republic of Korea, E-mail: ymeekim@ewha.ac.kr*

Calculation of specific capacitance, specific energy, and specific power

1. Three-electrode (3E) system

(a) Cyclic voltammetry (CV)

The specific capacitance can be calculated from the following equation [1].

$$C_S = \frac{I_a + |I_c|}{2\,m\,(dv/dt)}$$

C_S: specific capacitance (F g^{-1})

I_a: anodic voltammetric current (A)

I_a: cathodic voltammetric current (A)

2: average factor

m: mass of the active material (g)

dv/dt: potential scan rate (V s^{-1})

(b) Chronopotentiometry (Galvanostatic charge/discharge, GCD)

The specific capacitance can be estimated by the following equation [2].

$$C_S = \frac{2 \times I}{m \times (\Delta V)^2} \int V(t)dt$$

I: discharging current (A)

V: potential (V)

m: mass of the active material (g)

ΔV: actual potential window during the discharging (V).

The value of ΔV can be obtained from the following equation [3].

$$\Delta V = V_{appl} - IR = V_{appl} - I \times ESR$$

V_{appl}: applied potential window during the discharging (V)

IR: potential drop (V)

ESR: equivalent series resistance (Ω)

Assuming that the shape of the GCD curve is ideally triangular (*ESR* = 0, $\int V(t)dt = \Delta V \times \Delta t/2$), the specific capacitance can be replaced by *EDLC* [2a,2c].

$$EDLC = \frac{I \times \Delta t}{m \times V_{appl}}$$

Δt: discharging time (s)

EDLC: electric double layer capacitance (F g^{-1})

The specific energy (*E*) and specific power (*P*) can be evaluated by using the given equations [2b,4].

$$E = \frac{1000 \times C_S \times (\Delta V)_2}{3600 \times 2}$$

$$P = \frac{3600 \times E}{\Delta t}$$

E: specific energy (Wh kg^{-1})

P: specific power (W kg^{-1})

(c) Electrochemical impedance spectroscopy (EIS)

Using the following equation, frequency dependent specific capacitance can be derived from impedance values [2a,5].

$$C_S = \frac{-Z''}{2\pi f|Z|^2 \times m} = \frac{|Z''|}{2\pi f|(Z')^2 + (Z'')^2| \times m}$$

f: applied frequency (Hz)

Z: total impedance

Z′: real impedance

Z″: imaginary impedance

2. 2E system

The specific capacitance of single electrode (C_S) can be acquired from the specific capacitance of 2E supercapacitor cell (C_T) [6].

$$C_S = 4 \times C_T$$

By substituting *m* with *M* (total mass of the active materials), the value of C_T can be gained from the above equations described in 3E system. Thus, the specific energy (*E*, Wh kg^{-1}) and specific power (*P*, W kg^{-1}) for 2E cell can also be applied to the following equations in the same manner.

$$E = \frac{1000 \times C_T \times (\Delta V)^2}{3600 \times 2} = \frac{1000 \times C_S \times (\Delta V)^2}{3600 \times 8}$$

$$P = \frac{3600 \times E}{\Delta t}$$

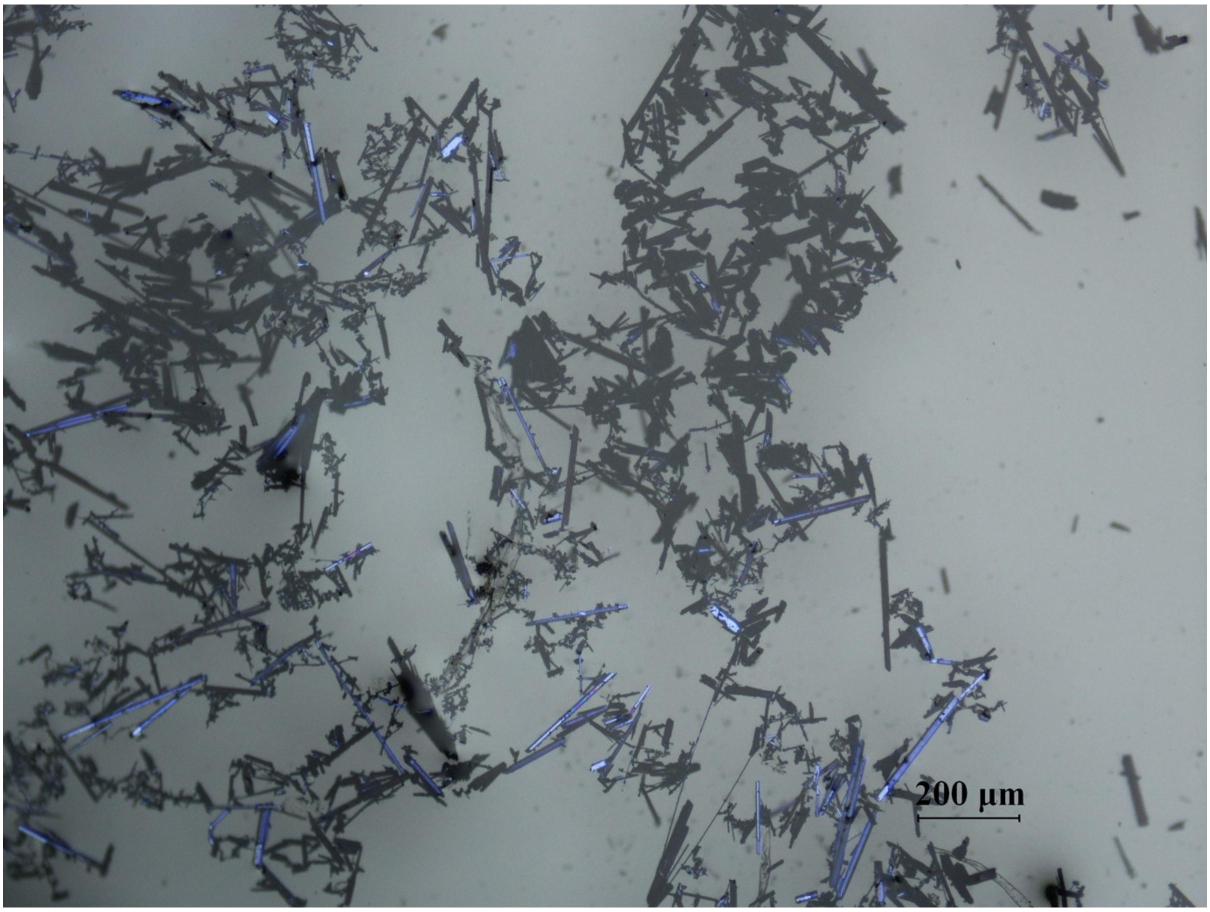

Figure S1. Optical microscopic image of the as-prepared Zn-DpyDtolP-MOF.

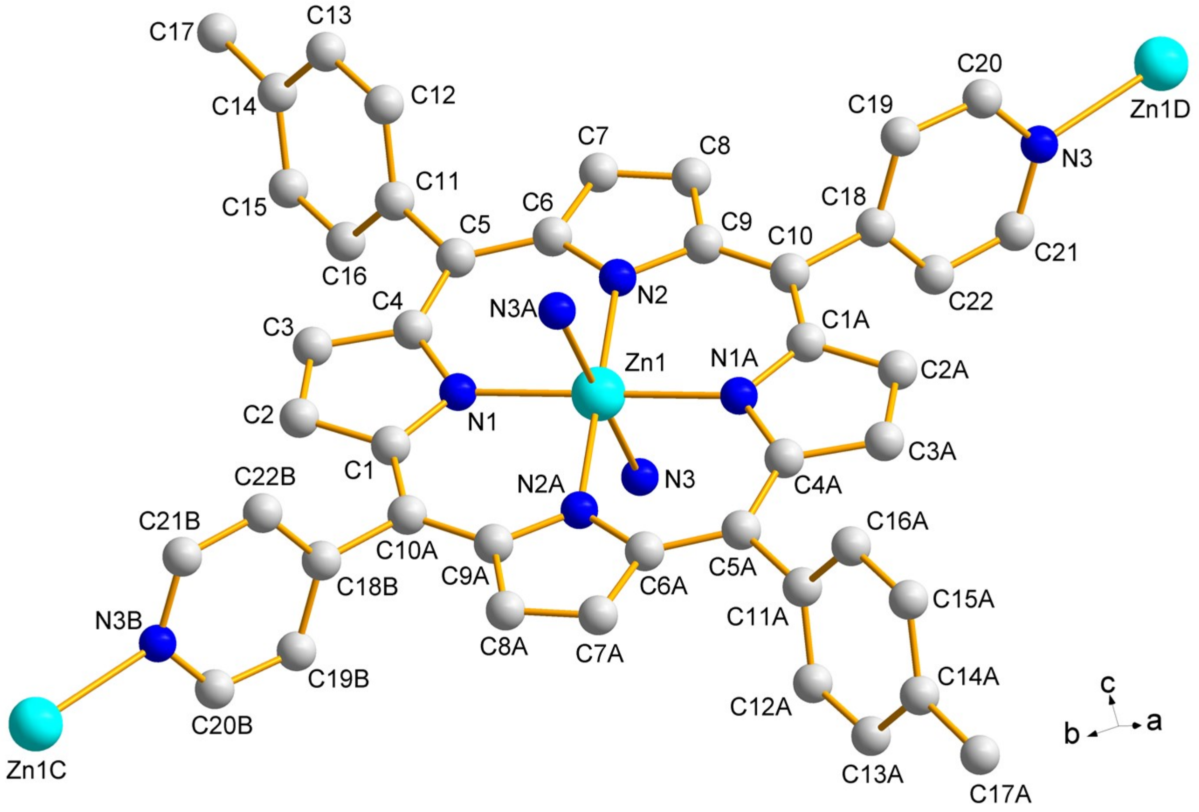

Figure S2. The atom numbering scheme of the asymmetric unit of Zn-DpyDtolP-MOF. All hydrogen atoms are removed for clarity. The DMF hemisolvate could not be refined due to disordering. Symmetry codes: A $1 - x$, $-y$, $-z$; B $1/3 + y$, $2/3 - x + y$, $-1/3 - z$; C $1/3 - y$, $-1/3 + x - y$, $-1/3 + z$; D $2/3 - y$, $-2/3 + x - y$, $1/3 + z$.

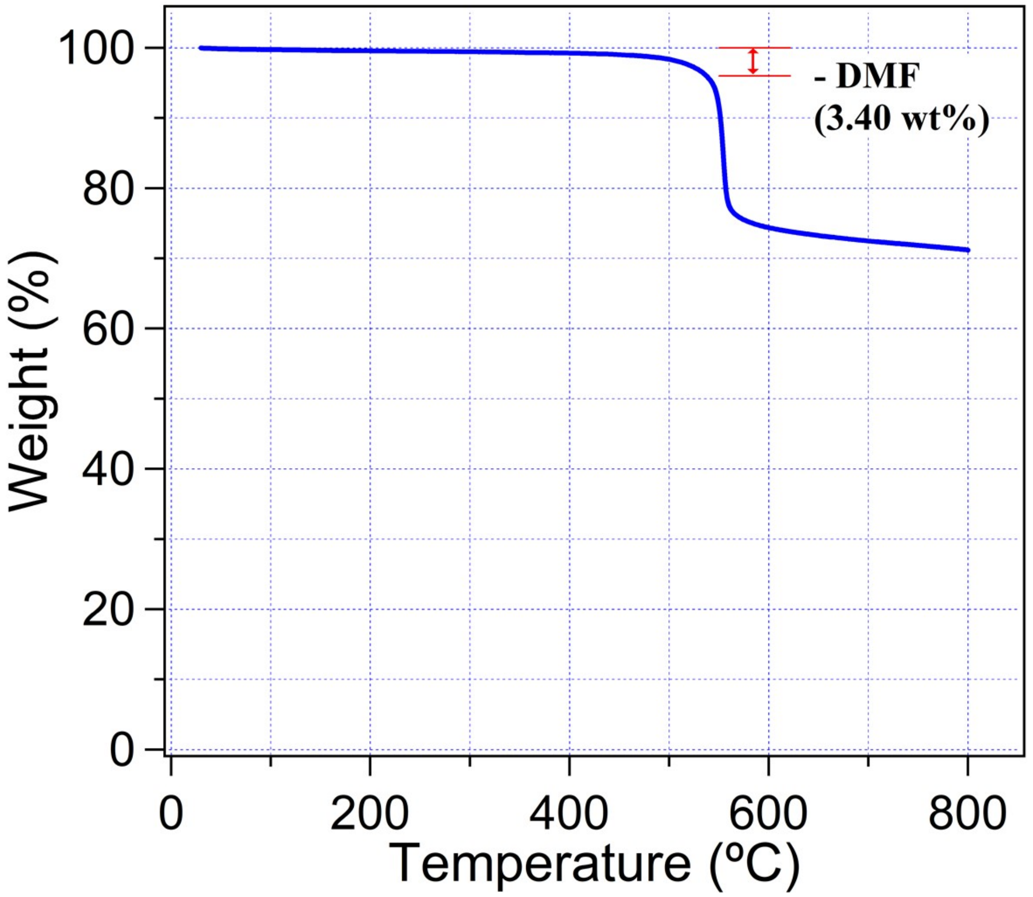

Figure S3. Thermogravimetric analysis curve of the as-prepared Zn-DpyDtolP-MOF.

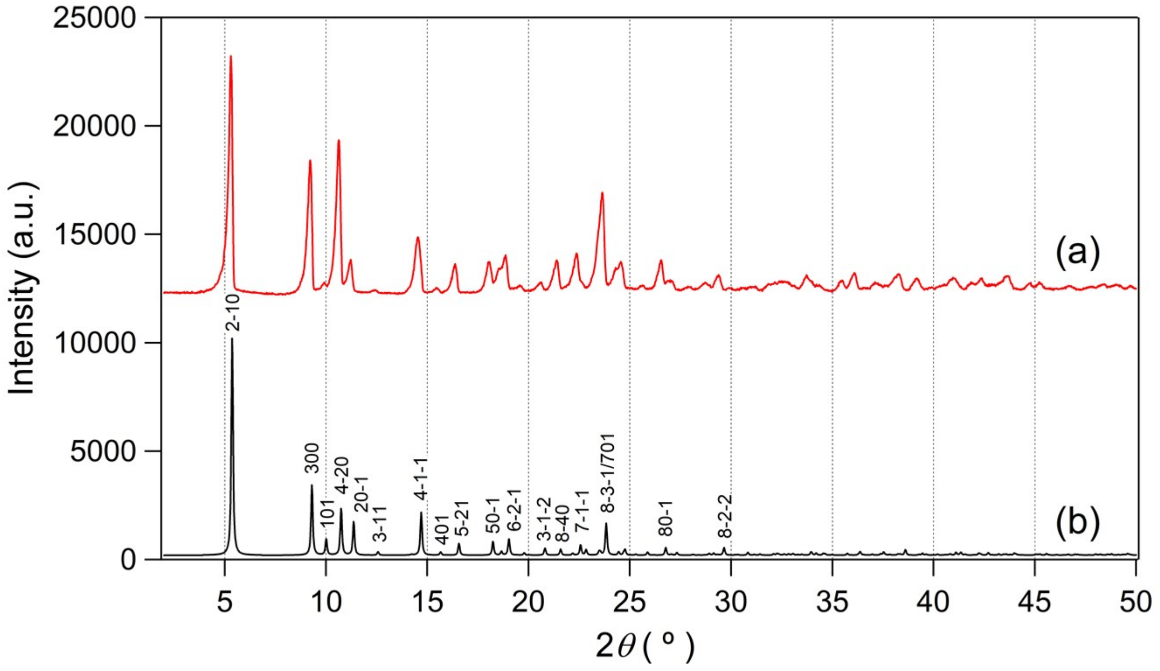

Figure S4. The PXRD pattern of the as-prepared Zn-DpyDtolP-MOF (a) and the simulated pattern from X-ray crystallography data (b).

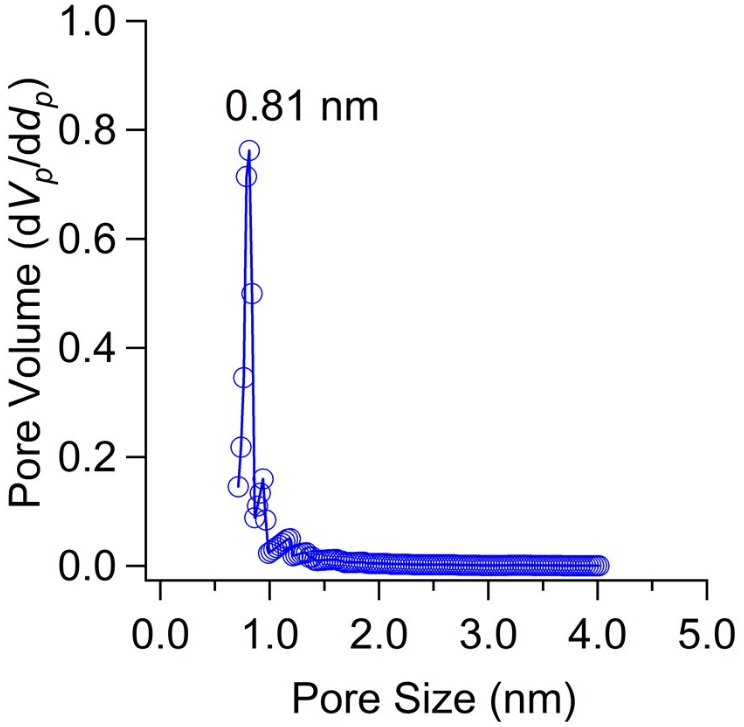

Figure S5. The pore size distribution curve of Zn-DpyDtolP-MOF calculated by Horváth-Kawazoe (HK) method.

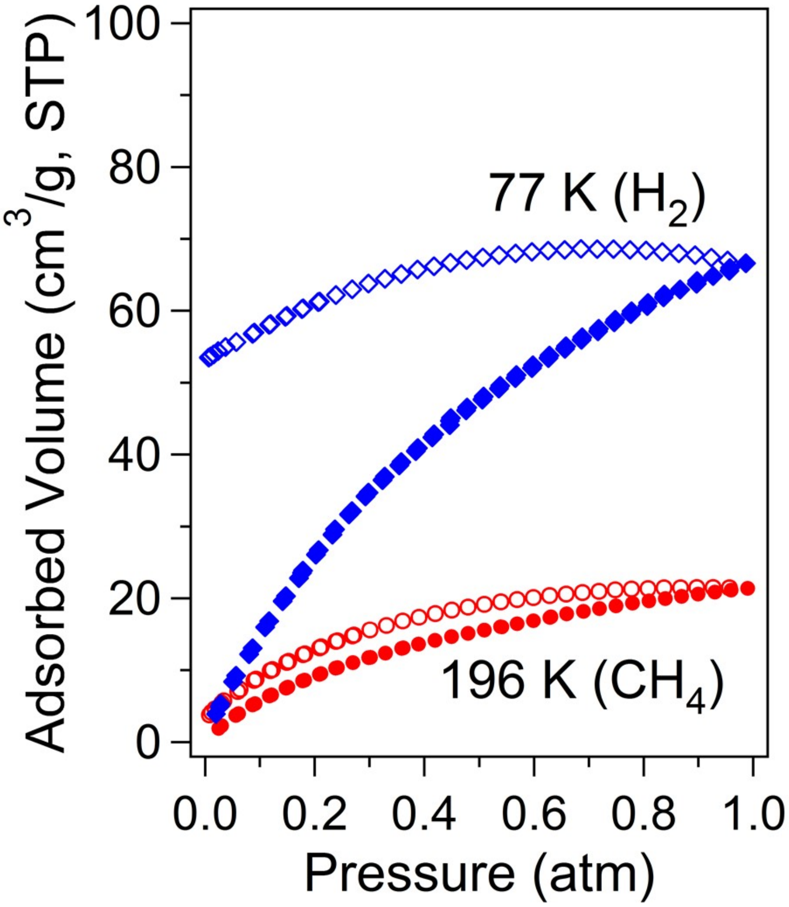

Figure S6. H_2 adsorption/desorption isotherms measured at 77 K and CH_4 adsorption/desorption isotherms at 196 K for solvent-free Zn-DpyDtolP-MOF. Open and solid symbols represent adsorption and desorption isotherms, respectively.

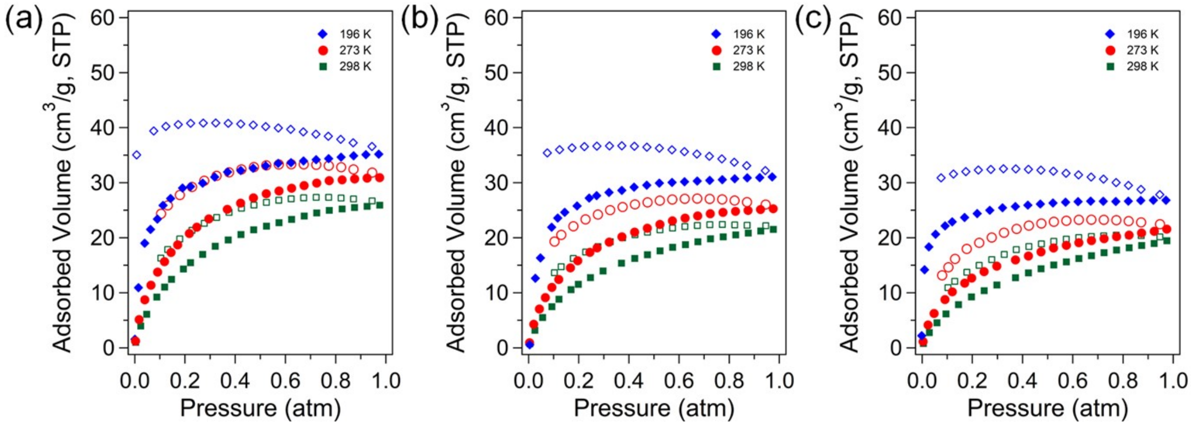

Figure S7. CO_2 adsorption/desorption isotherms measured at 196, 273, and 298 K for MDC-700 (a), MDC-800 (b), and MDC-900 (c). Open and solid symbols represent adsorption and desorption isotherms, respectively.

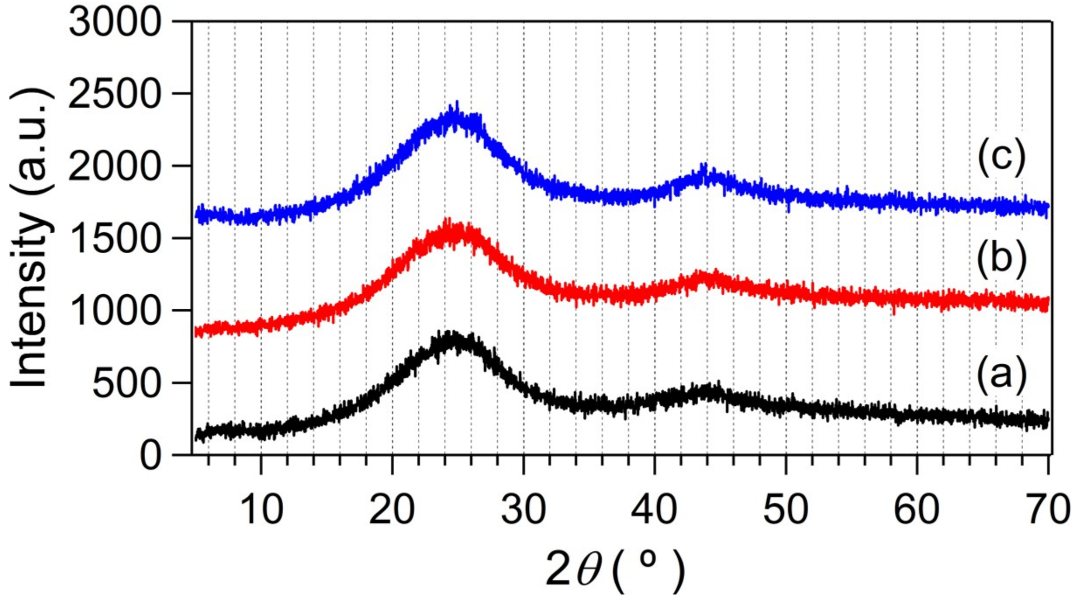

Figure S8. PXRD patterns of MDC-700 (a), MDC-800 (b), and MDC-900 (c).

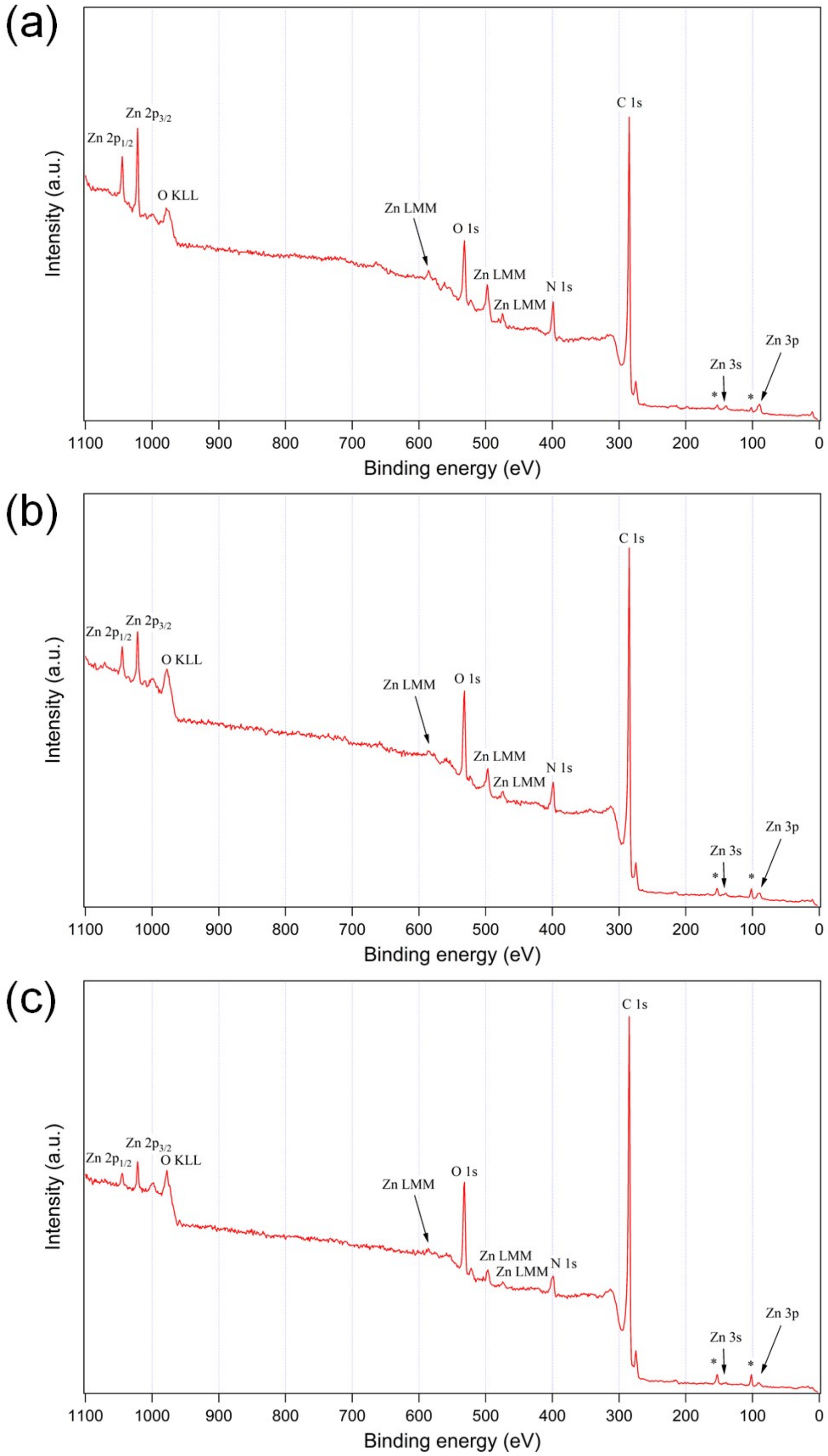
(a)
Zn 2p$_{1/2}$
Zn 2p$_{3/2}$
O KLL
Zn LMM
O 1s
Zn LMM
Zn LMM
N 1s
C 1s
Zn 3s
Zn 3p
*
*
Intensity (a.u.)
Binding energy (eV)
1100 1000 900 800 700 600 500 400 300 200 100 0
(b)
Zn 2p$_{1/2}$
Zn 2p$_{3/2}$
O KLL
Zn LMM
O 1s
Zn LMM
Zn LMM
N 1s
C 1s
Zn 3s
Zn 3p
*
*
Intensity (a.u.)
Binding energy (eV)
1100 1000 900 800 700 600 500 400 300 200 100 0
(c)
Zn 2p$_{1/2}$
Zn 2p$_{3/2}$
O KLL
Zn LMM
O 1s
Zn LMM
Zn LMM
N 1s
C 1s
Zn 3s
Zn 3p
*
*
Intensity (a.u.)
Binding energy (eV)
1100 1000 900 800 700 600 500 400 300 200 100 0

(d)

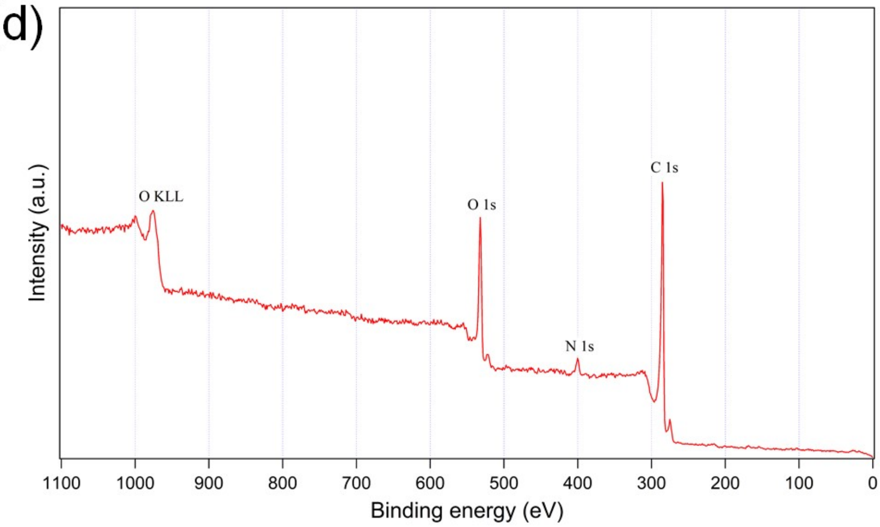

(e)

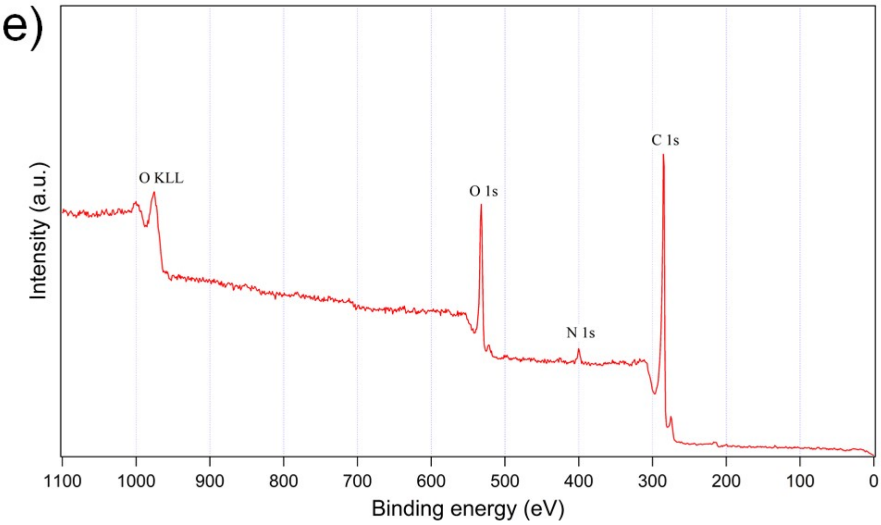

Figure S9. Survey XPS spectra of MDC-700 (a), MDC-800 (b), MDC-900 (c), MDC-700-1KOH (d), and MDC-700-2KOH (e).

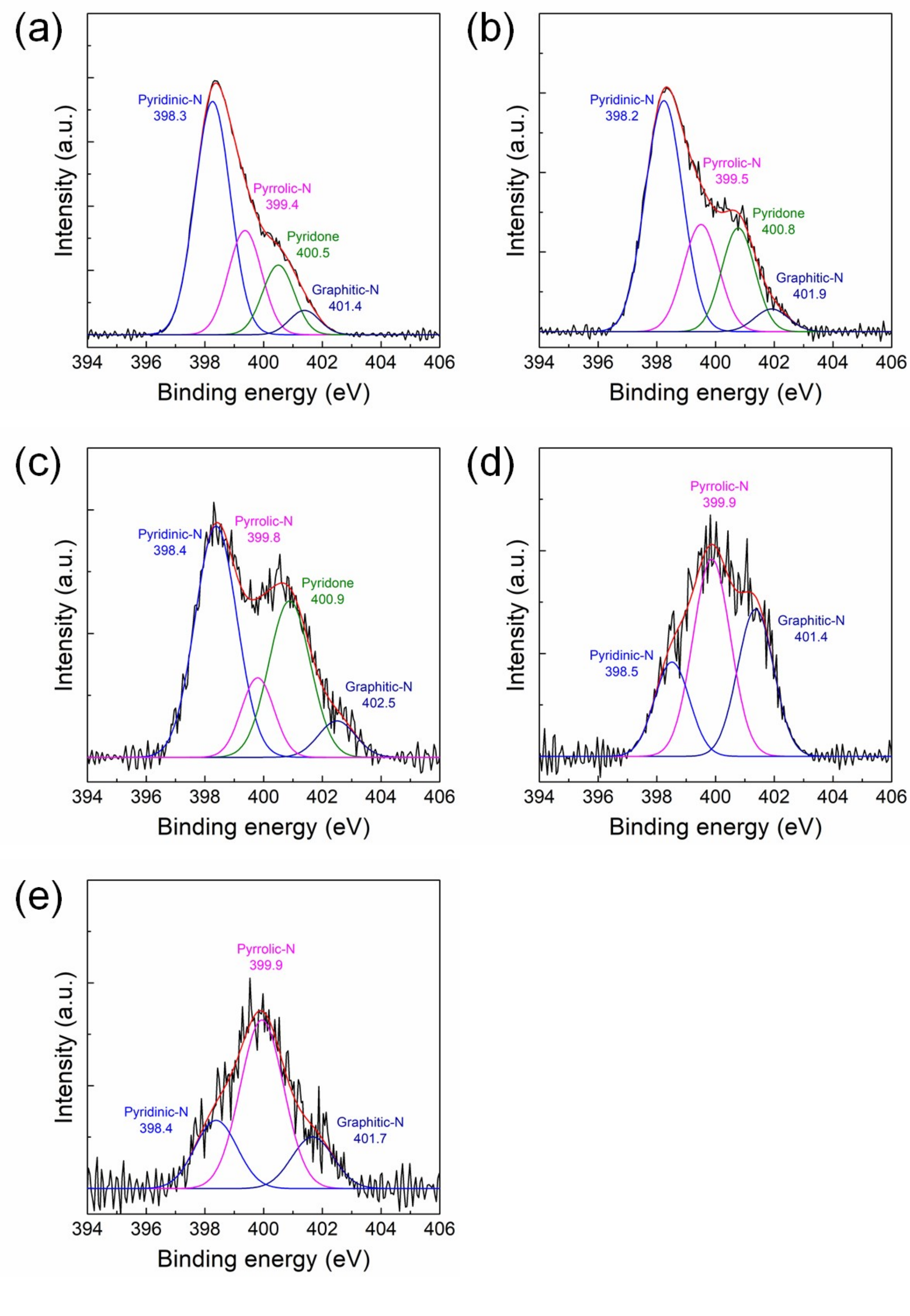

Figure S10. Deconvoluted high-resolution N1s XPS spectra of MDC-700 (a), MDC-800 (b), MDC-900 (c), MDC-700-1KOH (d), and MDC-700-2KOH (e).

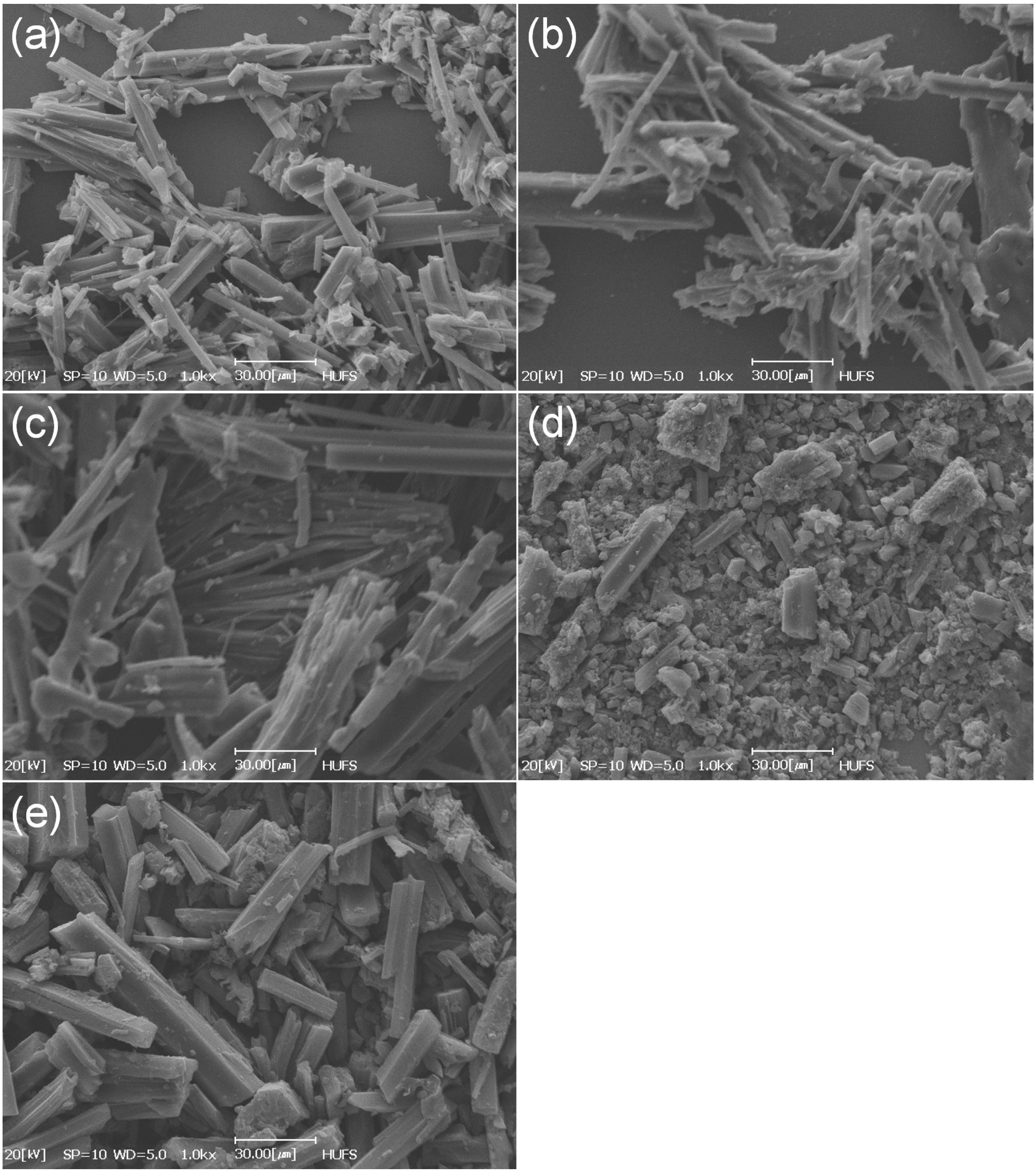

Figure S11. SEM images of MDC-700 (a), MDC-800 (b), MDC-900 (c), MDC-700-1KOH (d), and MDC-700-2KOH (e).

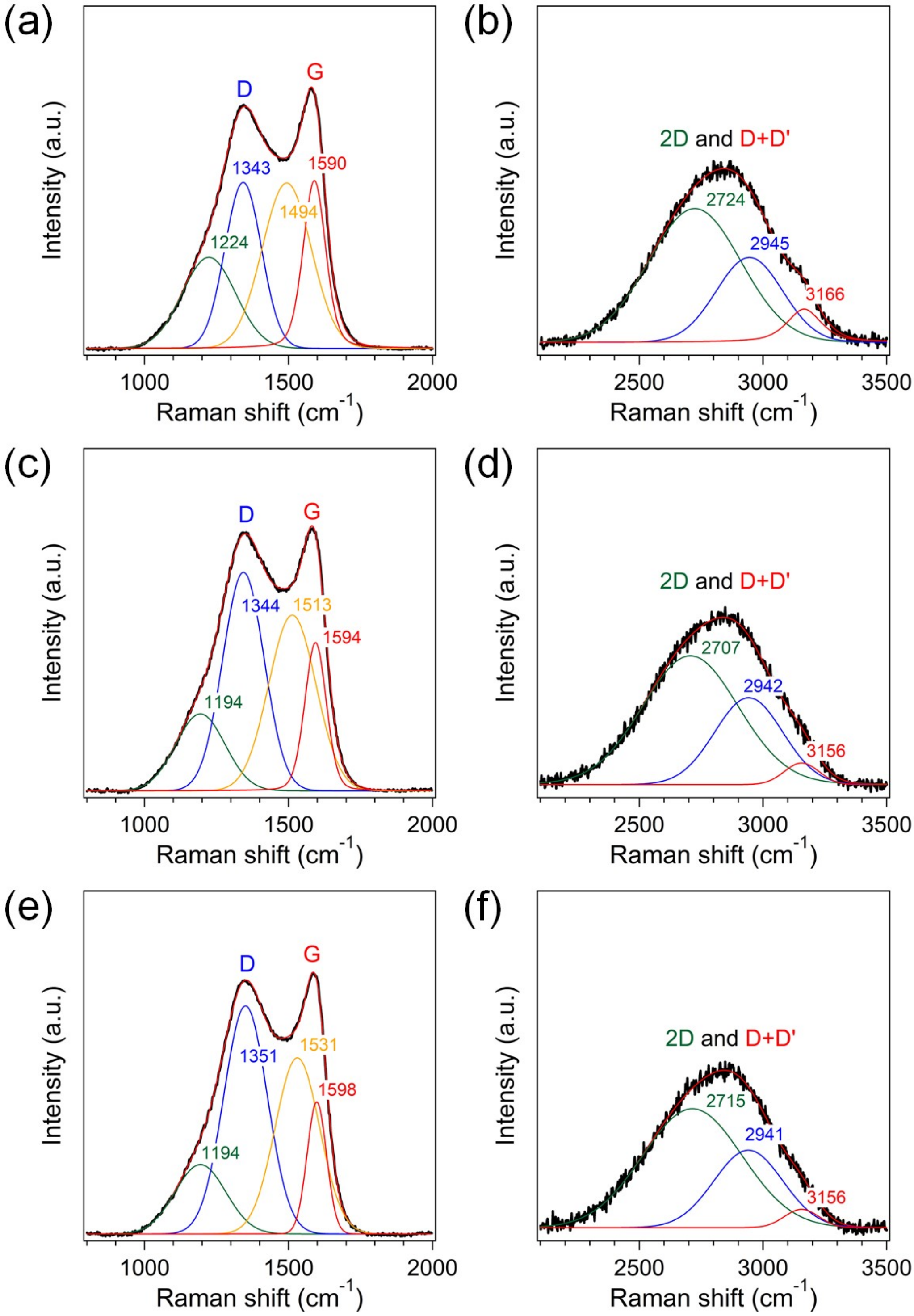

Figure S12. Raman spectra for MDC-700 (a, b), MDC-800 (c, d), and MDC-900 (e, f).

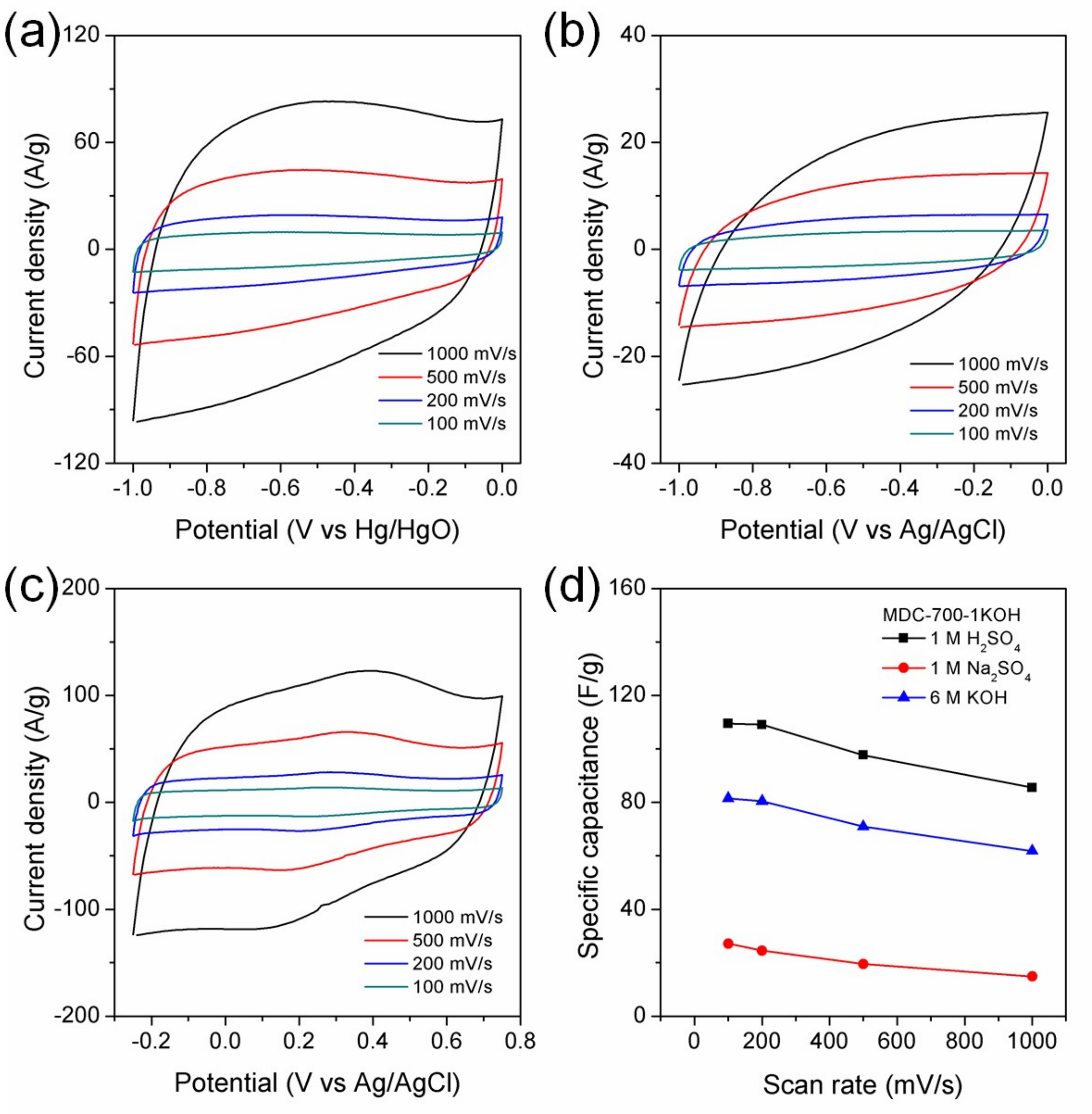

Figure S13. CV curves of MDC-700-1KOH using 3E system in 6 M KOH (a), 1 M Na_2SO_4 (b), and 1 M H_2SO_4 (c). (d) Dependence of specific capacitance on scan rate (potential window: -1.0 V ~ 0.0 V vs Hg/HgO in 6 M KOH, -1.0 V ~ 0.0 V vs Ag/AgCl in 1 M Na_2SO_4, -0.25 V ~ 0.75 V vs Ag/AgCl in 1 M H_2SO_4).

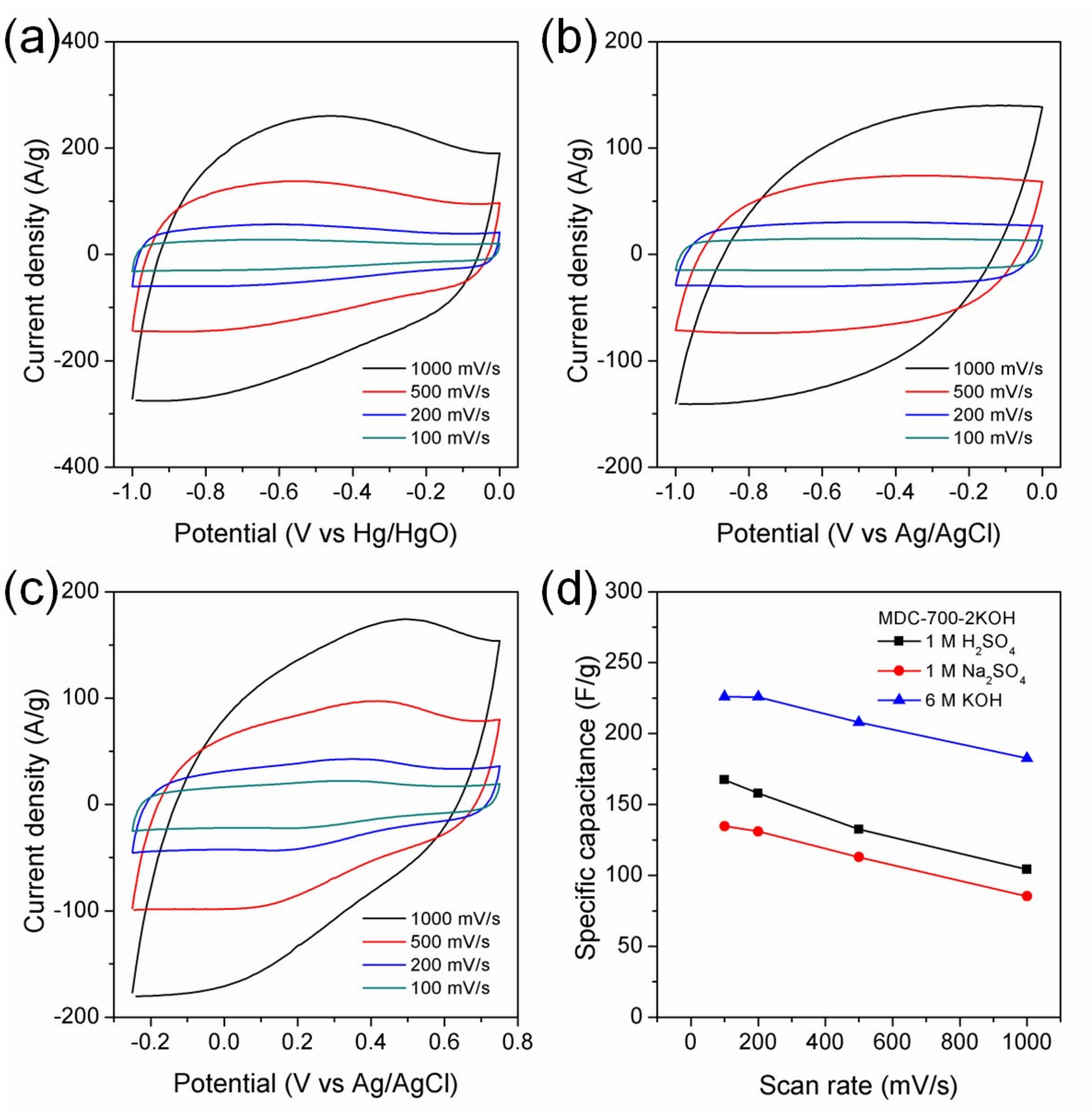

Figure S14. CV curves of MDC-700-2KOH using 3E system in 6 M KOH (a), 1 M Na_2SO_4 (b), and 1 M H_2SO_4 (c). (d) Dependence of specific capacitance on scan rate (potential window: -1.0 V ~ 0.0 V vs Hg/HgO in 6 M KOH, -1.0 V ~ 0.0 V vs Ag/AgCl for 1 M Na_2SO_4, -0.25 V ~ 0.75 V vs Ag/AgCl for 1 M H_2SO_4).

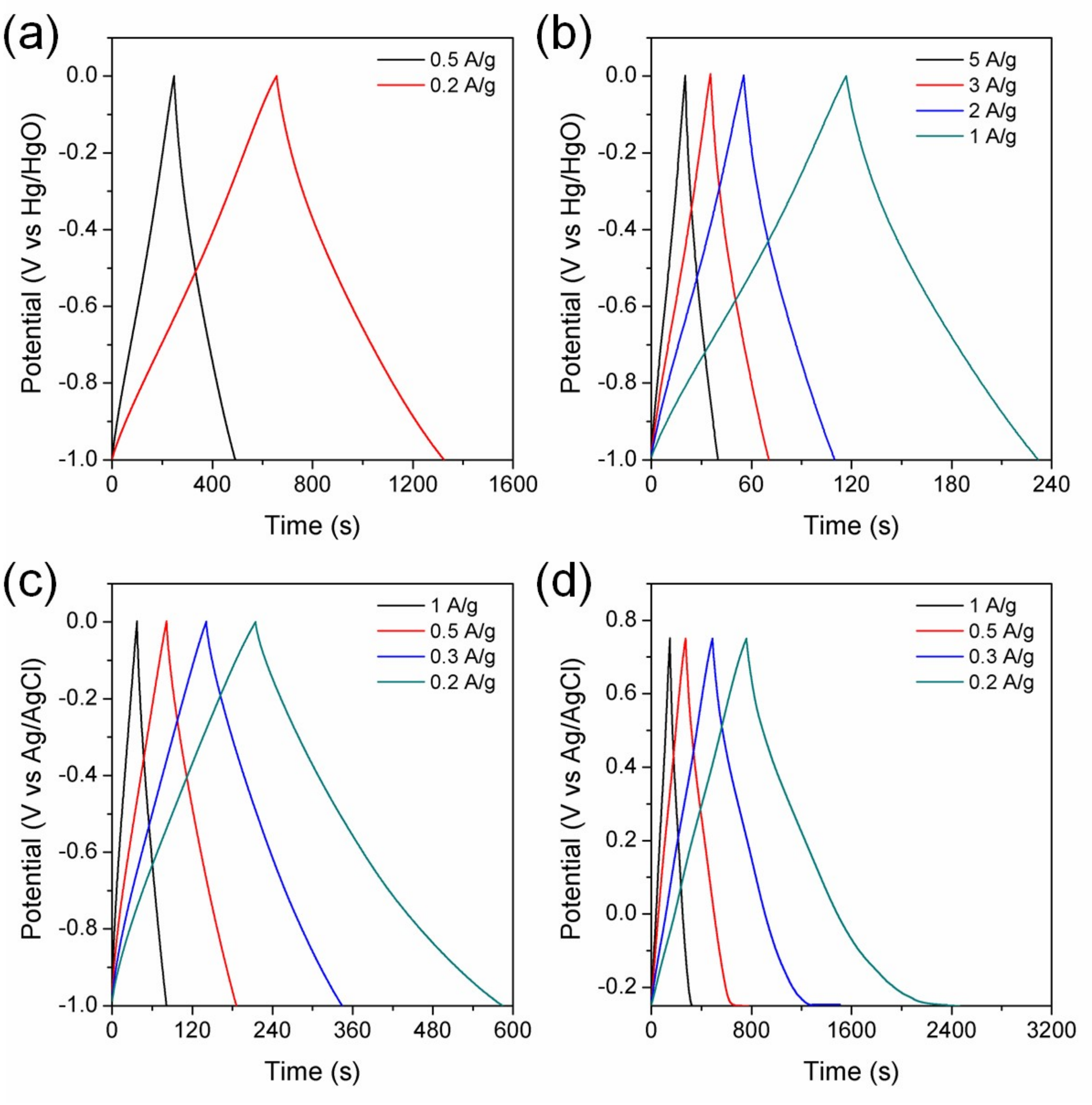

Figure S15. GCD curves for MDC-700-1KOH using 3E system in 6 M KOH (a and b), 1 M Na_2SO_4 (c), and 1 M H_2SO_4 (d).

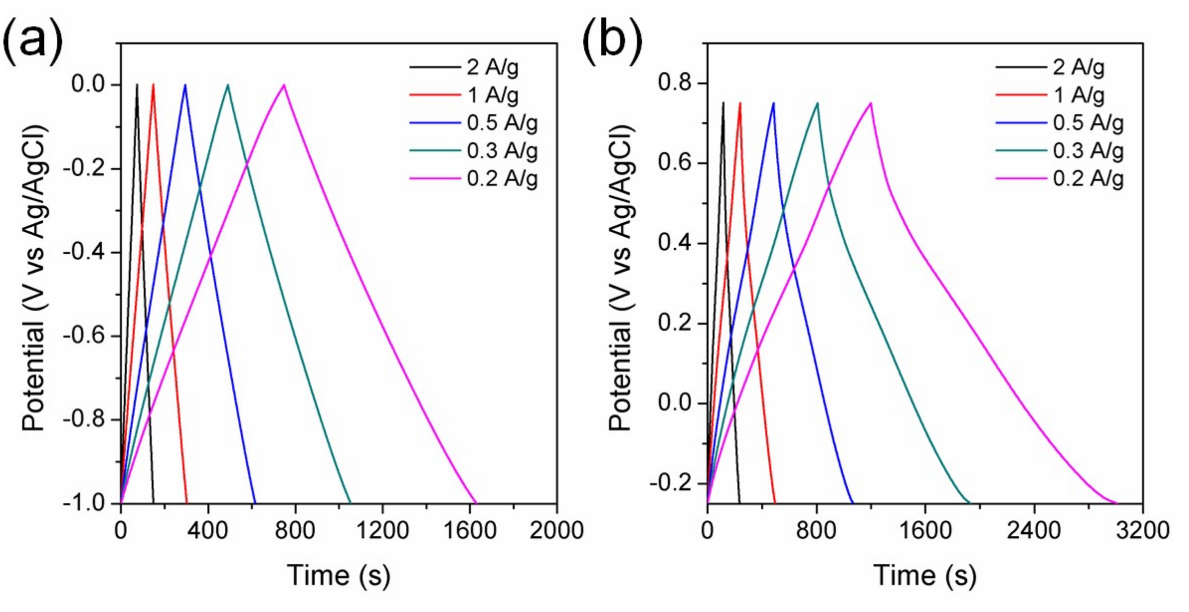

Figure S16. GCD curves for MDC-700-2KOH using 3E system in 1 M Na_2SO_4 (a) and 1 M H_2SO_4 (b).

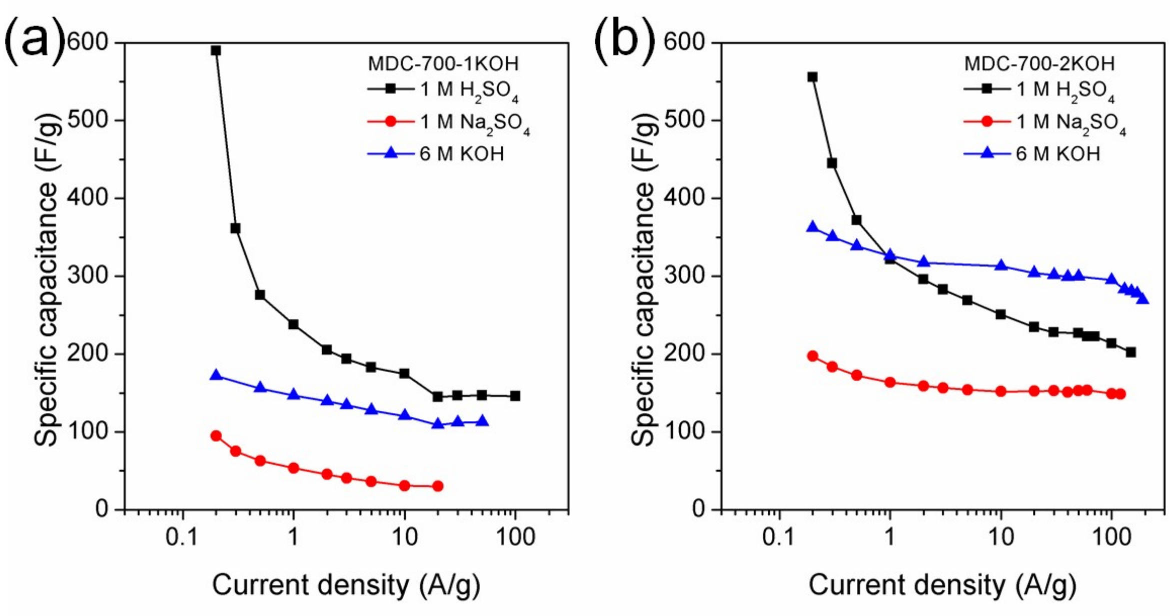

Figure S17. Dependence of specific capacitance on current density using 3E system in different electrolytes for MDC-700-1KOH (a) and for MDC-700-2KOH (b).

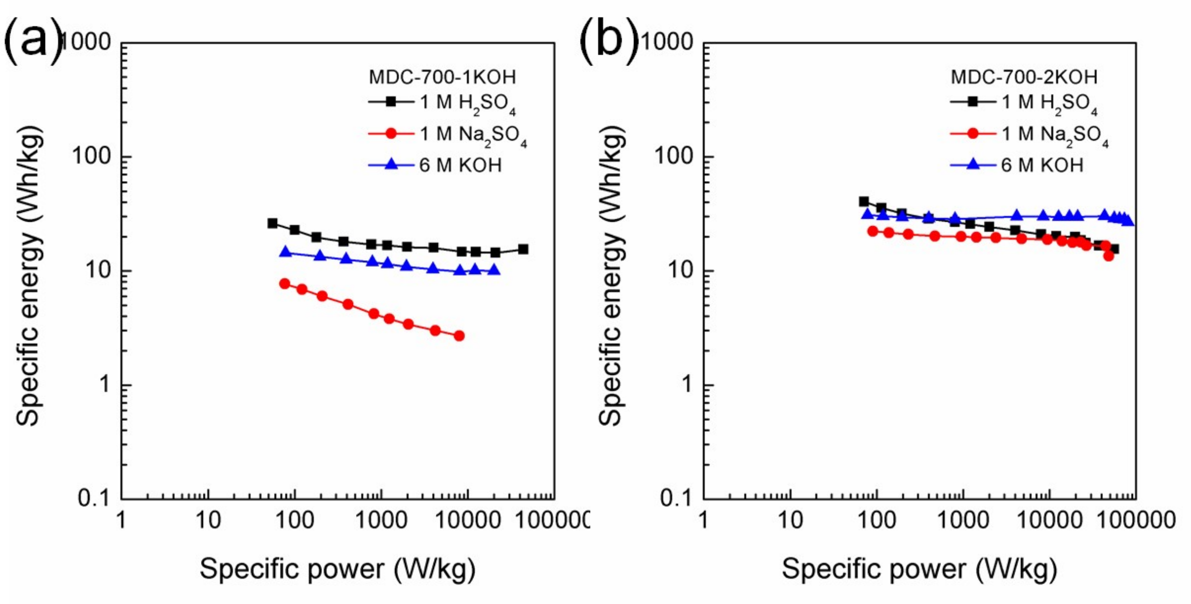

Figure S18. Ragone plots from the GCD curves using 3E system in different electrolytes for MDC-700-1KOH (a) and MDC-700-2KOH (b).

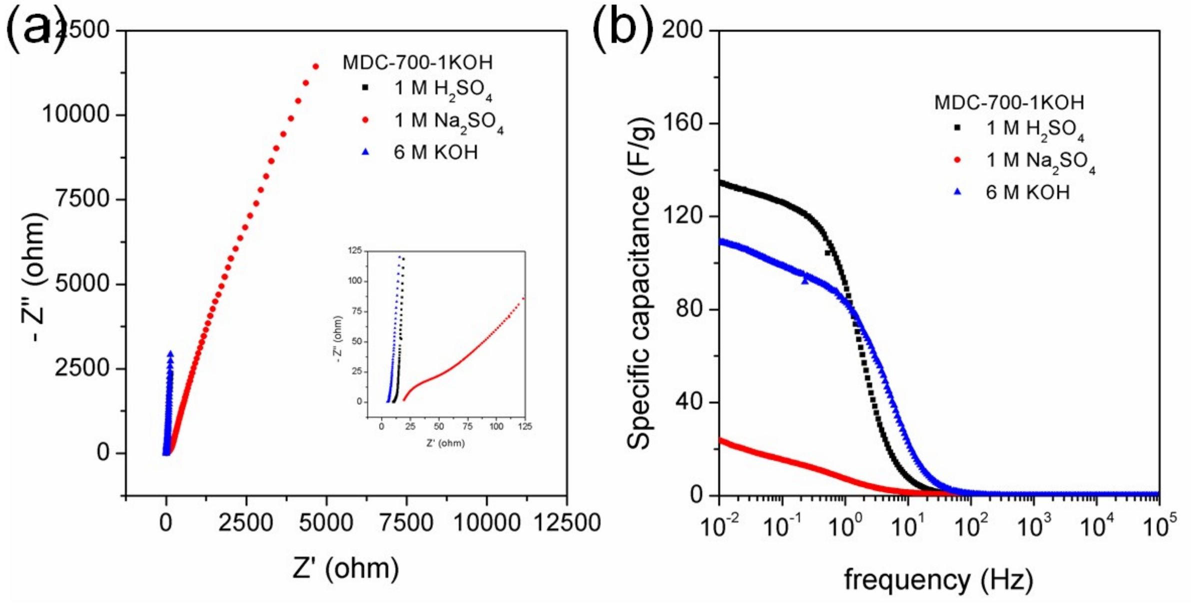

Figure S19. EIS plots for MDC-700-1KOH using 3E system in different electrolytes. Nyquist plots (a). Bode plots from the specific capacitance (b).

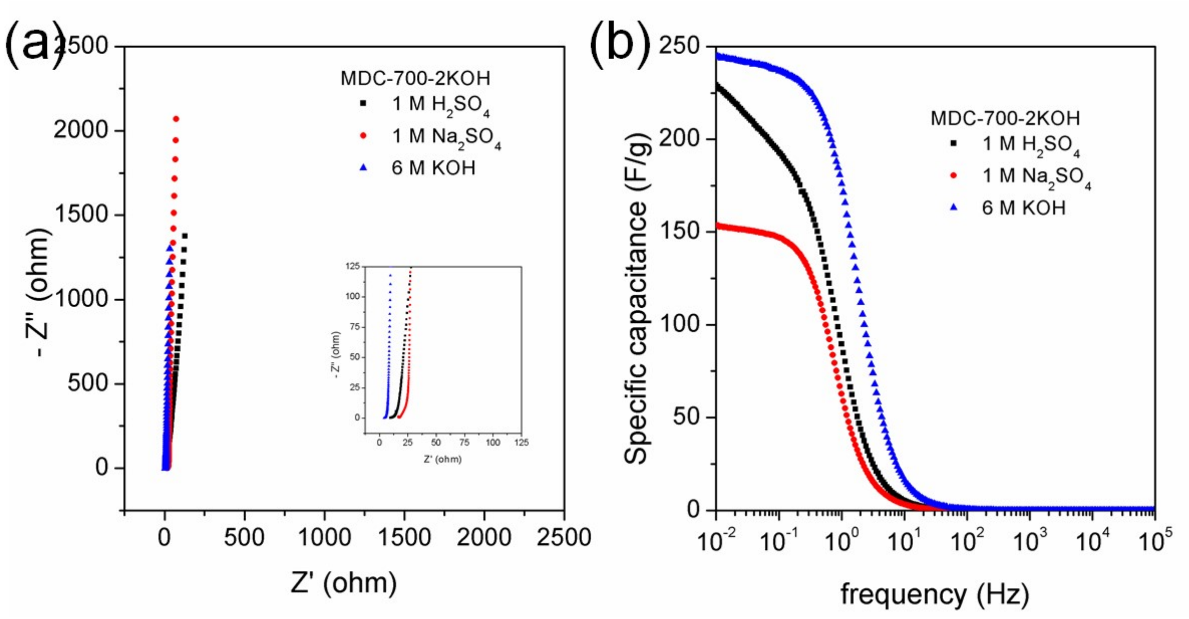

Figure S20. EIS plots for MDC-700-2KOH using 3E system in different electrolytes. Nyquist plots (a). Bode plots from the specific capacitance (b).

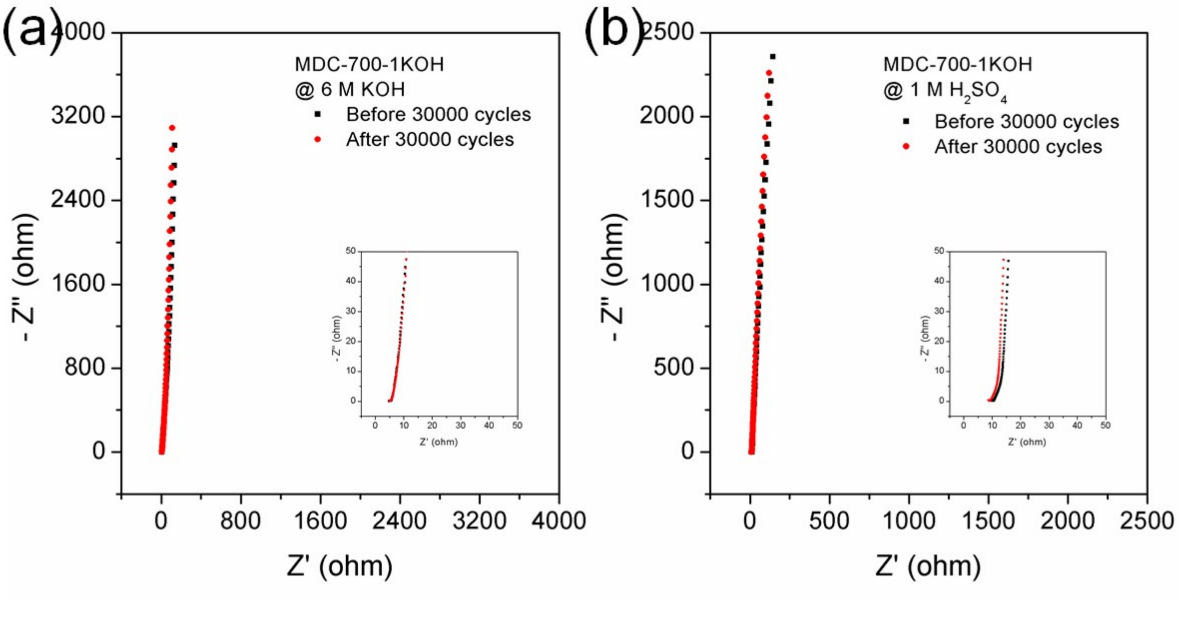

Figure S21. Nyquist plots for MDC-700-1KOH using 3E system before and after 30000 cycles in 6 M KOH (a) and 1 M H_2SO_4 (b).

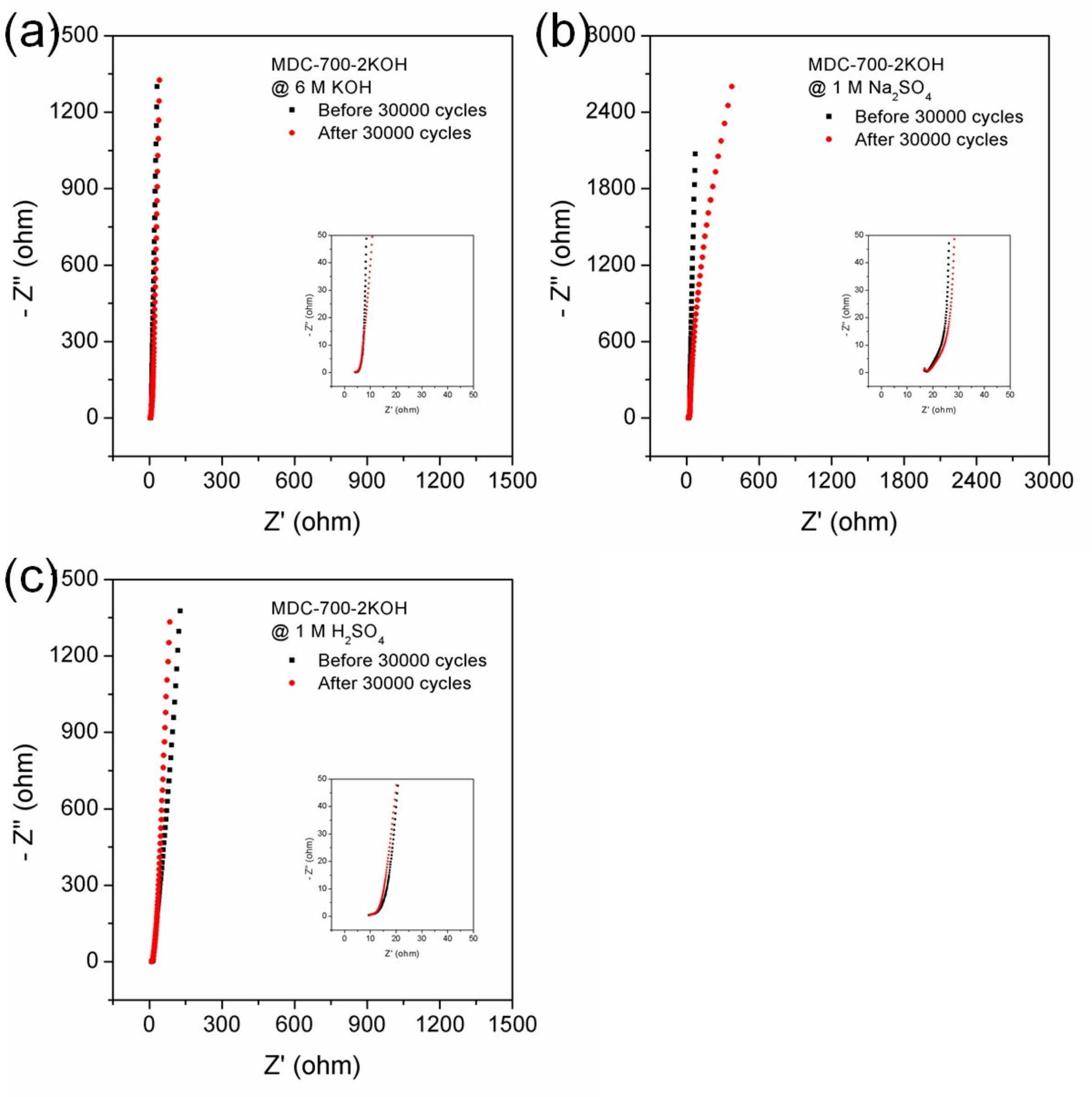

Figure S22. Nyquist plots for MDC-700-2KOH using 3E system before and after 30000 cycles in 6 M KOH (a), 1 M Na_2SO_4 (b), and 1 M H_2SO_4 (c).

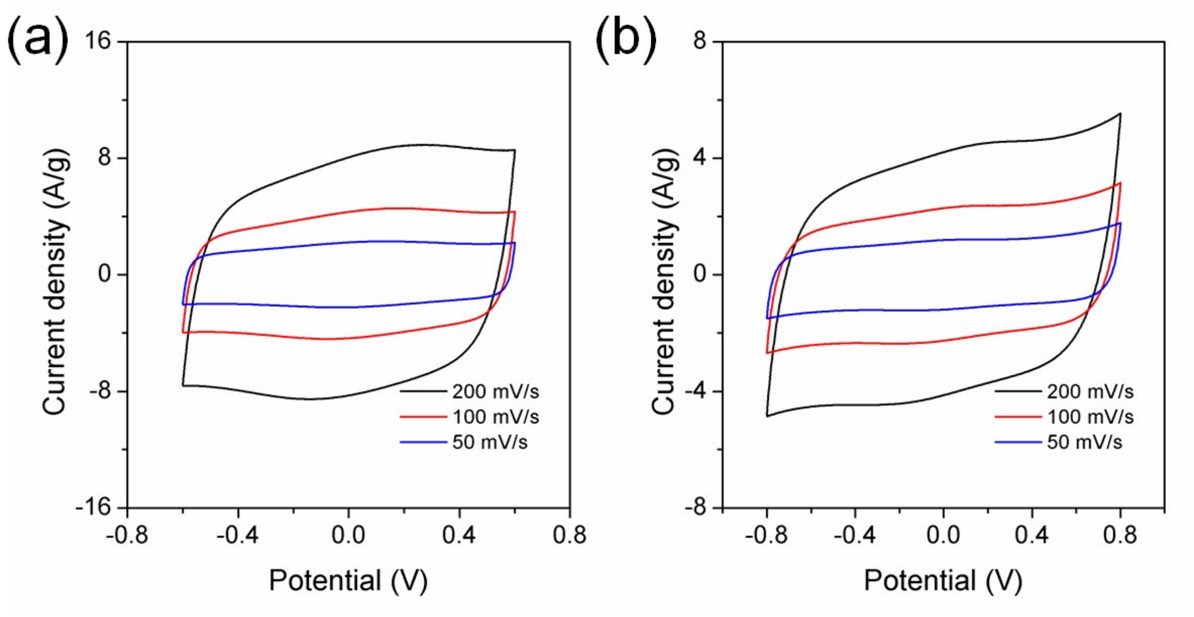

Figure S23. CV curves for MDC-700-2KOH using Swagelok cell-based 2E system in 1 M KOH (a) and 1 M Na_2SO_4 (b).

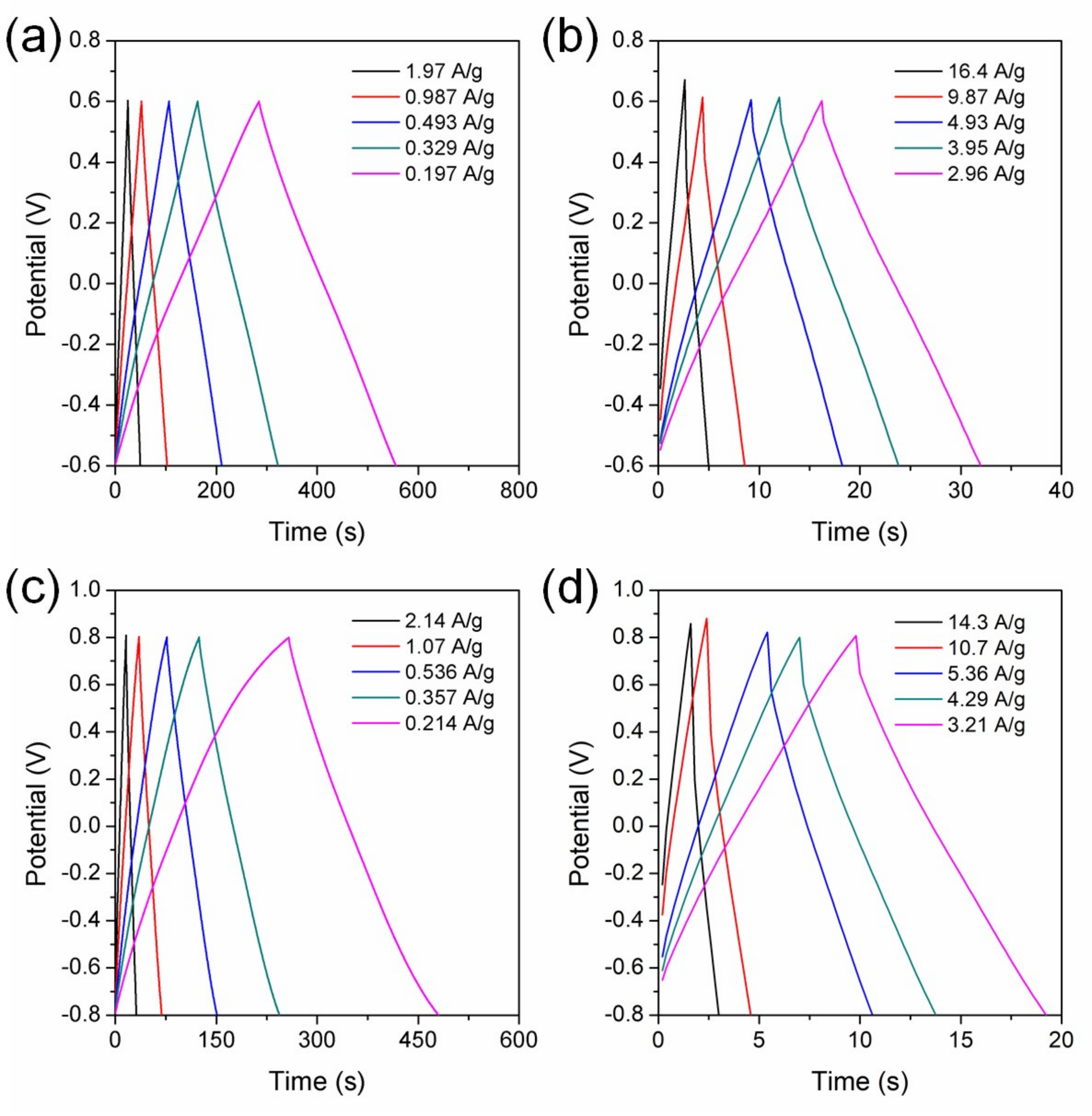

Figure S24. GCD curves for MDC-700-2KOH using Swagelok cell-based 2E system in 1 M KOH (a and b) and 1 M Na_2SO_4 (c and d).

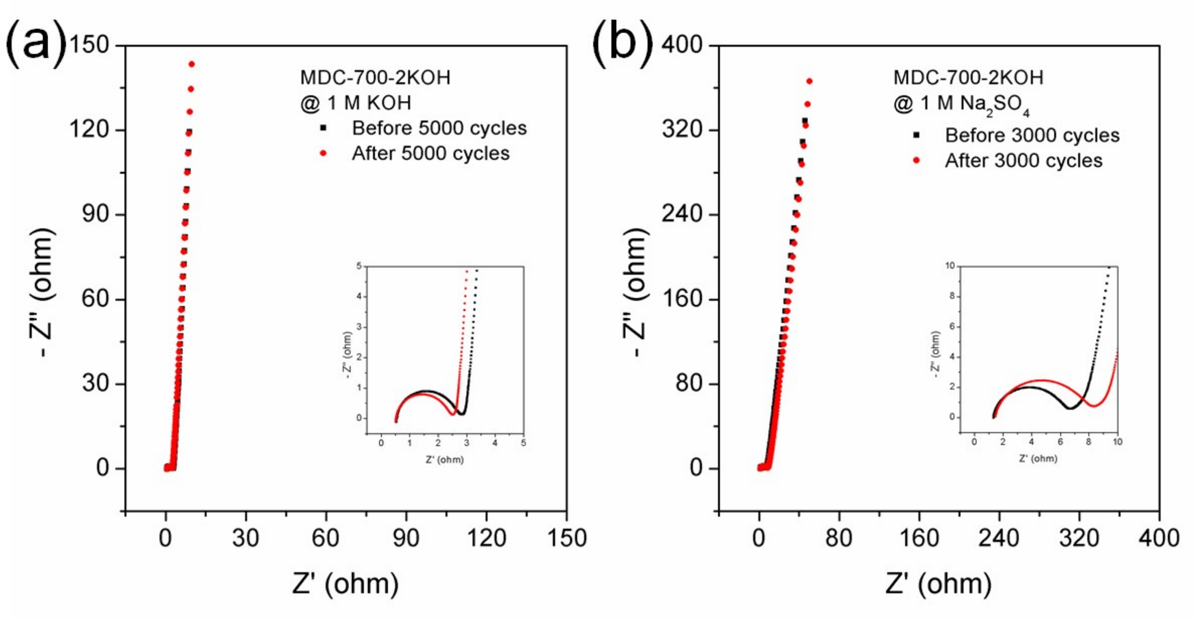

Figure S25. Nyquist plots for MDC-700-2KOH using Swagelok cell-based 2E system in 1 M KOH (a) and 1 M Na_2SO_4 (b).

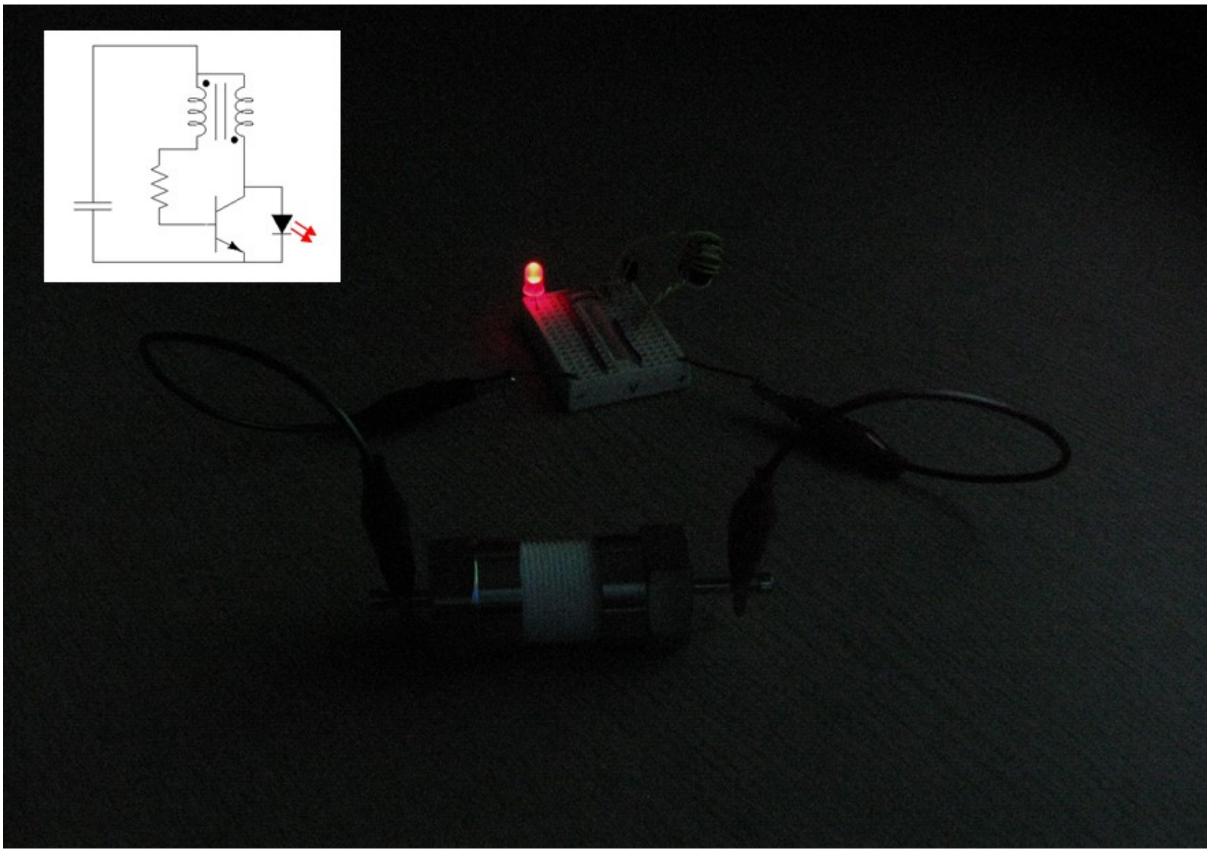

Figure S26. Digital photo image of the charged MDC-700-2KOH 2E capacitor lighting the red light-emitting diode which can operate at above 1.20 V. The electrolyte is 1.0 M Na_2SO_4. The minimum operation voltage of the red LED is controlled by the Joule thief circuit. Inset indicates the corresponding circuit diagram.

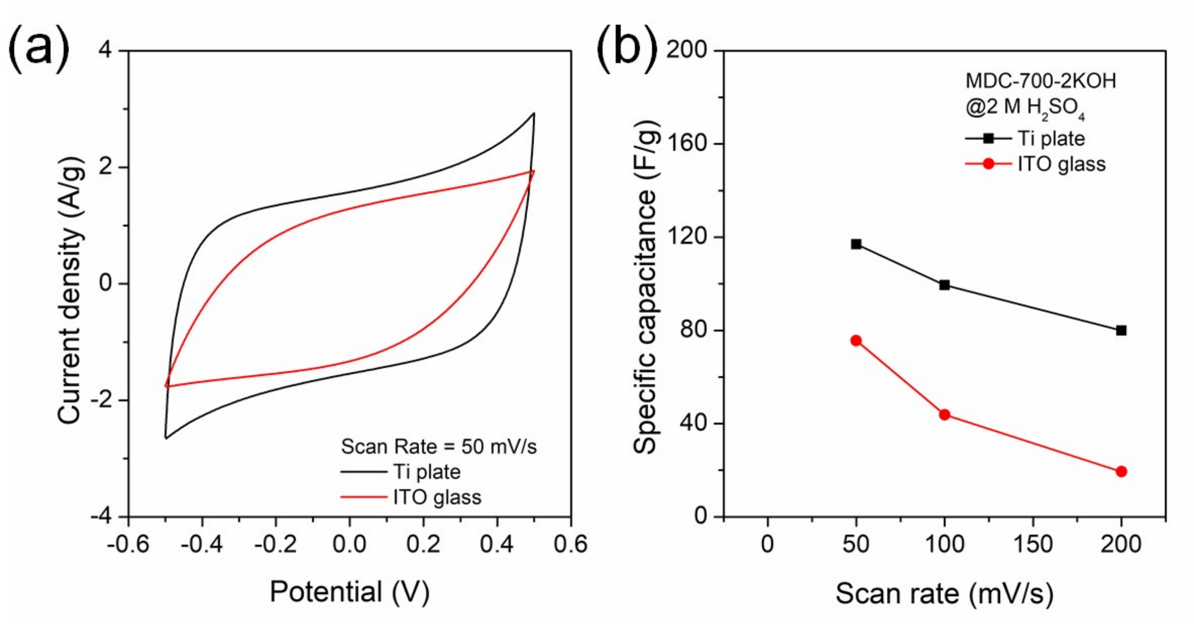

Figure S27. CV results for MDC-700-2KOH using ITO glass-based or Ti plate-based 2E systems in 2 M H_2SO_4 electrolyte (potential window: -0.5 V ~ 0.5 V). CV curves at 100 mV s^{-1} (a). Dependence of specific capacitance on scan rate (b).

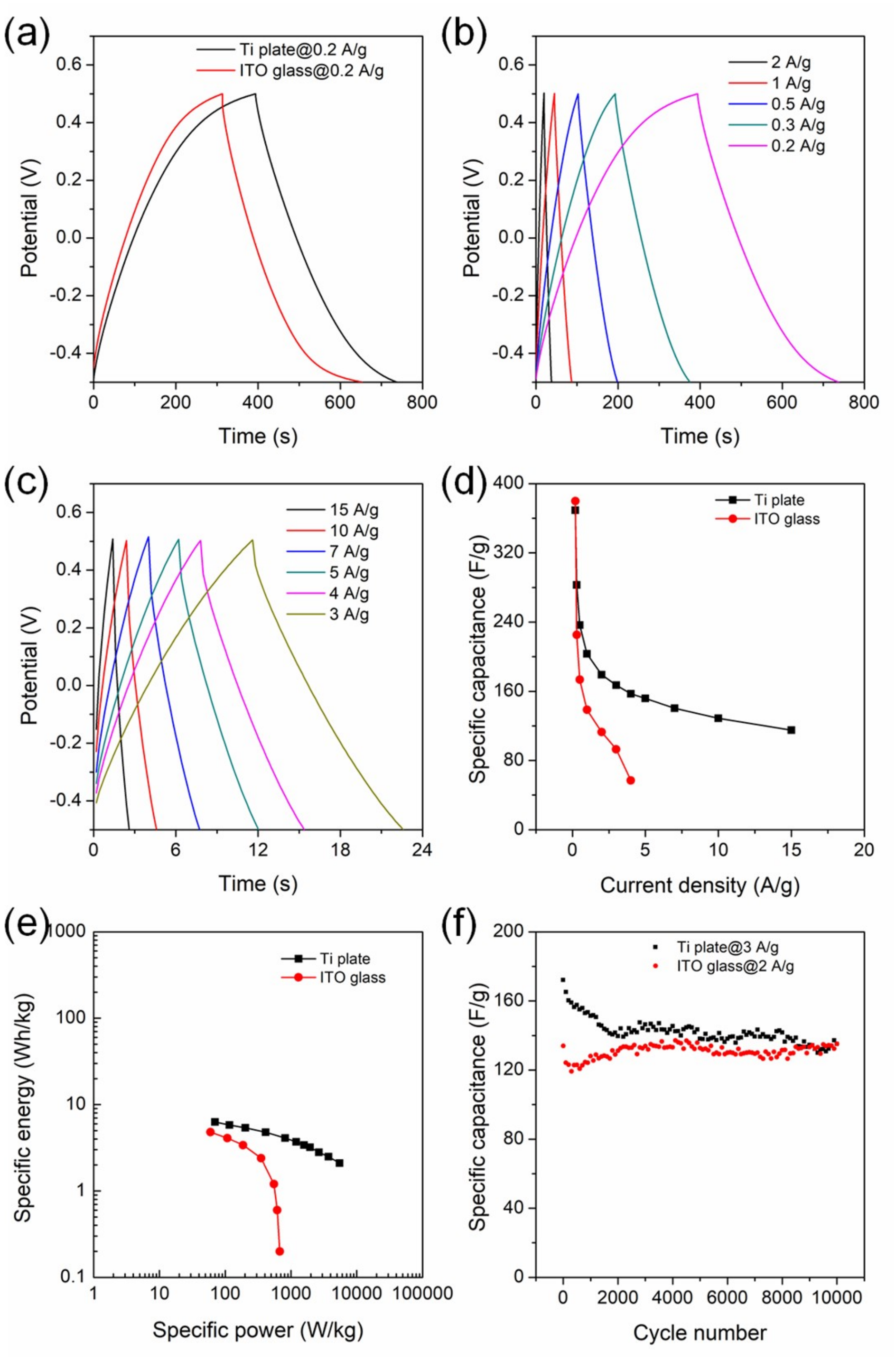

Figure S28. GCD results for MDC-700-2KOH using 2E systems in 2 M H_2SO_4 electrolyte (potential window: -0.5 V ~ 0.5 V). GCD curves at 0.2 A g^{-1} (a). GCD curves at low current densities using Ti plate-based system (b). GCD curves at high current densities under Ti plate-based systems (c). The dependence of specific capacitance on current density (d). Ragone plots (e). The variation of specific capacitance as a function of cycle number (f).

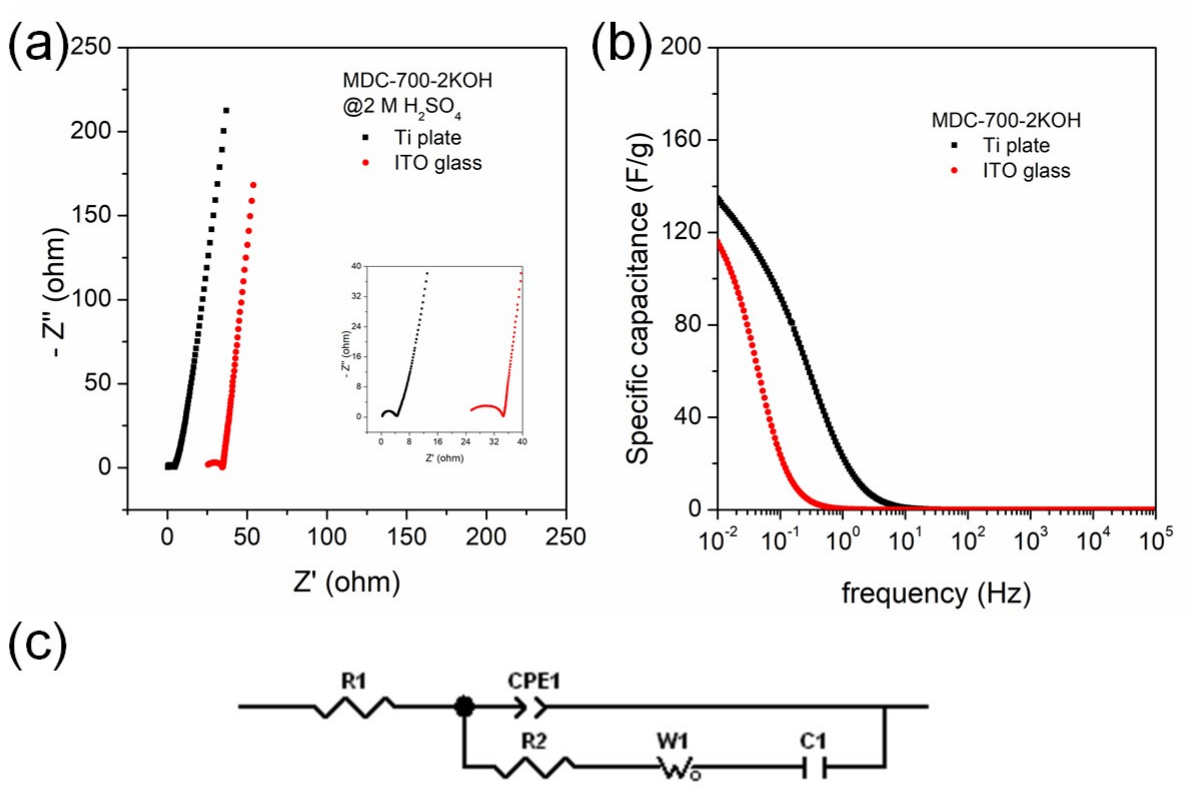

Figure S29. EIS results for MDC-700-2KOH using ITO glass-based or Ti plate-based 2E systems in 2 M H_2SO_4 electrolyte (applied constant potential: 0 V). Nyquist plots (a). Bode plots from specific capacitance (b). The corresponding equivalent circuit (c).

Table S1. Crystal data and structure refinement for Zn-DpyDtolP-MOF.

Empirical formula	C44 H30 N6 Zn	
Formula weight	708.11	
Temperature	100(2) K	
Wavelength	1.54178 Å	
Crystal system	Trigonal	
Space group	R -3	
Unit cell dimensions	a = 32.9136(15) Å	α= 90°.
	b = 32.9136(15) Å	β= 90°.
	c = 9.2886(5) Å	γ = 120°.
Volume	8714.3(9) Å^3	
Z	9	
Density (calculated)	1.214 Mg/m^3	
Absorption coefficient	1.167 mm^{-1}	
F(000)	3294	
Crystal size	0.01 x 0.01 x 0.08 mm^3	
Theta range for data collection	2.685 to 78.955°.	
Index ranges	-41<=h<=41, -39<=k<=41, -11<=l<=11	
Reflections collected	32296	
Independent reflections	4118 [R(int) = 0.0504]	
Completeness to theta = 67.679°	100.0 %	
Refinement method	Full-matrix least-squares on F^2	
Data / restraints / parameters	4118 / 0 / 233	
Goodness-of-fit on F^2	1.057	
Final R indices [I>2sigma(I)]	R1 = 0.0413, wR2 = 0.1071	
R indices (all data)	R1 = 0.0535, wR2 = 0.1135	
Largest diff. peak and hole	0.448 and -0.338 e.Å^{-3}	

Table S2. Atomic coordinates (Å × 10^4) and equivalent isotropic displacement parameters (Å^2 × 10^3) for Zn-DpyDtolP-MOF. U(eq) is defined as one third of the trace of the orthogonalized U^{ij} tensor.

	x	y	z	U(eq)
Zn(1)	5000	0	0	14(1)
N(1)	4992(1)	591(1)	718(2)	14(1)
N(2)	5344(1)	11(1)	1890(2)	15(1)
N(3)	5756(1)	434(1)	-911(2)	18(1)
C(1)	4747(1)	775(1)	79(2)	16(1)
C(2)	4773(1)	1149(1)	964(2)	20(1)
C(3)	5027(1)	1178(1)	2149(2)	19(1)
C(4)	5161(1)	822(1)	1998(2)	15(1)
C(5)	5410(1)	720(1)	3038(2)	16(1)
C(6)	5505(1)	347(1)	2944(2)	16(1)
C(7)	5816(1)	283(1)	3892(2)	21(1)
C(8)	5847(1)	-84(1)	3384(2)	22(1)
C(9)	5551(1)	-255(1)	2113(2)	16(1)
C(10)	5505(1)	-619(1)	1219(2)	16(1)
C(11)	5590(1)	1033(1)	4323(2)	17(1)
C(12)	5433(1)	856(1)	5700(2)	20(1)
C(13)	5594(1)	1149(1)	6897(2)	24(1)
C(14)	5919(1)	1625(1)	6760(3)	27(1)
C(15)	6078(1)	1799(1)	5385(3)	28(1)
C(16)	5917(1)	1510(1)	4184(2)	23(1)
C(17)	6101(1)	1933(1)	8081(3)	41(1)
C(18)	6707(1)	906(1)	-1671(2)	17(1)
C(19)	6544(1)	1065(1)	-556(2)	21(1)
C(20)	6070(1)	821(1)	-219(2)	21(1)
C(21)	5915(1)	278(1)	-1975(2)	20(1)
C(22)	6381(1)	500(1)	-2391(2)	20(1)

Table S3. Bond lengths [Å] and angles [°] for Zn-DpyDtolP-MOF.

Zn(1)-N(1)#1	2.0683(16)
Zn(1)-N(1)	2.0683(16)
Zn(1)-N(2)#1	2.0789(16)
Zn(1)-N(2)	2.0790(16)
Zn(1)-N(3)	2.3223(16)
Zn(1)-N(3)#1	2.3224(16)
N(1)-C(1)	1.363(2)
N(1)-C(4)	1.371(2)
N(2)-C(9)	1.367(2)
N(2)-C(6)	1.371(3)
N(3)-C(21)	1.334(3)
N(3)-C(20)	1.337(3)
C(1)-C(10)#1	1.408(3)
C(1)-C(2)	1.448(3)
C(2)-C(3)	1.355(3)
C(2)-H(2)	0.9500
C(3)-C(4)	1.450(3)
C(3)-H(3)	0.9500
C(4)-C(5)	1.413(3)
C(5)-C(6)	1.412(3)
C(5)-C(11)	1.493(3)
C(6)-C(7)	1.443(3)
C(7)-C(8)	1.348(3)
C(7)-H(7)	0.9500
C(8)-C(9)	1.452(3)
C(8)-H(8)	0.9500
C(9)-C(10)	1.403(3)
C(10)-C(1)#1	1.408(3)
C(10)-C(18)#2	1.487(3)
C(11)-C(12)	1.394(3)
C(11)-C(16)	1.395(3)
C(12)-C(13)	1.392(3)
C(12)-H(12)	0.9500
C(13)-C(14)	1.391(3)
C(13)-H(13)	0.9500

C(14)-C(15)	1.391(3)
C(14)-C(17)	1.513(3)
C(15)-C(16)	1.388(3)
C(15)-H(15)	0.9500
C(16)-H(16)	0.9500
C(17)-H(17A)	0.9800
C(17)-H(17B)	0.9800
C(17)-H(17C)	0.9800
C(18)-C(19)	1.385(3)
C(18)-C(22)	1.397(3)
C(18)-C(10)#3	1.487(3)
C(19)-C(20)	1.388(3)
C(19)-H(19)	0.9500
C(20)-H(20)	0.9500
C(21)-C(22)	1.385(3)
C(21)-H(21)	0.9500
C(22)-H(22)	0.9500

N(1)#1-Zn(1)-N(1)	180.0
N(1)#1-Zn(1)-N(2)#1	88.53(6)
N(1)-Zn(1)-N(2)#1	91.47(6)
N(1)#1-Zn(1)-N(2)	91.47(6)
N(1)-Zn(1)-N(2)	88.53(6)
N(2)#1-Zn(1)-N(2)	180.00(8)
N(1)#1-Zn(1)-N(3)	86.99(6)
N(1)-Zn(1)-N(3)	93.01(6)
N(2)#1-Zn(1)-N(3)	96.28(6)
N(2)-Zn(1)-N(3)	83.72(6)
N(1)#1-Zn(1)-N(3)#1	93.01(6)
N(1)-Zn(1)-N(3)#1	86.98(6)
N(2)#1-Zn(1)-N(3)#1	83.72(6)
N(2)-Zn(1)-N(3)#1	96.28(6)
N(3)-Zn(1)-N(3)#1	180.0
C(1)-N(1)-C(4)	107.22(16)
C(1)-N(1)-Zn(1)	124.44(13)
C(4)-N(1)-Zn(1)	127.61(13)
C(9)-N(2)-C(6)	107.03(16)

C(9)-N(2)-Zn(1)	123.95(13)
C(6)-N(2)-Zn(1)	127.18(13)
C(21)-N(3)-C(20)	117.27(17)
C(21)-N(3)-Zn(1)	122.58(13)
C(20)-N(3)-Zn(1)	119.38(13)
N(1)-C(1)-C(10)#1	125.76(18)
N(1)-C(1)-C(2)	109.52(17)
C(10)#1-C(1)-C(2)	124.69(18)
C(3)-C(2)-C(1)	107.05(18)
C(3)-C(2)-H(2)	126.5
C(1)-C(2)-H(2)	126.5
C(2)-C(3)-C(4)	106.86(18)
C(2)-C(3)-H(3)	126.6
C(4)-C(3)-H(3)	126.6
N(1)-C(4)-C(5)	125.62(18)
N(1)-C(4)-C(3)	109.31(17)
C(5)-C(4)-C(3)	125.02(18)
C(6)-C(5)-C(4)	124.91(18)
C(6)-C(5)-C(11)	117.76(17)
C(4)-C(5)-C(11)	117.33(17)
N(2)-C(6)-C(5)	125.56(18)
N(2)-C(6)-C(7)	109.50(17)
C(5)-C(6)-C(7)	124.80(18)
C(8)-C(7)-C(6)	107.11(18)
C(8)-C(7)-H(7)	126.4
C(6)-C(7)-H(7)	126.4
C(7)-C(8)-C(9)	107.16(18)
C(7)-C(8)-H(8)	126.4
C(9)-C(8)-H(8)	126.4
N(2)-C(9)-C(10)	125.62(18)
N(2)-C(9)-C(8)	109.17(17)
C(10)-C(9)-C(8)	125.14(18)
C(9)-C(10)-C(1)#1	127.52(18)
C(9)-C(10)-C(18)#2	116.99(17)
C(1)#1-C(10)-C(18)#2	115.48(17)
C(12)-C(11)-C(16)	118.15(19)
C(12)-C(11)-C(5)	120.49(18)

C(16)-C(11)-C(5)	121.35(18)
C(13)-C(12)-C(11)	120.6(2)
C(13)-C(12)-H(12)	119.7
C(11)-C(12)-H(12)	119.7
C(14)-C(13)-C(12)	121.3(2)
C(14)-C(13)-H(13)	119.4
C(12)-C(13)-H(13)	119.4
C(13)-C(14)-C(15)	117.9(2)
C(13)-C(14)-C(17)	120.3(2)
C(15)-C(14)-C(17)	121.8(2)
C(16)-C(15)-C(14)	121.2(2)
C(16)-C(15)-H(15)	119.4
C(14)-C(15)-H(15)	119.4
C(15)-C(16)-C(11)	120.8(2)
C(15)-C(16)-H(16)	119.6
C(11)-C(16)-H(16)	119.6
C(14)-C(17)-H(17A)	109.5
C(14)-C(17)-H(17B)	109.5
H(17A)-C(17)-H(17B)	109.5
C(14)-C(17)-H(17C)	109.5
H(17A)-C(17)-H(17C)	109.5
H(17B)-C(17)-H(17C)	109.5
C(19)-C(18)-C(22)	117.46(18)
C(19)-C(18)-C(10)#3	121.63(18)
C(22)-C(18)-C(10)#3	120.89(18)
C(18)-C(19)-C(20)	119.14(19)
C(18)-C(19)-H(19)	120.4
C(20)-C(19)-H(19)	120.4
N(3)-C(20)-C(19)	123.55(19)
N(3)-C(20)-H(20)	118.2
C(19)-C(20)-H(20)	118.2
N(3)-C(21)-C(22)	123.23(19)
N(3)-C(21)-H(21)	118.4
C(22)-C(21)-H(21)	118.4
C(21)-C(22)-C(18)	119.35(19)
C(21)-C(22)-H(22)	120.3
C(18)-C(22)-H(22)	120.3

Symmetry transformations used to generate equivalent atoms:

#1 -x+1,-y,-z #2 -y+2/3,x-y-2/3,z+1/3 #3 -x+y+4/3,-x+2/3,z-1/3

Table S4. Anisotropic displacement parameters ($Å^2 \times 10^3$) for Zn-DpyDtolP-MOF. The anisotropic displacement factor exponent takes the form: $-2p^2[h^2 a^{*2}U^{11} + ... + 2 h k a^* b^* U^{12}]$

	U^{11}	U^{22}	U^{33}	U^{23}	U^{13}	U^{12}
Zn(1)	14(1)	15(1)	15(1)	0(1)	0(1)	9(1)
N(1)	14(1)	16(1)	16(1)	1(1)	0(1)	9(1)
N(2)	14(1)	16(1)	16(1)	2(1)	0(1)	9(1)
N(3)	15(1)	19(1)	17(1)	3(1)	0(1)	8(1)
C(1)	16(1)	15(1)	18(1)	2(1)	2(1)	8(1)
C(2)	22(1)	19(1)	24(1)	-1(1)	-1(1)	13(1)
C(3)	22(1)	19(1)	21(1)	-4(1)	-2(1)	13(1)
C(4)	15(1)	16(1)	17(1)	0(1)	0(1)	8(1)
C(5)	14(1)	17(1)	17(1)	0(1)	1(1)	7(1)
C(6)	16(1)	18(1)	16(1)	0(1)	-1(1)	9(1)
C(7)	21(1)	23(1)	19(1)	-2(1)	-6(1)	12(1)
C(8)	24(1)	25(1)	22(1)	-2(1)	-6(1)	17(1)
C(9)	15(1)	18(1)	19(1)	3(1)	0(1)	10(1)
C(10)	15(1)	16(1)	18(1)	4(1)	2(1)	9(1)
C(11)	17(1)	20(1)	19(1)	0(1)	-3(1)	12(1)
C(12)	20(1)	21(1)	20(1)	0(1)	-1(1)	11(1)
C(13)	26(1)	32(1)	19(1)	-2(1)	-1(1)	18(1)
C(14)	28(1)	29(1)	29(1)	-10(1)	-10(1)	18(1)
C(15)	30(1)	18(1)	34(1)	-6(1)	-9(1)	10(1)
C(16)	25(1)	21(1)	23(1)	1(1)	-3(1)	11(1)
C(17)	51(2)	39(2)	35(1)	-17(1)	-15(1)	24(1)
C(18)	14(1)	19(1)	17(1)	4(1)	1(1)	7(1)
C(19)	16(1)	20(1)	23(1)	-2(1)	1(1)	6(1)
C(20)	17(1)	21(1)	22(1)	0(1)	4(1)	8(1)
C(21)	16(1)	21(1)	19(1)	-1(1)	1(1)	6(1)
C(22)	18(1)	23(1)	17(1)	-1(1)	2(1)	8(1)

Table S5. Hydrogen coordinates (Å × 10^4) and isotropic displacement parameters (Å^2 × 10^3) for Zn-DpyDtolP-MOF.

	x	y	z	U(eq)
H(2)	4638	1339	755	24
H(3)	5102	1391	2929	23
H(7)	5970	465	4719	25
H(8)	6028	-208	3782	26
H(12)	5214	532	5823	24
H(13)	5480	1022	7825	29
H(15)	6301	2123	5265	34
H(16)	6031	1638	3256	28
H(17A)	5854	1820	8814	62
H(17B)	6194	2257	7821	62
H(17C)	6373	1923	8465	62
H(19)	6754	1338	-28	25
H(20)	5962	935	544	25
H(21)	5697	2	-2473	24
H(22)	6478	377	-3158	24

Table S6. The XPS analysis data.

Samples	Zn (at.%)	N-dopant (%)	N1s binding energy (eV)			
			Pyridinic-N (N-6)	Pyrrolic-N (N-5)	Pyridone	Graphitic-N (N-Q)
MDC-700	1.36	5.92	398.3 (56%)	399.4 (24%)	400.5 (15%)	401.4 (5%)
MDC-800	0.72	4.67	398.2 (52%)	399.5 (23%)	400.8 (20%)	401.9 (5%)
MDC-900	0.34	3.63	398.4 (48%)	399.8 (13%)	400.9 (32%)	402.5 (7%)
MDC-700-1KOH	Not detected.	3.15	398.5 (21%)	399.9 (46%)	-	401.4 (33%)
MDC-700-2KOH	Negligible amount	2.32	398.4 (23%)	399.9 (59%)	-	401.7 (17%)

Table S7. The electrochemical parameters of MDC-700 calculated from GCD curves in 6 M KOH electrolyte with 3E system.

Current density (A/g)	Specific capacitance (F/g)	Potential drop (V)	ESR (Ω)	EDLC (F/g)	PC (F/g)	PC (%)	Specific energy (Wh/kg)	Specific power (W/kg)
10	34.3	0.476	950.2	18.0	16.3	47.5	1.3	2625
5	47.3	0.486	1942.2	24.3	23.0	48.6	1.7	1286
3	65.4	0.504	3362.4	32.4	33.0	50.5	2.2	744
2	84.5	0.510	5094.6	41.5	43.0	50.9	2.8	491
1	121.2	0.496	9923.0	61.1	60.1	49.6	4.3	252
0.5	156.6	0.462	18497.8	84.2	72.4	46.2	6.3	134
0.2	195.4	0.406	40683.8	115.9	79.5	40.7	9.6	59

Table S8. The electrochemical parameters of MDC-700-1KOH calculated from GCD curves in 6 M KOH electrolyte with 3E system.

Current density (A/g)	Specific capacitance (F/g)	Potential drop (V)	ESR (Ω)	EDLC (F/g)	PC (F/g)	PC (%)	Specific energy (Wh/kg)	Specific power (W/kg)
50	112.6	0.200	80.4	90.0	22.6	20.1	10.0	19980
30	111.9	0.194	129.4	90.0	21.9	19.5	10.1	12091
20	109.0	0.192	192.6	88.0	21.0	19.3	9.9	8074
10	120.4	0.214	427.4	94.7	25.7	21.3	10.3	3931
5	127.7	0.216	868.4	100.0	27.7	21.7	10.9	1957
3	134.2	0.216	1440.4	105.2	29.0	21.6	11.5	1176
2	139.1	0.214	2147.8	109.2	29.9	21.5	11.9	785
1	146.7	0.214	4289.0	115.3	31.4	21.4	12.6	393
0.5	155.9	0.216	8663.8	122.1	33.8	21.7	13.3	196
0.2	171.6	0.222	22196.4	133.5	38.1	22.2	14.4	78

Table S9. The electrochemical parameters of MDC-700-2KOH calculated from GCD curves in 6 M KOH electrolyte with 3E system.

Current density (A/g)	Specific capacitance (F/g)	Potential drop (V)	ESR (Ω)	EDLC (F/g)	PC (F/g)	PC (%)	Specific energy (Wh/kg)	Specific power (W/kg)
190	269.3	0.154	16.2	228.0	41.3	15.3	26.8	80423
170	277.7	0.142	16.8	238.0	39.7	14.3	28.3	72853
150	280.6	0.144	19.2	240.0	40.6	14.5	28.5	64156
130	283.1	0.144	220.	242.7	40.4	14.3	28.9	55676
100	294.9	0.142	28.2	253.3	41.6	14.1	30.2	42935
50	299.4	0.154	61.4	253.3	46.1	15.4	29.8	21158
40	299.2	0.154	76.6	253.3	45.9	15.3	29.8	16935
30	301.6	0.158	105.2	254.0	47.6	15.8	29.7	12633
20	304.1	0.158	158.2	256.0	48.1	15.8	29.9	8419
10	312.7	0.168	337.0	260.0	52.7	16.9	30.0	4158
2	317.3	0.196	1955.8	255.2	62.1	19.6	28.5	804
1	326.3	0.202	4024.8	260.6	65.7	20.1	28.9	399
0.5	338.7	0.208	8295.6	268.4	70.3	20.8	29.6	198
0.3	350.4	0.212	14161.0	275.9	74.5	21.3	30.2	118
0.2	362.3	0.218	21718.0	283.6	78.7	21.7	30.8	78

Table S10. The electrochemical parameters of MDC-700-1KOH calculated from GCD curves in 1 M Na_2SO_4 electrolyte with 3E system.

Current density (A/g)	Specific capacitance (F/g)	Potential drop (V)	ESR (Ω)	EDLC (F/g)	PC (F/g)	PC (%)	Specific energy (Wh/kg)	Specific power (W/kg)
20	30.2	0.202	20.3	24.0	6.2	20.5	2.7	7973
10	30.8	0.156	31.1	26.0	4.8	15.6	3.0	4222
5	36.1	0.178	70.8	29.7	6.4	17.7	3.4	2057
3	40.5	0.176	117.3	33.4	7.1	17.5	3.8	1236
2	45.3	0.180	179.6	37.2	8.1	17.9	4.2	820
1	53.4	0.174	347.8	44.1	9.3	17.4	5.1	413
0.5	62.8	0.168	678.8	52.2	10.6	16.9	6.0	208
0.3	74.8	0.186	1243.2	60.8	14.0	18.7	6.9	122
0.2	94.7	0.232	2324.3	72.7	22.0	23.2	7.7	77

Table S11. The electrochemical parameters of MDC-700-2KOH calculated from GCD curves in 1 M Na_2SO_4 electrolyte with 3E system.

Current density (A/g)	Specific capacitance (F/g)	Potential drop (V)	ESR (Ω)	EDLC (F/g)	PC (F/g)	PC (%)	Specific energy (Wh/kg)	Specific power (W/kg)
120	148.7	0.194	3.2	120.0	28.7	19.3	13.5	48420
100	149.0	0.106	2.1	133.3	15.7	10.5	16.6	44702
60	153.6	.0116	3.8	136.0	17.6	11.5	16.7	26547
50	152.9	0.084	3.4	140.0	12.9	8.4	17.8	22888
40	151.2	0.082	4.1	138.7	12.5	8.3	17.7	18344
30	152.8	0.070	4.7	142.0	10.8	7.1	18.3	13950
20	152.4	0.056	5.6	144.0	8.4	5.5	18.9	9445
10	151.9	0.048	9.6	144.7	7.2	4.7	19.1	4761
5	153.9	0.044	17.9	147.0	6.9	4.5	19.5	2388
3	156.4	0.048	31.4	149.0	7.4	4.7	19.7	1429
2	159.0	0.050	49.2	151.2	7.8	4.9	20.0	951
1	163.5	0.056	111.7	154.4	9.1	5.6	20.2	472
0.5	172.6	0.066	266.2	161.1	11.5	6.7	20.9	233
0.3	183.7	0.080	539.1	168.9	14.8	8.1	21.6	138
0.2	197.2	0.098	984.5	177.8	19.4	9.8	22.3	90

Table S12. The electrochemical parameters of MDC-700-1KOH calculated from GCD curves in 1 M H_2SO_4 electrolyte with 3E system.

Current density (A/g)	Specific capacitance (F/g)	Potential drop (V)	ESR (Ω)	EDLC (F/g)	PC (F/g)	PC (%)	Specific energy (Wh/kg)	Specific power (W/kg)
100	145.7	0.122	2.4	126.7	19.0	13.0	15.4	43885
50	146.8	0.160	6.4	123.3	23.5	16.0	14.4	21002
30	146.4	0.152	10.2	124.0	22.4	15.3	14.6	12708
20	144.9	0.144	14.4	124.0	20.9	14.4	14.7	8558
10	174.5	0.190	19.0	141.3	33.2	19.0	15.9	4050
5	182.8	0.202	80.56	146.0	36.8	20.1	16.2	1997
3	193.7	0.214	142.2	152.4	41.3	21.3	16.7	1180
2	205.1	0.228	227.2	158.5	46.6	22.7	17.0	773
1	237.8	0.262	523.1	175.6	62.2	26.2	18.0	369
0.5	275.6	0.282	1129.2	197.1	78.5	28.5	19.6	179
0.3	361.1	0.328	2186.1	242.7	118.4	32.8	22.7	101
0.2	589.7	0.436	4367.6	332.0	257.7	43.7	26.0	56

Table S13. The electrochemical parameters of MDC-700-2KOH calculated from GCD curves in 1 M H_2SO_4 electrolyte with 3E system.

Current density (A/g)	Specific capacitance (F/g)	Potential drop (V)	ESR (Ω)	EDLC (F/g)	PC (F/g)	PC (%)	Specific energy (Wh/kg)	Specific power (W/kg)
150	201.9	0.258	3.4	150.0	51.9	25.7	15.5	55725
100	213.6	0.252	5.0	160.0	53.6	25.1	16.6	37448
70	222.6	0.246	7.0	168.0	54.6	24.5	17.6	26410
60	222.4	0.226	7.6	172.0	50.4	22.7	18.5	23191
50	226.9	0.206	8.2	180.0	46.9	20.7	19.8	19837
30	227.8	0.200	13.4	182.0	45.8	20.1	20.2	11987
20	234.6	0.198	19.9	188.0	46.6	19.9	20.9	8014
10	250.6	0.194	38.8	202.0	48.6	19.4	22.6	4030
5	268.9	0.192	77.2	217.0	51.9	19.3	24.3	2018
3	282.7	0.192	127.6	228.6	54.1	19.1	25.7	1213
2	295.9	0.192	192.5	238.9	57.0	19.3	26.8	807
1	321.7	0.200	398.7	257.6	64.1	19.9	28.6	400
0.5	371.9	0.216	864.0	291.6	80.3	21.6	31.8	196
0.3	445.0	0.242	1607.8	337.6	107.4	24.1	35.6	114
0.2	555.6	0.276	2774.4	402.0	153.6	27.6	40.4	72

Table S14. The specific energy and specific power values of reported carbon samples in KOH aqueous electrolytes with 3E system.

Sample	Electrolyte	Potential window (V)	E_{max} (Wh/kg)	E_{min} (Wh/kg)	P_{max} (W/kg)	Reference
3D-HPCFs	6 M KOH	1.1	63.7	18.7	11000	7a
DWNT	6 M KOH	1.0	12.1	10.0	7200	7b
ACN	6 M KOH	1.0	63.2	47.5	34200	
BP-800	6 M KOH	1.0	~41.7	~6.9	5000	7c
YP17D	6 M KOH	1.0	~29.9	17.6	31750	7d
a-CBP	6 M KOH	1.0	41.1	30.7	55250	
CS15A6	6 M KOH	0.9	25.1	18.2	3645	7e
PGC-1	6 M KOH	1.0	16.4	13.2	9500	7f
APC	6 M KOH	1.0	32.1	19.3	6950	7g
ACA-500/500-K1	30 % KOH	1.0	34.0	27.9	10050	7h
RGO	1 M KOH	1.1	33.8	28.9	94655	7i
N-RGO	1 M KOH	1.1	30.6	25.8	84590	
MDC-700-1KOH	6 M KOH	1.0	14.4	10.0	19980	This work
MDC-700-2KOH	6 M KOH	1.0	30.8	26.8	80423	This work

Table S15. The specific energy and specific power values of reported carbon samples in H_2SO_4 aqueous electrolytes with 3E system.

Sample	Electrolyte	Potential window (V)	E_{max} (Wh/kg)	E_{min} (Wh/kg)	P_{max} (W/kg)	Reference
CNC700	1 M H_2SO_4	1.0	32.5	23.6	8500	8a
Activated carbon	0.5 M H_2SO_4	1.0	~20.6	~17.5	~6300	8b
Z-900	0.5 M H_2SO_4	1.2	42.8	23.0	6900	8c
NPC-800	1 M H_2SO_4	0.8	21.2	15.2	13680	8d
PCF-4	1 M H_2SO_4	0.8	37.4	18.6	14725	8e
N-doped carbon nanosheets	0.5 M H_2SO_4	1.0	14.2	~11.3	~40800	8f
NPC	1 M H_2SO_4	0.9	28.4	14.1	56250	8g
RGO	1 M H_2SO_4	1.1	31.9	26.1	85525	7h
N-RGO	1 M H_2SO_4	1.1	36.6	26.4	86350	
MDC-700-1KOH	1 M H_2SO_4	1.0	26.0	15.4	43855	This work
MDC-700-2KOH	1 M H_2SO_4	1.0	40.4	15.5	55735	This work

Table S16. The estimated electrochemical parameters from capacitance versus frequency plots and the fitted values from Nyquist plots using the equivalent circuits for various carbon samples in 6 M KOH aqueous electrolyte with 3E system.

Parameters	MDC-700		MDC-700-1KOH		MDC-700-2KOH	
	Before 1000 cycles	After 1000 cycles	Before 30000 cycles	After 30000 cycles	Before 30000 cycles	After 30000 cycles
Maximum C at 0.01 Hz (F/g)	46.6	55.5	108.7	102.9	244.4	240.4
Operating frequency (Hz)	0.04948	0.05995	3.831	3.831	1.896	1.896
Relaxation time constant (s)	20.2102	16.6806	0.2610	0.2610	0.5274	0.5274
R1 (Ω)	10.99	10.72	4.669	4.809	4.178	4.116
C1 (F/g)	88.8	58.6	0.651	0.740	1.05	1.01
R2 (Ω)	152.8	24.7	0.32320	0.29855	0.42333	0.51322
C2 (F/g)	19.3	16.7	305.1	285.6	701.6	1004.9
R3 (Ω)	32.93	8.607	1.296	1.654	1.346	1.968
W1-R (Ω)	9.791	13.25	2.198	2.166	3.797	4.058
WI-T (s)	6.4502×10^{-4}	1.5398×10^{-3}	8.6671×10^{-3}	8.2970×10^{-3}	4.2942×10^{-2}	4.1384×10^{-2}
WI-P	0.31396	0.32020	0.47781	0.47928	0.49307	0.48533

Table S17. The estimated values from capacitance versus frequency plots and the fitted values from Nyquist plots using the equivalent circuit for MDC-700-1KOH and MDC-700-2KOH in 1 M Na_2SO_4 aqueous electrolyte with 3E system.

Parameters	MDC-700-1KOH	MDC-700-2KOH	
		Before 30000 cycles	After 30000 cycles
Maximum C at 0.01 Hz (F/g)	23.9	153.6	120.0
Operating frequency (Hz)	0.3162	0.8254	1
Relaxation time constant (s)	3.1626	1.2115	1
R1 (Ω)	15.53	16.54	16.77
C1 (F/g)	0.612	0.430	0.399
R2 (Ω)	8.196	0.93023	1.141
C2 (F/g)	125.6	171.5	182.0
R3 (Ω)	2003	5.144	9804
W1-R (Ω)	316.5	7.749	26.2
WI-T (s)	2.0678×10^{-1}	5.5714×10^{-2}	3.7108×10^{-1}
WI-P	0.40611	0.49418	0.47513

Table S18. The estimated values from capacitance versus frequency plots and the fitted values from Nyquist plots using the equivalent circuits for MDC-700-1KOH and MDC-700-2KOH in 1 M H_2SO_4 aqueous electrolyte with 3E system.

Parameters	MDC-700-1KOH		MDC-700-2KOH	
	Before 30000 cycles	After 30000 cycles	Before 30000 cycles	After 30000 cycles
Maximum C at 0.01 Hz (F/g)	134.7	140.6	229.4	238.2
Operating frequency (Hz)	1.668	1.778	0.7263	0.7743
Relaxation time constant (s)	0.5995	0.5624	1.3768	1.2915
R1 (Ω)	10.1	8.674	9.114	9.661
C1 (F/g)	155.2	1.60	0.249	1.55
R2 (Ω)	1.224	0.30731	0.65612	0.87502
C2 (F/g)	448.0	171.7	2.68	1000.4
R3 (Ω)	1.334	2.016	0.7565	3.707
W1-R (Ω)	2.211	3.046	14.06	10.12
WI-T (s)	1.2168×10^{-2}	1.8073×10^{-2}	1.1735×10^{-1}	9.7112×10^{-2}
WI-P	0.48549	0.48724	0.46639	0.47779

Table S19. The electrochemical recycling performances of the reported carbon samples in 6 M KOH aqueous electrolyte with 3E system.

Sample	Potential window (V)	Recycling condition (A/g)	Initial C_s (F/g)	Cycle number	Retention (%)	Reference
HPCFA	1.0	10	144.2	10000	95.0	9a
N-OMCS	1.0	20	~200.0	25000	100.0	9b
NHCSF-0	1.0	4	~200.0	20000	89.9	9c
NHCSF-3	1.0	4	~250.0	20000	92.1	
NMCS-8	1.0	10	~200.0	10000	96.0	9d
KBM-700	0.8	10	~175	10000	97.0	9e
carbon–Zn–Mg-900	1.0	20	162.9	10000	96.6	9f
MDC-700-1KOH	1.0	50	118.7	30000	96.5	This work
MDC-700-2KOH	1.0	20	282.5	30000	97.2	This work

Table S20. The electrochemical recycling performances of the reported carbon samples in 1 M H_2SO_4 aqueous electrolyte with 3E system.

Sample	Potential window (V)	Recycling condition (A/g)	Initial C_s (F/g)	Cycle number	Retention (%)	Reference
CA-C-1	1.0	1	~275.0	5000	97.0	10a
ZIF-8-NPC	1.0	1	201.0	5000	97.7	10b
NPCF	1.0	1	332.0	5000	98.9	
CS3-6A	0.8	10	~280.0	8000	98.0	10c
N-OMC	0.8	2	156.0	10000	107.5	10d
GA-MC	0.8	2	168.0	5000	141.7	10e
N-OMCNFA	0.8	3	~240.0	10000	~100.0	10f
MDC-700-1KOH	1.0	50	142.0	30000	105.6	This work
MDC-700-2KOH	1.0	20	237.3	30000	105.0	This work

Table S21. The electrochemical parameters of MDC-700-2KOH calculated from GCD curves in 1 M KOH electrolyte with Swagelok cell-based 2E system.

Current density (A/g)	Specific capacitance (F/g)	Potential drop (V)	ESR (Ω)	EDLC (F/g)	PC (F/g)	PC (%)	Specific energy (Wh/kg)	Specific power (W/kg)
16.4	157.3	0.196	3.9	131.6	25.7	16.3	5.5	8256
9.87	158.9	0.157	5.2	138.2	20.7	13.0	6.0	5148
4.93	164.9	0.099	6.6	151.3	13.6	8.2	6.9	2717
3.95	169.3	0.093	7.8	156.1	13.2	7.8	7.2	2184
2.96	169.5	0.082	9.1	157.9	11.6	6.8	7.4	1655
1.97	172.5	0.074	12.3	161.8	10.74	6.2	7.6	1111
0.987	176.2	0.061	20.2	167.3	8.9	5.1	7.9	562
0.493	180.5	0.057	38.0	171.9	8.6	4.8	8.2	282
0.329	183.2	0.057	57.1	174.5	8.7	4.7	8.3	188
0.197	188.5	0.060	100.5	179.1	9.4	5.0	8.5	113

Table S22. The electrochemical parameters of MDC-700-2KOH calculated from GCD curves in 1 M Na_2SO_4 electrolyte with Swagelok cell-based 2E system.

Current density (A/g)	Specific capacitance (F/g)	Potential drop (V)	ESR (Ω)	EDLC (F/g)	PC (F/g)	PC (%)	Specific energy (Wh/kg)	Specific power (W/kg)
14.3	69.5	0.450	11.2	50.5	19.0	27.3	3.2	8216
10.7	76.0	0.360	12.0	58.9	17.1	22.5	4.1	6642
5.36	88.3	0.290	19.3	72.3	16.0	18.1	5.3	3510
4.29	90.6	0.276	23.0	75.0	15.6	17.2	5.5	2838
3.21	91.9	0.257	28.6	77.1	14.8	16.1	5.8	2158
2.14	94.7	0.212	35.3	82.1	12.6	13.3	6.3	1488
1.07	101.6	0.171	55.3	90.5	11.1	10.9	7.2	763
0.536	111.6	0.175	116.9	99.4	12.2	10.9	7.9	382
0.357	120.2	0.191	190.7	105.8	14.4	12.0	8.3	252
0.214	138.2	0.236	392.9	117.8	20.4	14.8	8.9	146

Table S23. The specific energy and specific power values of the reported carbon samples in KOH aqueous electrolytes with 2E system.

Sample	Electrolyte	Potential window (V)	E_{max} (Wh/kg)	E_{min} (Wh/kg)	P_{max} (W/kg)	Reference
N-CNFs-900	6 M KOH	1.0	7.1	5.4	15000	11a
Carbon	6 M KOH	1.6	7.1	~4.4	~4000	11b
Maxsorb	30 wt% KOH	0.9	9.4	2.1	410	11c
OMC-1		0.9	6.0	3.9	789	
OMC-2		0.9	5.3	4.7	932	
OMC-3		0.9	8.0	5.1	1019	
MC-A	6 M KOH	1.0	7.2	~6.4	~2300	11d
MPC-A		1.0	6.8	~5.8	~2100	
MAC-A		1.0	9.4	~6.1	~2200	
KPAC-800	6 M KOH	1.0	8.2	4.7	13000	11e
APC	6 M KOH	1.4	18.4	11.4	6881	7g
NNPC-800	6 M KOH	1.0	5.4	~3.0	~1000	11f
HPCNC-700-a	6 M KOH	1.0	9.7	~6.3	4200	11g
MAC-2-700	6 M KOH	1.0	8.5	5.0	12080	11h
MDC-700-2KOH	1 M KOH	1.2	8.5	5.5	8256	This work

Table S24. The electrochemical recycling performances of the reported carbon samples in KOH aqueous electrolytes with 2E system.

Sample	Potential window (V)	Recycling condition (A/g)	Initial C_s (F/g)	Cycle number	Retention (%)	Reference
CNCs-800	1.0	2	~240	5000	95.0	12a
LN750	1.2	1	168	5000	89.0	12b
	1.4	1	163	5000	86.0	
APC	1.4	0.5	270	5000	91.1	7g
HPCNC-700-a	1.0	5	~270	5000	85.0	11g
HCN-900-10H5R	1.0	1	180	5000	~91.7	12c
MDC-700-2KOH	1.2	4.93	166.1	5000	89.8	This work

Table S25. The estimated values from capacitance versus frequency plots and the fitted values from Nyquist plots using the equivalent circuit for MDC-700-2KOH in different electrolytes with Swagelok cell-based 2E system.

Sample	1 M KOH		1 M Na_2SO_4	
	Before 5000 cycles	After 5000 cycles	Before 3000 cycles	After 3000 cycles
Maximum C at 0.01 Hz (F/g)	174.3	145.5	67.8	61.0
Operating frequency (Hz)	0.3831	0.4948	0.3371	0.2966
Relaxation time constant (s)	2.610	2.021	2.966	3.372
R1 (Ω)	0.56754	0.55994	1.313	1.429
CPE1-T (F/g)	29.1	26.8	34.0	46.8
CPE-P	0.95987	0.96962	0.90082	0.86086
R2 (Ω)	2.058	1.783	4.846	6.266
W1-R (Ω)	1.098	0.93314	5.353	5.448
WI-T (s)	0.1212	0.084987	0.1859	0.16061
WI-P	0.47967	0.47984	0.45990	0.45622

Table S26. The electrochemical parameters of MDC-700-2KOH calculated from GCD curves in 2 M H_2SO_4 electrolyte with Ti plate-based 2E system.

Current density (A/g)	Specific capacitance (F/g)	Potential drop (V)	ESR (Ω)	EDLC (F/g)	PC (F/g)	PC (%)	Specific energy (Wh/kg)	Specific power (W/kg)
15	115.2	0.271	7.1	84.0	31.2	27.1	2.1	5468
10	128.7	0.254	11.7	96.0	32.7	25.4	2.5	3730
7	140.3	0.242	15.7	106.4	33.9	24.2	2.8	2654
5	151.8	0.218	20.2	118.7	33.1	21.8	3.2	1955
4	157.1	0.213	24.6	123.7	33.4	21.3	3.4	1575
3	167.1	0.201	31.0	133.6	33.5	20.0	3.7	1199
2	179.1	0.184	42.6	146.1	33.0	18.4	4.1	816
1	203.4	0.174	80.6	168.0	35.4	17.4	4.8	413
0.5	236.6	0.191	176.7	191.5	45.1	19.1	5.4	202
0.3	283.0	0.229	353.9	218.1	64.9	22.9	5.8	116
0.2	369.2	0.301	697.1	257.9	111.3	30.1	6.3	70

Table S27. The electrochemical parameters of MDC-700-2KOH calculated from GCD curves in 2 M H_2SO_4 electrolyte with ITO glass-based 2E system.

Current density (A/g)	Specific capacitance (F/g)	Potential drop (V)	ESR (Ω)	EDLC (F/g)	PC (F/g)	PC (%)	Specific energy (Wh/kg)	Specific power (W/kg)
4	56.9	0.662	55.9	19.2	37.7	66.3	0.2	675
3	93.0	0.587	66.1	38.4	54.6	58.7	0.6	619
2	113.0	0.448	75.6	62.4	50.6	44.8	1.2	552
1	138.6	0.296	99.9	97.6	41.0	29.6	2.4	352
0.5	173.4	0.252	170.3	130.0	43.4	25.0	3.4	187
0.3	225.2	0.280	315.6	162.1	63.1	28.0	4.1	108
0.2	379.8	0.396	669.0	228.9	150.9	39.7	4.8	60

Table S28. The specific energy and specific power values of the reported MOF-derived carbon samples in H_2SO_4 aqueous electrolytes with 2E system.

Sample	Electrolyte	Potential window (V)	E_{max} (Wh/kg)	E_{min} (Wh/kg)	P_{max} (W/kg)	Reference
CIRMOF-3-950	1 M H_2SO_4	1.0	8.3	5.8	2075	13a
NPC_{1000}	1 M H_2SO_4	1.0	4.2	3.8	688	13b
A-ZC-800	1 M H_2SO_4	1.0	5.9	4.9	1763	13c
AS-ZC-800			7.4	6.5	9350	
NCPPs	1 M H_2SO_4	1.0	6.6	~2.0	~1400	13d
HPCNFs-N	2 M H_2SO_4	1.0	11.0	6.7	25000	13e
NPC	1 M H_2SO_4	1.0	10.8	5.5	994	13f
C800	1 M H_2SO_4	1.0	6.5	5.6	1000	13g
MPC	1 M H_2SO_4	1.0	3.8	2.5	3550	13h
CNRod			5.7	4.0	5800	
GNRib			6.7	4.3	6150	
MDC-700-2KOH	2 M H_2SO_4	1.0	6.3	2.1	5468	This work

Table S29. The electrochemical recycling performances of reported carbon samples in H_2SO_4 aqueous electrolyte with 2E system under the identical potential window and cycle number.

Sample	Potential window (V)	Recycling condition (A/g)	Initial C_s (F/g)	Cycle number	Retention (%)	Reference
SSG Film	1.0	108	~180	10000	97.0	14a
3D-porous RGO films	1.0	25	~230	10000	97.6	14b
RGO films		25	~125		86.2	
LN750	1.0	1	~180	10000	83.3	12b
SSC-1	1.0	5	~150	10000	72.0	14c
MDC-700-2KOH	1.0	3	172.1	10000	78.5	This work
MDC-700-2KOH	1.0	2	133.9	10000	101.0	This work

Table S30. The estimated values from capacitance versus frequency plots and the fitted values from Nyquist plots using the equivalent circuit for MDC-700-2KOH in 2 M H_2SO_4 electrolyte with Ti plate-based and ITO glass-based 2E systems.

Sample	Ti plate current collector		ITO glass current collector	
	Before 10000 cycles	After 10000 cycles	Before 10000 cycles	After 10000 cycles
Maximum C at 0.01 Hz (F/g)	134.7	119.1	116.1	117.1
Operating frequency (Hz)	0.2448	0.1565	0.04948	0.04642
Relaxation time constant (s)	4.085	6.390	20.210	21.542
R1 (Ω)	0.31784	0.25351	24.42	26.37
CPE1-T (F/g)	16.5	25.0	48.2	50.2
CPE-P	0.95015	0.98007	0.70500	0.66119
R2 (Ω)	3.559	4.020	10.16	8.317
W1-R (Ω)	1.820	6.464	86.35	89.62
WI-T (s)	0.076298	0.29098	64.06	70.74
WI-P	0.32409	0.26324	0.69452	0.69200
C1 (F/g)	180.7	149.8	153.4	154.8

References

[1] J. Gao, X. Wang, Y. Zhang, J. Liu, Q. Lu, M. Liu, *Electrochim. Acta*, **2016**, *207*, 266-274.

[2] a) Y. F. Nie, Q. Wang, X. Y. Chen, Z. J. Zhang, *J. Power Sources*, **2016**, *320*, 140-152; b) Y. Zhang, X. Cui, L. Zu, X. Cai, Y. Liu, X. Wang, H. Lian, *Materials*, **2016**, *9*, 734-746; c) L.-Q. Mai, A. Minhas-Khan, X. Tian, K. M. Hercule, Y.-L. Zhao, X. Lin, X. Xu, *Nat. Commun.*, **2013**, *4*, 2923-2929.

[3] a) J.-W. Lang, X.-B. Yan, X.-Y. Yuan, J. Yang, Q.-J. Xue, *J. Power Sources*, **2011**, *196*, 10472-10478; b) G. Xu, C. Zheng, Q. Zhang, J. Huang, M. Zhao, J. Nie, X. Wang, F. Wei, *Nano Res.*, **2011**, *4*, 870-881.

[4] J. Lee, P. Srimuk, S. Fleischmann, X. Su, T. Alan Hatton, V. Presser, *Prog. Mater. Sci.*, **2019**, *101*, 46-89.

[5] J. Chmiola, G. Yushin, R. Dash, Y. Gogotsi, *J. Power Sources*, **2006**, *158*, 765-772.

[6] J. Zhang, J. Jiang, H. Li, X. S. Zhao, *Energy Environ. Sci.*, **2011**, *4*, 4009-4015.

[7] a) B. You, J. Jiang, S. Fan, *ACS Appl. Mater. Interfaces*, **2014**, *6*, 15302−15308; b) Z. Fan, J. Yan, T. Wei, L. Zhi, G. Ning, T. Li, F. Wei, *Adv. Funct. Mater.*, **2011**, *21*, 2366-2375; c) H. Zhu, J. Yin, X. Wang, H. Wang, X. Yang, *Adv. Funct. Mater.*, **2013**, *23*, 1305-1312; d) C. Long, D. Qi, T. Wei, J. Yan, L. Jiang, Z. Fan, *Adv. Funct. Mater.*, **2014**, *24*, 3953-3961; e) K. Xia, Q. Gao, J. Jiang, J. Hu, *Carbon*, **2008**, *46*, 1718-1726; f) Z. Wang, X. Zhang, X. Liu, M. Lv, K. Yang, J. Meng, *Carbon*, **2011**, *49*, 161-169; g) Z.-X. Li, B.-L. Yang, K.-Y. Zou, L. Kong, M.-L. Yue, H.-H. Duan, *Carbon*, **2019**, *144*, 540-548; h) J. Wang, X. Yang, D. Wu, R. Fu, M. S. Dresselhaus, G. Dresselhaus, *J. Power Sources*, **2008**, *185*, 589-594; i) Y.-H. Lee, K.-H. Chang, C.-C. Hu, *J. Power Sources*, **2013**, *227*, 300-308.

[8] a) K. Xie, X. Qin, X. Wang, Y. Wang, H. Tao, Q. Wu, L. Yang, Z. Hu, *Adv. Mater.*, **2012**, *24*, 347-352; b) S. Isikli, M. Lecea, M. Ribagorda, M. C. Carreño, R. Díaz, *Carbon*, **2014**, *66*, 654-661; c) W. Chaikittisilp, M. Hu, H. Wang, H.-S. Huang, T. Fujita, K. C.-W. Wu, L.-C. Chen, Y. Yamauchi, K. Ariga, *Chem. Commun.*, **2012**, *48*, 7259-7261; d) N. L. Torad, R. R. Salunkhe, Y. Li, H. Hamoudi, M. Imura, Y. Sakka, C.-C. Hu, Y. Yamauchi, *Chem. Eur. J.*, **2014**, *20*, 7895-7900; e) Z. Zhang, Z. Zhou, H. Peng, Y. Qin, G. Li, *Electrochim. Acta*, **2014**, *134*, 471-477; f) G. Hasegawa, M. Aoki, K. Kanamori, K. Nakanishi, T. Hanada,

K. Tadanaga, *J. Mater. Chem.*, **2011**, *21*, 2060-2063; g) R. R. Salunkhe, Y. Kamachi, N. L. Torad, S. M. Hwang, Z. Sun, S. X. Dou, J. H. Kim, Y. Yamauchi, *J. Mater. Chem. A*, **2014**, *2*, 19848-19854.

[9] a) Y. Liu, G. Li, Y. Guo, Y. Ying, X. Peng, *ACS Appl. Mater. Interfaces*, **2017**, *9*, 14043-14050; b) J.-G. Wang, H. Liu, H. Sun, W. Hua, H. Wang, X. Liu, B. Wei, *Carbon*, **2018**, *127*, 85-91; c) Z. Li, H. Mi, L. Liu, Z. Bai, J. Zhang, Q. Zhang, J. Qiu, *Carbon*, **2018**, *136*, 176-186; d) Y.-N. Hou, Z. Zhao, Z. Yu, S. Zhang, S. Li, J. Yang, H. Zhang, C. Liu, Z. Wang, J. Qiu, *Chem. Eur. J.*, **2018**, *24*, 2681-2686; e) Y. Pan, Y. Zhao, S. Mu, Y. Wang, C. Jiang, Q. Liu, Q. Fang, M. Xue, S. Qiu, *J. Mater. Chem. A*, **2017**, *5*, 9544-9552; f) Z. J. Zhang, D. H. Xie, P. Cui, X. Y. Chen, *RSC Adv.*, **2014**, *4*, 6664-6671.

[10] a) M. Zhou, F. Pu, Z. Wang, S. Guan, *Carbon*, **2014**, *68*, 185-194; b) C. Wang, C. Liu, J. Li, X. Sun, J. Shen, W. Han, L. Wang, *Chem. Commun.*, **2017**, *53*, 1751-1754; c) N. P. Wickramaratne, J. Xu, M. Wang, L. Zhu, L. Dai, M. Jaroniec, *Chem. Mater.*, **2014**, *26*, 2820-2828; d) H. Chen, M. Zhou, Z. Wang, S. Zhao, S. Guan, *Electrochim. Acta*, **2014**, *148*, 187-194; e) Z.-S. Wu, Y. Sun, Y.-Z. Tan, S. Yang, X. Feng, K. Müllen, *J. Am. Chem. Soc.*, **2012**, *134*, 19532-19535; f) D.-D. Zhou, W.-Y. Li, X.-L. Dong, Y.-G. Wang, C.-X. Wang, Y.-Y. Xia, *J. Mater. Chem. A*, **2013**, *1*, 8488-8496.

[11] a) L.-F. Chen, X.-D. Zhang, H.-W. Liang, M. Kong, Q.-F. Guan, P. Chen, Z.-Y. Wu, S.-H. Yu, *ACS Nano*, **2012**, *6*, 7092-7102; b) R. R. Salunkhe, J. Tang, Y. Kamachi, T. Nakato, J. H. Kim, Y. Yamauchi, *ACS Nano*, **2015**, *9*, 6288-6296; c) W. Xing, S. Z. Qiao, R. G. Ding, F. Li, G. Q. Lu, Z. F. Yan, H. M. Cheng, *Carbon*, **2006**, *44*, 216-224; d) J. Hu, H. Wang, Q. Gao, H. Guo, *Carbon*, **2010**, *48*, 3599-3606; e) P. Cheng, S. Gao, P. Zang, X. Yang, Y. Bai, H. Xu, Z. Liu, Z. Lei, *Carbon*, **2015**, *93*, 315-324; f) J. Zou, P. Liu, L. Huang, Q. Zhang, T. Lan, S. Zeng, X. Zeng, L. Yu, S. Liu, H. Wu, W. Tu, Y. Yao, *Electrochim. Acta*, **2018**, *271*, 599-607; g) J. Shao, M. Song, G. Wu, Y. Zhou, J. Wan, X. Ren, F. Ma, *Energy Storage Mater.*, **2018**, *13*, 57-65; h) D. Zhang, C. He, J. Zhao, J. Wang, K. Li, *J. Colloid Interf. Sci.*, **2019**, *546*, 101-112.

[12] a) Y. Tan, C. Xu, G. Chen, Z. Liu, M. Ma, Q. Xie, N. Zheng, S. Yao, *ACS Appl. Mater. Interfaces*, **2013**, *5*, 2241-2248; b) M. P. Bichat, E. Raymundo-Piñero, F. Béguin, *Carbon*, **2010**, *48*, 4351-4361; c) F. Xu, Z. Tang, S. Huang, L. Chen, Y. Liang, W. Mai, H. Zhong, R. Fu, D. Wu, *Nat. Commun.*, **2015**, *6*, 7221-7230.

[13] a) J.-W. Jeon, R. Sharma, P. Meduri, B. W. Arey, H. T. Schaef, J. L. Lutkenhaus, J. P. Lemmon, P. K. Thallapally, M. I. Nandasiri, B. P. McGrail, S. K. Nune, *ACS Appl. Mater. Interfaces*, **2014**, *6*, 7214-7222; b) B. Liu, H. Shioyama, H. Jiang, X. Zhang, Q. Xu, *Carbon*, **2010**, *48*, 456-463; c) A. J. Amali, J.-K. Sun, Q. Xu, *Chem. Commun.*, **2014**, *50*, 1519-1522; d) L. Kong, Q. Chen, X. Shen, Z. Xu, C. Xu, Z. Ji, J. Zhu, *Electrochim. Acta*, **2018**, *265*, 651-661; e) L.-F. Chen, Y. Lu, L. Yu, X. W. Lou, *Energy Environ. Sci.*, **2017**, *10*, 1777-1783; f) B. Liu, H. Shioyama, T. Akita, Q. Xu, *J. Am. Chem. Soc.*, **2008**, *130*, 5390-5391; g) H.-L. Jiang, B. Liu, Y.-Q. Lan, K. Kuratani, T. Akita, H. Shioyama, F. Zong, Q. Xu, *J. Am. Chem. Soc.*, **2011**, *133*, 11854-11857; h) P. Pachfule, D. Shinde, M. Majumder, Q. Xu, *Nat. Chem.*, **2016**, *8*, 718-723.

[14] a) X. Yang, J. Zhu, L. Qiu, D. Li, *Adv. Mater.*, **2011**, *23*, 2833-2838; b) Y. Shao, M. F. El-Kady, C.-W. Lin, G. Zhu, K. L. Marsh, J. Y. Hwang, Q. Zhang, Y. Li, H. Wang, R. B. Kaner, *Adv. Mater.*, **2016**, *28*, 6719-6726; c) T. E. Rufford, D. Hulicova-Jurcakova, K. Khosla, Z. Zhu, G. Q. Lu, *J. Power Sources*, **2010**, *195*, 912-918.
